# The Role of bZIP Transcription Factors in Green Plant Evolution: Adaptive Features Emerging from Four Founder Genes

**DOI:** 10.1371/journal.pone.0002944

**Published:** 2008-08-13

**Authors:** Luiz Gustavo Guedes Corrêa, Diego Mauricio Riaño-Pachón, Carlos Guerra Schrago, Renato Vicentini dos Santos, Bernd Mueller-Roeber, Michel Vincentz

**Affiliations:** 1 Centro de Biologia Molecular e Engenharia Genética, Departamento de Genética e Evolução, Instituto de Biologia, Universidade Estadual de Campinas, Campinas, Brazil; 2 Department of Molecular Biology, University of Potsdam, Potsdam-Golm, Germany; 3 Cooperative Research Group, Max-Planck Institute of Molecular Plant Physiology, Potsdam-Golm, Germany; 4 GabiPD Team, Bioinformatics Group, Max-Planck Institute of Molecular Plant Physiology, Potsdam-Golm, Germany; 5 Laboratório de Biodiversidade Molecular, Departamento de Genética, Universidade Federal do Rio de Janeiro, Rio de Janeiro, Brazil; Michigan State University, United States of America

## Abstract

**Background:**

Transcription factors of the basic leucine zipper (bZIP) family control important processes in all eukaryotes. In plants, bZIPs are regulators of many central developmental and physiological processes including photomorphogenesis, leaf and seed formation, energy homeostasis, and abiotic and biotic stress responses. Here we performed a comprehensive phylogenetic analysis of bZIP genes from algae, mosses, ferns, gymnosperms and angiosperms.

**Methodology/Principal Findings:**

We identified 13 groups of bZIP homologues in angiosperms, three more than known before, that represent 34 Possible Groups of Orthologues (PoGOs). The 34 PoGOs may correspond to the complete set of ancestral angiosperm bZIP genes that participated in the diversification of flowering plants. Homologous genes dedicated to seed-related processes and ABA-mediated stress responses originated in the common ancestor of seed plants, and three groups of homologues emerged in the angiosperm lineage, of which one group plays a role in optimizing the use of energy.

**Conclusions/Significance:**

Our data suggest that the ancestor of green plants possessed four bZIP genes functionally involved in oxidative stress and unfolded protein responses that are bZIP-mediated processes in all eukaryotes, but also in light-dependent regulations. The four founder genes amplified and diverged significantly, generating traits that benefited the colonization of new environments.

## Introduction

Growth and development of all organisms depend on proper regulation of gene expression. The control of transcription initiation rates by transcription factors (TF) represents one of the most important means of modulating gene expression [1]–[4]. TFs can be grouped into different protein families according to their primary and/or three-dimensional structure similarities in the DNA-binding and multimerization domains [4]–[6]. The interplay between the amplification of the ancestral repertoire of TFs, the emergence of new TFs, the combination of protein domains and sequence divergence constitutes an important driving force towards the evolution of organismic complexity [7]–[10]. Understanding the detailed evolutionary history of these TFs and their corresponding functions is therefore crucial to reveal the changes and/or innovations in transcriptional regulatory circuits that underlie the biological diversity found among eukaryotes.

Large scale genomic comparisons revealed that angiosperm TF families undergo more intense gene expansion when compared to animals and fungi, possibly reflecting the ability of flowering plants to efficiently adapt to different and unstable environmental conditions. Moreover, gene expansion rates vary among plant TF families, indicating lineage-differential specializations [11], [12]. For instance, MADS-box and homeodomain families, which exert similar functions in developmental control, expanded preferentially in the angiosperm and human lineages, respectively [13], [14]. Contrariwise, the basic leucine zipper (bZIP) TF family apparently expanded to a similar extent in angiosperms and humans [15]. Currently we do not well understand why individual TF families underwent differential evolutionary expansions in the different eukaryotic lineages. Therefore, a deep evolutionary analysis of TF families including the identification of the founding (ancestral) gene sets in combination with functional assignments will greatly assist in addressing this issue [16], [17].

To our knowledge, however, only four families that are present in all green plants have until today been studied in a deep evolutionary scale, Dof [18], homeodomain [19], MADS-box [20], [21] and WRKY [22]. As a matter of fact, groups of orthologues, for which functional equivalence is often assumed, are rarely identified in a systematic and direct manner, with the exception of the HD-Zip class III subfamily [23], [24]. It is thus often difficult to infer ancestral functions at different time points of the evolutionary process. Here we performed a comprehensive analysis of the evolutionary relationships of TFs of the green plant bZIP family; homologous and orthologous relationships among bZIP TFs were established and ancestral functions were inferred.

The bZIP TFs are characterized by a 40- to 80-amino-acid-long conserved domain (bZIP domain) that is composed of two motifs: a basic region responsible for specific binding of the TF to its target DNA, and a leucine zipper required for TF dimerization [5], [25]. Genetic, molecular and biochemical analyses indicate that bZIPs are regulators of important plant processes such as organ and tissue differentiation [26]–[30], cell elongation [31], [32], nitrogen/carbon balance control [33], [34], pathogen defence [35]–[40], energy metabolism [41], unfolded protein response [42], [43], hormone and sugar signalling [44]–[47], light response [48]–[50], osmotic control [34], [51], and seed storage protein gene regulation [52]. Initially, 50 plant bZIP proteins were classified into five families, taking into account similarities of their bZIP domain [53]. An original investigation of the complete *Arabidopsis thaliana* genome sequence indicated the presence of 81 putative *bZIP* genes [54], [55]. However, further detailed studies revealed 75 to 77 bZIP proteins to be encoded by the Arabidopsis nuclear genome, representing members of ten groups of homologues [55], [56].

The availability of the rice (*Oryza sativa*) [57], [58], black cottonwood (*Populus trichocarpa*) [59] and Arabidopsis genomic sequences [54] provides an exciting opportunity for the large-scale investigation of the genetic bases that underlies the extensive physiological and morphological diversity amongst the two main angiosperm divisions: monocots and eudicots. A possible comparative approach involves the establishment of relationships between different genomes in a homologous gene system [60]–[62], in which each group of orthologues is derived from an ancestral gene that underwent numerous modifications throughout evolution, including duplication and subsequent functional diversification. Considering that all genes of a given group of orthologues have the same ancestral origin, the establishment of this classification should allow the transfer of biochemical, structural and functional information from one protein to another, inside the same group [63]. Moreover, the relationships within a group of orthologues constitute the basis for a better understanding of the evolution of ancestral functions (conservation versus neo- or sub-functionalization through duplication) [64]–[66].

In this study, we identified the possible non-redundant complete sets of bZIPs in rice, comprising 92 proteins, and in black cottonwood, comprising 89 proteins. These collections of bZIPs together with the 77 bZIPs from Arabidopsis [56] could be divided, based on bZIP domain and other conserved motifs similarities, into 13 groups of bZIP homologues in angiosperms, three more than previously reported [55]. The identified groups constituted a backbone for a more detailed analysis of each group, to which additional bZIP sequences reported from other plants, including those deduced from expressed sequence tags (ESTs), were added. In total, we defined 34 Possible Groups of Orthologues (PoGOs), which may represent 34 ancestral functions in angiosperms. Interestingly, one PoGO was found exclusively in monocots, whereas a Possible Group of Paralogues (PoGP) appears to be restricted to Arabidopsis.

To extend our bZIP analysis to all major lineages of green plants we additionally identified and incorporated bZIP sequences not only from two algal (*Chlamydomonas reinhardtii* [67] and *Ostreococcus tauri* [68]) and moss (*Physcomitrella patens* [69]) genomes, but also from ESTs of the ferns *Selaginella moellendorffii* and *Adiantum capillus-veneris* and the gymnosperms *Pinus taeda* and *Picea glauca*. Based on this investigation, a model for the evolution of *bZIP* genes in green plants, based on four founder genes representing an ancestral tool kit, was established. Its main points are discussed here. We also propose an updated classification of plant *bZIP* genes which should facilitate functional studies.

## Results and Discussion

### Groups of Homologues of Angiosperm *bZIP* Genes

The Arabidopsis genome encodes for a possible complete set of 77 unique bZIP proteins, representing an update of previous results [55], [56], [70]. *AtbZIP73* contains a premature stop codon and was thus not considered further in our analyses. As it appears to be a pseudogene it should be referred to as *ΨAtbZIP73*. Through iterated searches with tblastn and blastx algorithms, and PFAM bZIP Hidden Markov Models (HMM), we identified 92 *bZIP* genes in rice (Text S1a). Recently, Nijhawan *et al.* [71] reported the presence of 89 *bZIP* genes in rice and their phylogenetic relationship to the Arabidopsis *bZIPs*. Of the 89 bZIPs, 86 are also present in this study. Careful sequence analyses of both gene sets revealed complete sequence identity of the Os06g50480 and Os06g50830 TFs, and complete identity with TF Os06g50600 (OsbZIP14) along amino acids 1–143, indicating that these sequences were redundant in the Nijhawan *et al.* data set. *Os03g59460* has also been identified in our studies, however, the protein it encodes contains a proline residue at the beginning of its leucine zipper, precluding dimerization [25]; thus it may not function like other known bZIPs. Despite *OsbZIP24* and *OsbZIP75* being classified as retrotransposons in TIGR, we included them in our analysis as they possess a standard bZIP sequence in their open reading frame. Table S1 gives a summary of this information.

We identified 89 bZIP sequences in *P. trichocarpa*, some of which were incomplete. We therefore performed a more refined analysis of genomic data sets taking into account gene structures and conserved motifs. This allowed us to resolve the entire *bZIP* gene sequences in nine cases (Datasets S1 and S2).

**Figure 1:**
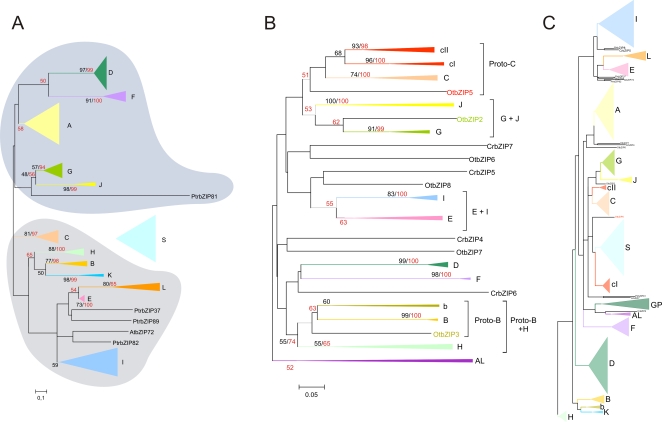
Phylogeny of bZIP transcription factors in green plants. (A) Model of angiosperm bZIP evolution with two large clades, one including groups A, D, F, G and J, and the other including groups B, C, E, H, I and L. Sister groups B and K, E and L, D and F, and G and J, respectively, were defined based on bootstrap support of >50%. The position of Group S could not be clearly defined. (B) Consensus tree inferred from NJ analyses of bryophyte and algal bZIP sequences. This tree reveals new evolutionary relationships among green plant bZIPs, which were not observed when the complete ViridiZIP set was analyzed. Group C appears to be related to two other groups (cI and cII) and members of these three groups are orthologues of *OtbZIP5*, constituting the Group Proto-C. Group b was identified as a sister group of Group B and genes of both groups are orthologous to the algal *OtbZIP3* gene, forming the Group Proto-B. Groups Proto-B and H have a common ancestral origin. Similarly, Groups G and J diverged from the same ancestor and are both orthologous to the algal gene *OtbZIP2*. Finally, Groups E and I show a sisterhood relation but no ancestral link to a bZIP from algae could be established. (C) Tree inferred from NJ analyses of the ViridiZIP data set (bZIPs from algae to angiosperms). This tree indicates that Group S probably originated from Proto-C, and Group K from Proto-B. Tree topology and functional data support these hypotheses. Bootstrap values were calculated from NJ analyses. Red, values obtained with p-distances and, black, with PAM matrix.

Through Neighbour-Joining (NJ) analysis of the minimum bZIP domain (44 amino acids; Text S1a) of 257 unique bZIPs from Arabidopsis, rice and black cottonwood (bZARP data set) we identified seven clusters of proteins with bootstrap support greater than or equal to 50%, defining the groups of homologous genes B, D, F, G, H, J and K. The topology of the phylogenetic tree and a bootstrap support of 50% indicate that Groups D and F are sister groups that share a common ancestor (Figures 1A and S1). Although Group A has a weaker bootstrap support in NJ analyses (34% using PAM matrix data, and 58% using p-distance values), its members were kept together for two main reasons: (i) all its member genes share a common motif in accordance with previous results from Jakoby *et al.* [55]; (ii) all genes but *Gbf4* (*AtbZIP40*) and *AtbZIP13* from Arabidopsis share common intron positions, suggesting a single evolutionary origin (Text S1b, and Figure S2). In Group F a clear tendency for loss of introns was observed. None of the rice *bZIP* genes contains introns, nor do the black cottonwood genes *PtrbZIP39* and *PtrbZIP40*. Although *PtrbZIP38* and *PtrbZIP41* have introns, they lost it from the conserved basic motif. The only gene that possesses an intron in this motif is *AtbZIP24* from Arabidopsis.

**Figure 2:**
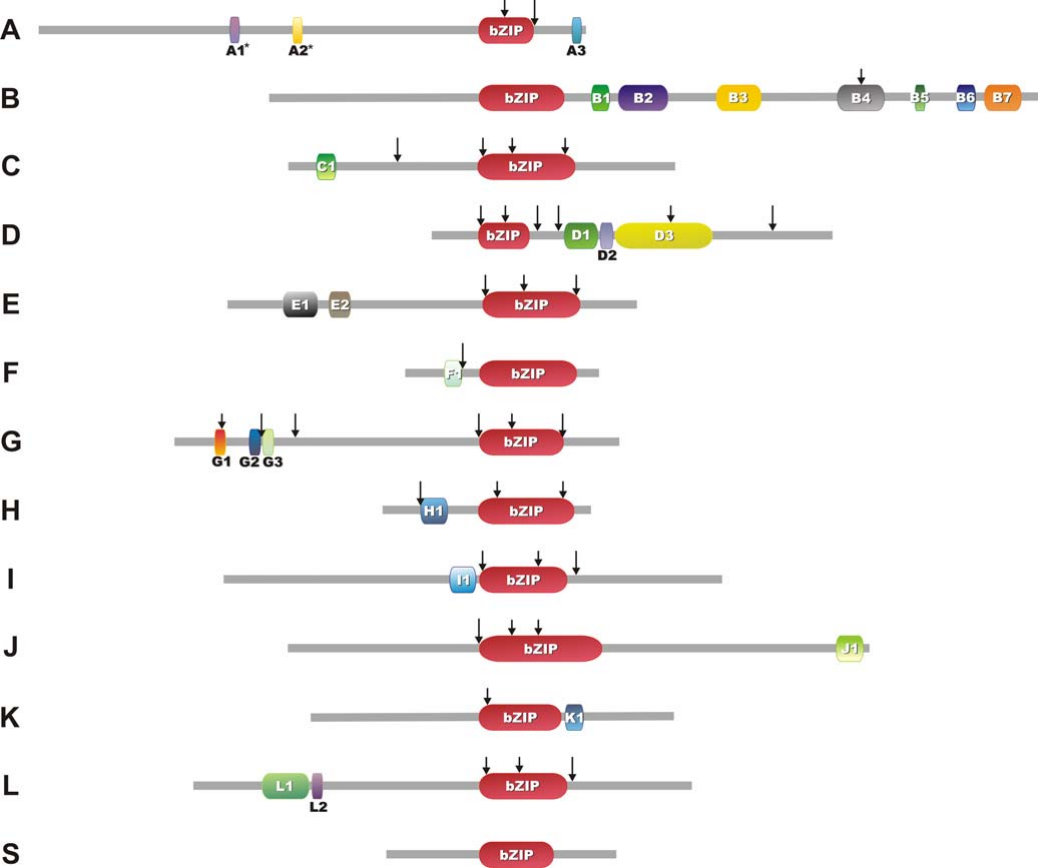
Motifs conserved in angiosperm bZIPs. A summary of the motif sequences is given in Table S2. Arrows indicate intron positions conserved among most members of each group. Representative bZIP sizes and positions of conserved motifs are shown. (*) Group A has two motifs (A1 and A2), that are important putative kinase phosphorylation sites involved in ABA responses. Both motifs appear to be conserved in most members of this group of homologues, except for OsbZIP8, 13, 14 and 15, and PtrbZIP5 and 10, which lack motif A1. The same sequences and also PtrbZIP9 lack motif A2. Due to the lack of complete sequences, no structures are shown for Groups AL, GP, b, cI and cII.

Members of Groups A and D have a bZIP domain of only 44 amino acids. To refine our analysis we created a subset-of-bZARP (sbZARP) dataset that excluded groups A and D members but included all remaining 172 proteins with a bZIP domain of 60 amino acids (53, 60 and 59 bZIPs from Arabidopsis, rice and black cottonwood, respectively). NJ analyses revealed four new groups of homologues, Groups C, E, I and L, all supported by bootstrap values of >50% (Figure S3; note that Group L members harbor an atypical basic motif; see Figure S2, and Text S1c). The overall organization into twelve groups is further supported by the presence of at least one shared intron position among the members of each group, confirming a common ancestral origin of all its members (Figures 1A, 2 and S2). The twelve groups encompass 199 of the 257 bZIPs of the bZARP data set. Fifty-three of the remaining bZIPs (17, 17 and 19 from Arabidopsis, rice, and black cottonwood, respectively) tended to form a separate group, defined as Group S in agreement with previous data [55]. However, this group did not have significant bootstrap support. Members of Group S bZIPs share two characteristics: they harbor a long leucine zipper (eight to nine heptads) and are encoded by intron-less genes. Finally, *AtbZIP72* (Arabidopsis) and *PtrbZIP37*, *81*, *82* and *89* (black cottonwood) could not be classified into any of the above groups (Figure 1A).

**Figure 3:**
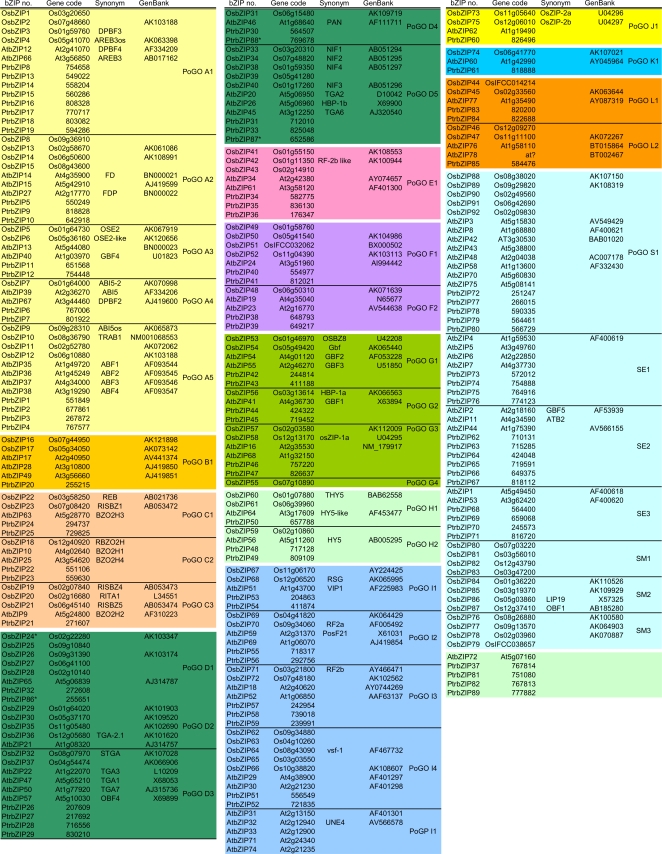
Classification of bZIPs from Arabidopsis, black cottonwood and rice. Thirteen groups of homologues (A to L, and S) were defined through NJ phylogenetic analyses with the bZARP set (Figures S1 and S3). The organization into Possible Groups of Orthologues (PoGOs) was done by more refined NJ phylogenetic analyses inside each group of homologues, including also sequences from other eudicots and monocots. The alignment used for these analyses corresponds to a concatenated sequence of the group-specific conserved motifs identified employing MEME (http://meme.sdsc.edu/meme/website/intro.html; Figure 2). (*) Represents genes that lack group-wise conserved motifs, thus they were included inside a PoGO according to their best hit to another bZIP. Because the relation of AtbZIP72, PtrbZIP37, 81, 82 and 89 could not be clarified, they were not included in any of the groups of homologous or orthologous genes. One Possible Group of Paralogues (PoGP I1) was found in Arabidopsis. Column ‘Gene code’ provides the gene identifiers for Arabidopsis, black cottonwood and rice bZIP sequences taken from TAIR (http://www.arabidopsis.org/), JGI (http://www.jgi.doe.gov/) or TIGR (http://www.tigr.org/), respectively. ‘Synonym’ indicates published and often cites names of bZIP genes. The GenBank accession numbers of nucleotide sequences are given.

In summary, our data suggest 13 groups of homologous angiosperm *bZIP* genes (A, B, C, D, E, F, G, H, I, J, K, L, and S), representing a unified classification of angiosperm bZIPs (Figure 3) [55], [56], [71]. This result is in agreement with previous analyses, but additionally revealed three new groups (J, K and L) (Figure S3). The name of each group of homologues follows the classification established by Jakoby *et al.* [55]. Similar conclusions were reached using Maximum Likelihood analyses.

### Possible Groups of Orthologues (PoGOs) in Angiosperms

We next aimed at identifying Possible Groups of Orthologues (PoGOs) among the 13 groups of homologues. By definition, each PoGO represents a group of genes that diverged from an ancestral gene through speciation and duplication. Members of a given PoGO typically have closely related biological functions, and this allows making predictions for poorly characterized genes and rationalizes functional studies of the proteins they encode [72]. PoGOs also establish a basis for the definition of functional diversification among genes. Here, we identified PoGOs by NJ analysis of each group of homologues separately, using the criteria defined in Material and Methods. To optimize the resolution of the evolutionary relationships, alignment lengths were extended by including conserved motifs specific to each group of homologues (Figure 2, and Table S2). Additionally, 636 further bZIP sequences, 260 from eudicots and 376 from monocots (Table S3), were extracted from EST databases. These new bZIPs were included in the respective groups of homologous genes according to their tblastn best matches against members of an upgraded Angiotot dataset that contained the rice and black cottonwood bZIPs.

Our analysis revealed 31 PoGOs distributed among Groups A to L (Figures 3 and S4, S5, S6, S7, S8, S9, S10, S11, S12, S13, S14 and S15). In all PoGOs except D2, at least one black cottonwood bZIP sequence could be included (Figure 3) further supporting the organization into PoGOs. The lack of a black cottonwood *bZIP* gene in PoGO D2 could be due to an absence of such a gene in its genome or to incomplete genome sequence availability. OsbZIP24, PtrbZIP86, 87 and 88 lack some of the motifs conserved in Group D members and were therefore assigned to the PoGO to which they showed the highest overall sequence similarity (as identified through blastp analysis).

We identified only one eudicot-monocot PoGO, S1, in Group S (Figure S16). The remaining sequences could be clustered into three PoGOs each restricted to either eudicots (SE1, SE2 and SE3) or monocots (SM1, SM2 and SM3) (Figure S16). Arabidopsis bZIP TFs of groups SE2 and SE3 are involved in energy metabolism and hypoosmolarity signaling (Table S4) further supporting the evolutionary relationship deduced from the phylogenetic analysis. Similarly, SM2 members play a role in cold signaling (Table S4), thus providing function-based support also for this group. Although further efforts to more precisely uncover the relationship between the three monocot (SM1, SM2 and SM3) and eudicot (SE1, SE2 and SE3) groups of orthologues proved unsuccessful, we propose that up to three additional eudicot-monocot PoGOs, besides S1, exist in Group S (as a minimal representation of the three possible monocot and eudicot PoGOs). The difficulty of organizing Group S bZIPs into PoGOs that comprise both eudicots and monocots sequences may reflect an increased evolutionary rate after their emergence. Rapid evolution can mainly be explained by relaxation of purifying selection or by positive selection. We used the Yang algorithm [73] to verify whether lineage-specific dN/dS ratios in Arabidopsis, black cottonwood and rice (the ω parameter, [74], [75]) of Group S were different from that of all other groups. The ω value for Group S (0.12) was found to be significantly different from the average ω calculated for all other groups (0.03, likelihood ratio test 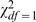, *p*<0.01). Despite being under purifying selection (ω<1), the value of ω for Group S is four times higher than the average. Thus it can be concluded that purifying selection is relaxed in this group, explaining the higher rate of sequence divergence among its members. Low selective constraint (i.e., low purifying selection) is a hallmark of more recently duplicated genes and can be correlated with functional diversification [76]. The extensive amplification of Group S members in angiosperms (see below) further supports the notion that functional diversification partly related to the control of energy metabolism is operating among Group S genes.

In Group G, we observed one PoGO that is restricted to monocots (PoGO G4; Figure S10). This may be explained by gene gain at an early phase of monocot radiation, or alternatively by gene loss in the ancestor of the eudicot lineage. Our analysis also revealed the existence of a Possible Group of Paralogues (PoGP) restricted to Group I in Arabidopsis (PoGP I1, Figure S12). This PoGP most probably reflects a recent duplication event followed by rapid divergence in the Arabidopsis lineage. As PoGO G4 and PoGP I1 are restricted to distinct evolutionary lineages, they probably do not play essential (common) roles in angiosperms as a whole. This conclusion is supported by the fact that EmBP from maize and wheat, both assigned to PoGO G4, control reserve protein (prolamin) production [77] which can be considered a monocot-specific function.

Gene duplication is an important means of evolutionary diversification. Therefore, PoGOs that preferentially expanded during angiosperm evolution are expected to include genes that were particularly important for establishing angiosperm-specific physiological or functional characteristics. Of the 13 groups of homologous genes, Groups A, D, E, I and S contain more genes per PoGO than the average (approximately six genes per PoGO, Figure S17), indicating their preferential contribution to the evolution of adaptive characteristics in angiosperms. Interestingly, Groups A, D and S include genes for responses and adaptation to environmental factors (abiotic and biotic stresses in Groups A/S and D, respectively; Table S4) and the control of energy use (Group S; Table S4). These observations raise the possibility that genes of these groups were particularly important for the colonization of new habitats and consequently for the radiation and expansion of angiosperms (Text S1d). Additionally, some PoGOs have a conserved one-to-one gene relationship, indicating that their genes may play a pivotal role during development (Text S1e)

In summary, we propose the existence of 31 monocot-eudicot PoGOs in Groups A to L, one monocot-specific PoGO (G5), one PoGP (I1) in Arabidopsis, and possibly three PoGOs in Group S. The 34 PoGOs are likely to be related to 34 possible ancestral functions of bZIPs in angiosperms (Figure 3, and Text S1d).

### Tracing the Origin and Diversification of *bZIP* Genes in Green Plants

Based on the phylogenetic analyses and the *bZIP* gene structures from Arabidopsis, black cottonwood and rice, we propose a model for the evolution of angiosperm bZIPs (Figure 1A). This model proposes two large clades encompassing Groups A, D, F, G and J, and Groups B, C, E, H, I, K and L, respectively. Groups B, H and K, Groups E and L, and Groups D and F are sister groups, as evidenced by their bootstrap support. Furthermore, the conserved intron position in the bZIP domain shared by Groups A, D, G and J, as well as the one shared by Groups C, E, H, I, K and L (Figure S3) supports the hypothesis that these groups diverged from a common ancestor. We were not able to establish a clear relationship of Group S to any of the two larger groups. It may have an independent ancestral origin, constituting a third group, or may have evolved from one of the two large groups (Figure 1A).

To identify groups of homologues among the major eukaryotic lineages, i.e. animals, fungi, and plants, we performed a large-scale phylogenetic analysis using the conserved bZIP region of all bZIPs from *Homo sapiens* [78], *Caenorhabditis elegans* (http://www.wormbase.org/), *Drosophila melanogaster* [79], *Saccharomyces cerevisiae* (http://mips.gsf.de/genre/proj/yeast/), *A. thaliana* and *O. sativa*. This analysis revealed that *bZIPs* of each of these lineages share only one common ancestor (data not shown) which is in accordance with the fact that only a single *bZIP* sequence is present in the primitive eukaryote *Giardia lamblia* [80], [81], perhaps representing the *bZIP* gene content prior to the plant/animal/fungal separation [80]. The function of this unique ancestral gene may be related to unfolded protein (UPR) and oxidative stress responses (see below). Deep evolutionary analyses have also been performed for the homeodomain and MADS-box families and it appears that their member TFs derived from at least two genes present in the last common ancestor of the three eukaryotic kingdoms [19], [82]. It has been proposed that one of the ancestral functions of the MIKC^c^ class of MADS-box genes is an involvement in reproductive organ development [83], [84]. Although this function appears to be conserved, it is still not clear whether it has a monophyletic origin.

**Figure 4:**
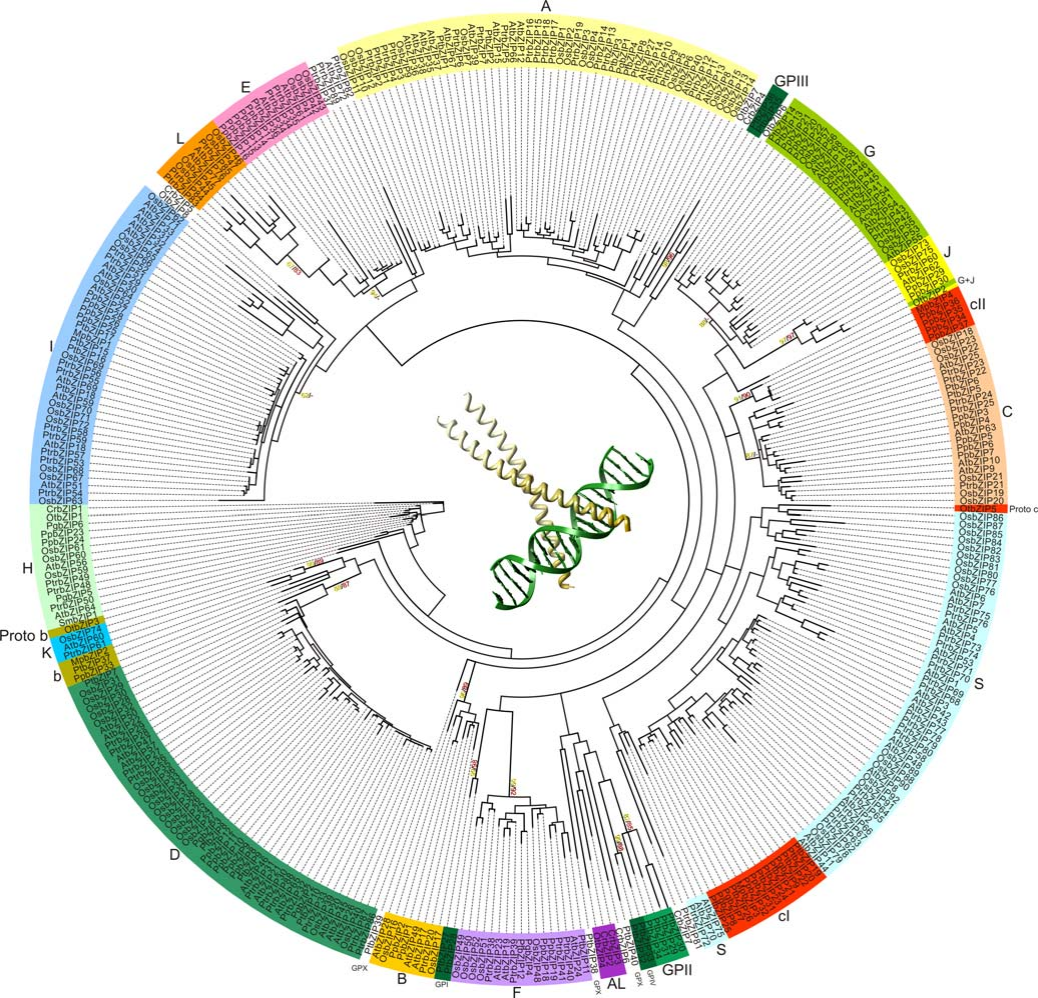
Global Phylogeny of bZIPs in green plants. This tree is a consensus of NJ analyses with p-distance performed with the ViridiZIP set. Bootstrap values in yellow were calculated from NJ analysis (PAM matrices, and with 44 and 60 amino acid alignments; only the highest bootstrap values are shown). Bootstrap values in red were calculated from ML analyses using the JTT+Γ evolutionary model (either with 44 or 60 amino acid alignments; only the highest bootstrap values are shown). GPX, GPI, GPII, GPIII, and GPIV indicate putative gymnosperm specific groups. Each group of homologues is colored following the same colour scheme used in Tables I and SV. The center of the tree depicts a typical bZIP dimer bound to DNA, representing the conserved bZIP domain (GCN4 from *Saccharomyces cerevisiae*; Protein Data Bank entry 2DGC).

We identified 7, 8, and 40 *bZIP* genes, respectively, in the genomes of the algae *Chlamydomonas reinhardtii* and *Ostreococcus tauri* and the moss *Physcomitrella patens* (however, a complete bZIP domain is missing in three of the moss proteins). Additionally, we identified bZIP sequences from assembled ESTs of species representing the most relevant divisions of the green plants from which sequences are available: four *bZIP* genes in the bryophyte *Marchantia polymorpha*, one each in the ferns *Selaginella moellendorffii* and *Adiantum capillus-veneris*, and 40 and nine, respectively, in the gymnosperms *Pinus taeda* and *Picea glauca* (Table S5). Although no complete genomic sequences were available for ferns or gymnosperms, a considerable number of ESTs is available for the latter. We assembled a set of 345 bZIPs from algae to angiosperms (ViridiZIP set) for phylogenetic analyses (Figures 1B, 1C and 4).

Our study revealed that Group H is the most conserved group of *bZIP* homologues; members of this group are present in all green plant lineages. This observation is particularly interesting because Group H includes *HY5* and *HYH* that are important regulators of light responses and anthocyanin biosynthesis (Table S4). We therefore propose that Hy5-like bZIPs control light-dependent processes in all green plants. Similar to bZIPs in Group H, DOF transcription factors involved in light responses (subfamily A) also appear to be well conserved, suggesting that genes involved in light-related functions are under strong selective constraints [85]. In Arabidopsis Hy5-mediated photomorphogenesis is negatively regulated by the E3 ubiquitin ligase Cop1, which ubiquitylates Hy5 protein leading to its degradation [86]. We detected Cop1-related proteins in Physcomitrella, in agreement with previous results, as well as the Cop1-interaction motif in Physcomitrella Hy5-like bZIPs, suggesting that the genetic toolkit for photomorphogenesis described in angiosperms is also present in mosses [87]. We also detected a single gene similar to *COP1* in Ostreococcus (ID 30007), but while in higher plants Cop1 protein contains a RING domain at the N-terminus, followed by multiple WD40 repetitions [88], this order is reversed in the Ostreococcus protein. Moreover, a Cop1 interaction site (Table S2) was not detected in the algal *HY5*-orthologues OtbZIP1 or CrbZIP1, or in any other green algae bZIP. Nevertheless, we found one Cop1-related protein in the red alga *Cyanidioschyzon merolae* (ID CMK039C; http://merolae.biol.s.u-tokyo.ac.jp/). Cop1-like proteins are also known in animals where they promote the degradation of the bZIP transcription factor c-Jun [88], suggesting Cop1-dependent protein degradation to be a regulatory scheme conserved in most eukaryotes.

**Figure 5:**
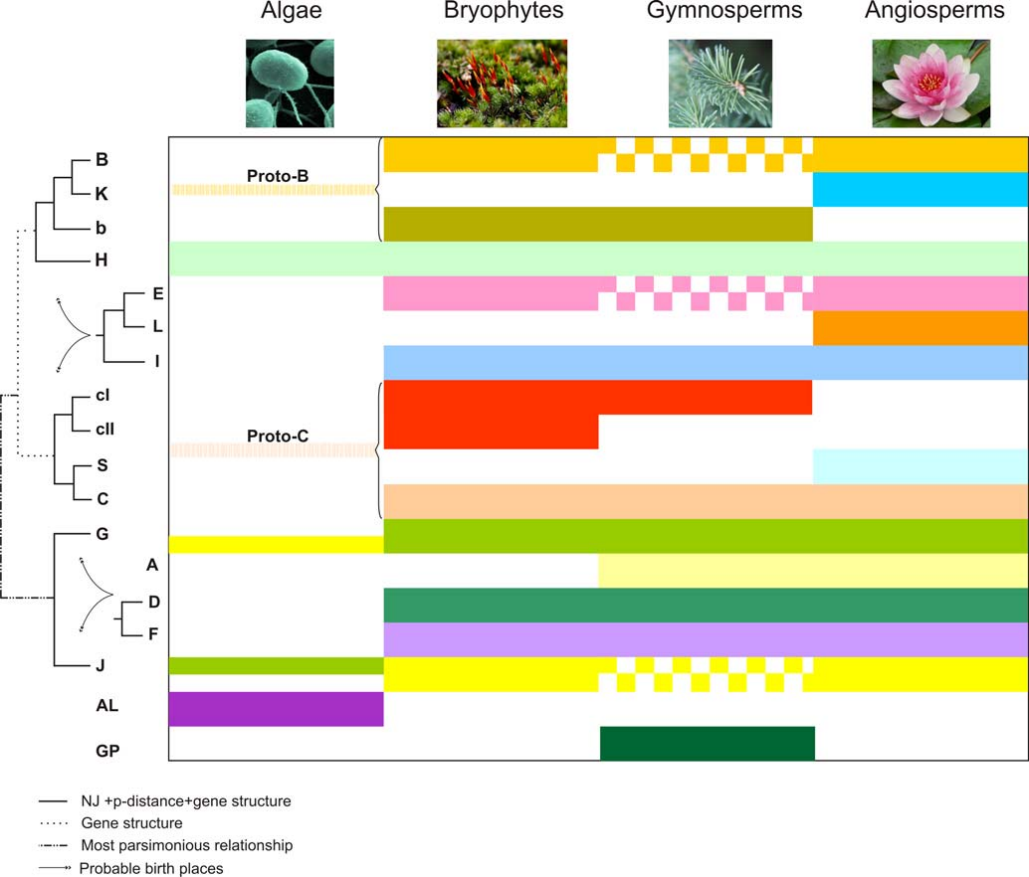
Phylogenetic profile and structure of bZIPs in green plants. Groups E, L and I belong to the same branch as Groups Proto-B, Proto-C and H but their exact position is not clear (Figure 1A). Similarly, Groups A, D and F do not have a clear position, though they belong to the same branch as Groups G and J (Figure 1A). The relation of Groups AL and GP to the other groups could not be established. bZIPs of the species studied here were grouped at the level of higher taxa, i.e., algae (represented by *C. reinhardtii* and *O. tauri*); bryophytes (*P. patens*); gymnosperms (*P. glauca* and *P. taeda*), and angiosperms (*O. sativa*, *A. thaliana* and *P. trichocarpa*). Solid boxes indicate that at least one bZIP was found for a given group of homologues in the respective taxon. Squared boxes indicate that homologous bZIP sequences were not yet observed in gymnosperms, possibly due to sampling limitations. Notably, however, sequences of the respective groups are conserved in bryophytes and angiosperms. Dashed lines with brackets shown in Groups Proto-B and Proto-C indicate that there is an orthologous bZIP in at least one of the algal species, although it does not strictly belong to any of the homologous groups. The half lines present in G and J indicate the presence of common orthologues in algae. Groups AL, GP, K, L and S appear to be lineage specific.

Groups B, C, D, E, F, G, I and J were present in the most recent common ancestor (MRCA) of bryophytes and tracheophytes, indicating a functional connection to the colonization of the terrestrial environment (Figure 5). Some of these genes play a role in light responses (Group G), nitrogen/carbon balance control (Groups C and G), and ion responses (Group D), which are some of the important features that developed further in embryophytes (Table S4). Moreover, it appears that during the evolution from early land plants to angiosperms, Group D and I genes amplified more than genes of the other groups of homologues (5 to 10, and 4 to 11 genes in groups D and I, respectively), strongly suggesting that both groups were particularly important for this transition. Several Group D genes are involved in biotic stress responses (Table S4) indicating that improved pathogen defense was important for land plant evolution. Some *bZIP* genes of Group I control the expression of vascular genes (Table S4), which are central to vascular tissue development in tracheophytes.

Group A probably first appeared in the MRCA of spermatophytes and may thus be related to seed formation (Figure 5). As a matter of fact, Group A bZIPs often have functions in seed development, ABA responsiveness and fruit maturation (Table S4). Moreover, they are elements of ABA-dependent signaling pathways that coordinate responses to desiccation/dehydration and salt stress. ABA-mediated signaling is known in Physcomitrella [89], [90], however, Group A bZIPs are not present in this organism (Figure 5), indicating a less developed ABA regulatory network (Text S1f).

According to our data Groups K, L and S are angiosperm-specific (Figure 5). However, due to sampling limitations we can not formally exclude the possibility that these groups are also present in gymnosperms. Additionally, this analysis eliminates the hypothesis that Group S has an independent ancestral origin (Figures 1A and 1C).

We also detected Group NA, a possible group of homologues exclusively present in non-angiosperm plants (Figure S18, and Text S1g). This finding is intriguing as genes conserved in mosses and gymnosperms are expected to represent general plant functions. Group NA bZIPs may thus have lineage-specific roles unimportant for angiosperms; the reduction of a dominant gametophyte during angiosperm evolution combined with a concomitant gene loss is an example for this. Alternatively, gene loss could have played a key role in the acquisition of important features in angiosperms, as seen for *KNOX* genes [91]; or, the roles played by bZIPs of Group NA could have been taken over by non-related but functionally analogous genes (non-orthologous gene displacement).

### Ancestral Relationships in Groups B and C

The above analysis in combination with detailed sequential NJ analyses restricted to algal, moss and/or Arabidopsis sequences revealed two new groups, i.e. Groups Proto-B and Proto-C (Figure 1B). Group Proto-C encompasses Group C (Figure 1A) and two new Groups, cI and cII that correspond to the sequences previously identified in Group NA (Figure S18). While cI appears to be restricted to bryophytes, cII is found up to gymnosperms, and C is present up to angiosperms (Figures 1C and 5). Notably, in all phylogenetic analyses Group S appeared to be more attracted by Groups C, cI and cII (Figures 1C, 4 and 5), suggesting it originated from Group Proto-C, probably by gene duplication followed by rapid evolution. This finding is supported by the observation that bZIPs tend to dimerize with more similar partners, e.g. AtbZIP10 (Group C) with AtbZIP53 (Group S) [34], [92]. Additionally, members of Group C (*AtbZIP63*) and S (*ATB2*, *GBF5*, *AtbZIP1* and *AtbZIP53*) participate in the control of energy metabolism and thus share similar functions (Table S4). Moreover, Group Proto-C possesses one *bZIP* gene, *OtbZIP5* from Ostreococcus, supporting the model that the biological functions played by bZIPs of Group C/S, such as oxidative stress responses associated with *AtbZIP10* [40] and energy metabolism control mediated for example by *GBF5* [41], are at least partially present in all green plants. Importantly, oxidative stress signaling involving bZIPs has been reported in yeast and men and thus appears to be conserved in all eukaryotes [93]–[97].

Group Proto-B consists of Group B, which includes members from bryophytes and angiosperms, a new group of homologues (Group b) that is apparently restricted to bryophytes and gymnosperms, and the Ostreococcus gene *OtbZIP3* (Figures 1B, 4 and 5). Based on our initial phylogenetic analysis of angiosperm sequences (Figure 1A) and tree topology (Figures 1C and 4) we concluded that angiosperm-specific Group K is not only a sister group of B, but very likely also emerged from Proto-B. Members of Group K are likely to have a role in the unfolded protein response (UPR), a cellular process involving the endoplasmic reticulum (ER) that counteracts cellular stress when incorrectly folded proteins accumulate [43]. bZIPs involved in this response are known in mammals and yeast and thus appear to be conserved in many lineages [98], [99]. Recently, Liu et al. [42] demonstrated a role of Arabidopsis AtbZIP17 (Group B) in the UPR pathway, supporting the hypothesis that Group K emerged from Group B, and that *OtbZIP3* plays a similar role. Members of Groups B and K (like animal bZIP proteins involved in UPR) posses a trans-membrane domain for ER attachment (Table S2), but members of Group K lack the cleavage site recognized by the so-called site-1 protease (S1P). Most likely, the two groups function in different branches of the UPR pathway. Additionally, we looked for the presence of both trans-membrane and S1P interaction domains in other plant proteins. The trans-membrane domain is present in all Group B and K bZIPs from green plant lineages, whereas the S1P interaction domain was not found in some of them, perhaps due to missing sequence data.

Another important result of our analysis is that Ostreococcus sequences could be included, with significant bootstrap support, into Groups Proto-C (*OtbZIP5*) and Proto-B (*OtbZIP3*; Figure 1B). Moreover, Ostreococcus *OtbZIP2* was found to significantly cluster with Groups G and J, forming a new group named G+J (Figure 1B).

**Figure 6:**
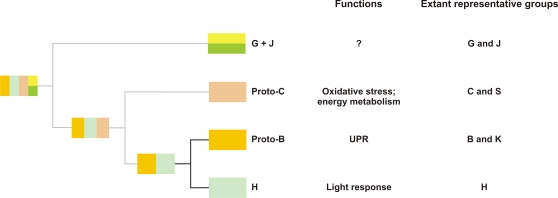
Most parsimonious model explaining the emergence of the four green plant founder *bZIP* genes. The four founder genes (in Groups G+J, Proto-C, Proto-B and H) are derived from a unique ancestral gene common to all eukaryotes. Groups Proto-B and Proto-C most likely derived from a multifunctional UPR/oxidative stress gene. Groups Proto-B and H are sister groups and their relationship to Group Proto-C was found by analyzing angiosperm bZIPs (Figure 1A). Group G+J is the ancestral group of a large set of *bZIP* genes included in Groups A, D and F, but the ancestral function played by this group is still largely unknown.

In conclusion, our results indicate that four Ostreococcus *bZIP* genes can be assigned to Groups Proto-C (*OtbZIP5*), Proto-B (*OtbZIP3*), G+J (*OtbZIP2*), and H (*OtbZIP1*), defining four orthologous relationships between algal and five groups of homologues from terrestrial plants (Figure 6). This data suggests the presence of at least four founder genes in the MRCA of green plants. Our analysis also indicates that Groups H (including *OtbZIP1* and *CrbZIP1*) and Proto-B (including *OtbZIP3*) originated from a common ancestral gene (Figure 1B). However, their relationship with Proto-C (*OtbZIP5*) and G+J (*OtbZIP2*), and the relationship of the four founder genes to the possible monophyletic origin of bZIPs in green plants could not be determined. The most parsimonious model that can explain the origin of the four ancestral bZIPs is shown in Figure 6. The assumption that Group Proto-C and Groups H/Proto-B share a common ancestral gene was inferred from the observation that angiosperm Groups C, B and H also cluster together (Figure 1A). Similarly, all DOF TFs appear to have originated from a single founder gene from subfamily A, which was present in the MRCA of green plants and might have played a role in light-regulated mechanisms [18]. In addition, MADS-box TYPE II (MIKC^c^) and HD-Zip class III TF families each emerged from a single founder gene present in the MRCA of streptophytes that was possibly involved in haploid reproductive cell differentiation [84] or control of apical growth [23], [24], respectively.

### bZIP Evolution in Plants

Our data show that Group C and B members are elements of the oxidative stress signaling and UPR pathways, respectively, which appear to be crucial in all eukaryotes. This observation and the likely monophyletic origin of bZIPs of the main eukaryotic lineages (plants, animals, and fungi) suggest that the common bZIP ancestor was a multifunctional regulatory factor. An important consequence of this model is that Group H, which has a central role in light-mediated control, emerged from bZIPs of the oxidative stress and UPR regulatory modules. The integration of the branch leading to Group G+J, however, remains unclear which is partially due to the fact that functional information is limited and restricted to Group G that plays a role in light and ABA signaling.

From the extant algal sequences that do not cluster into any of the homologous groups of streptophytes, only a single group of homologues restricted to algae could be detected (Group AL; Figures 1C and 5). In most cases bZIP sequences from Chlamydomonas and Ostreococcus do not cluster together at all. This observation indicates that bZIPs evolved differently in the algal lineages, probably reflecting adaptations to different ecological niches; Chlamydomonas lives in fresh water, while Ostreococcus lives in sea water.

We estimated the number of bZIPs in the MRCA of all land plants (embryophytes), using the method of Hahn *et al.* [100]; the MRCA most likely had 64 bZIPs that expanded to 83 in the branch leading to seed plants. The rate of gene gain-loss, λ, in the seed plant lineage was found to be 2.01×10^−3^ per million years, which is similar to estimates for yeast (0.002) [100] and mammals (0.0016) [101]. We calculated expansions and contractions of the bZIP phylogenetic branches in the land plant lineage, using the estimated value for λ; this revealed a significant expansion (*p*<0.05) of the branch leading to the seed plant lineage. Finally, the evolution of the *bZIP* gene family is well explained by the random birth-and-death model in seed plants, i.e., no significant expansions/contractions occurred preferentially in any specific PoGO or group of homologues (Figure S19, and Text S1h).

### Conclusions

In our analysis presented here we systematically classified bZIP TFs into PoGOs and considered existing knowledge about their biological functions to establish a robust methodology to reveal evolutionary relationships of this group of regulatory proteins. The moss Physcomitrella possesses almost five times more *bZIP* genes (37 genes, Table S5) than the alga Ostreococcus (8 genes), and half the number found in angiosperms (around 80 genes). Group A genes first appeared in the MRCA of spermatophytes and were recruited for seed development or germination but also to fine tune the responses to desiccation/dehydration and salt stress. Groups K, L and S are seemingly exclusive to angiosperms. Unexpectedly, Groups K and S control processes conserved in all eukaryotes, i.e. UPR and energy homeostasis. This apparent paradox can be explained by the fact that both, Groups K and S derived from the functionally related Groups Proto-B and Proto-C, respectively, that emerged early on during green plant evolution. Group S amplification likely contributed to refining the regulatory circuit controlling the organism's energy status. The most strongly conserved group of homologues in algae and angiosperms is Group H which includes light control factors *HY5* and *HYH*. Group H is representative of one of the four green plant founder *bZIP* genes. Our data thus establish the hypothesis that bZIP-controlled light responses of Group H emerged (through neofunctionalization) from a multifunctional ancestral gene of the UPR and oxidative stress response pathways (UPR/oxidative stress). The UPR/oxidative stress gene is also the ancestor of two other of the four founder genes, i.e. Groups Proto-B (UPR) and Proto-C (oxidative stress), which most likely diverged through subfunctionalization processes. The fourth founder gene, represented by Groups G and J, is the sister gene of the multifunctional UPR/oxidative stress gene. More functional data for Group G- and J-related bZIPs are required to further elaborate the model of green plant bZIP evolution.

## Materials and Methods

### Datasets of *bZIP* Genes

We generated a bZIP dataset (Angiotot) representing an updated version of the ABZ data set [56]. Plant bZIP sequences were identified as described by Riaño-Pachón et al. [102]. The whole proteomes deduced from the completely sequenced genomes of the algae *Ostreococcus tauri* [68] and *Chlamydomonas reinhardtii* [67], the bryophyte *Physcomitrella patens* [69], and the angiosperm *Populus trichocarpa* [59] were downloaded from the Joint Genome Institute/Department of Energy (JGI/DOE; http://www.jgi.doe.gov/). Protein sequences for the angiosperm *Arabidopsis thaliana* [54] were downloaded from The Arabidopsis Information Resource (TAIR; http://www.arabidopsis.org/), and from The J. Craig Venter Institute (http://www.tigr.org/) for the monocot *Oryza sativa* ssp. *japonica* [58].

Assembled ESTs from *Marchantia polymorpha*, *Physcomitrella patens*, *Adiantum capillus-veneris*, *Selaginella moellendorffii*, *Picea glauca*, *Pinus taeda*, *Brassica napus*, *Glycine max*, *Heliathus annus*, *Medicago truncatula*, *Solanum lycopersicum*, *Solanum tuberosum*, *Hordeum vulgare*, *Saccharum ssp.*, *Sorghum bicolor*, *Triticum aestivum* and *Zea mays* were downloaded from the TIGR Plant Transcript Assemblies Database [103]. ESTs from *Oryza sativa* ssp. *indica* were downloaded from the Beijing Genomics Institute website (07.11.2006), and assembled into clusters using TGICL [104]. Additional rice bZIP sequences were obtained from the Full Length Rice cDNA Consortium [105]. Some sequences from completely sequenced genomes were re-annotated (Datasets S1 and S2), based on conserved protein motifs and gene structures of each family. The list of abbreviations of the organisms used is given in Table S6.

The tblastn program [106] was used to search for bZIP sequences in rice nucleotide databases (*Oryza sativa* ssp. *indica* [57]; Beijing Genomics Institute, http://btn.genomics.org.cn/rice, and *Oryza sativa* ssp. *japonica*; Syngenta, http://www.syngenta.com/; IRGSP, http://www.gramene.org/) using Angiotot as query. Sequences with an e-value <10^−4^ were selected to form a subset (SeqZIP), from which false positive hits, corresponding mainly to low complexity regions, and hits that we initially identified using the above procedure were excluded. To identify the open reading frame and gene structure of each SeqZIP sequence, pairwise blastx analyses against their respective Angiotot best hits were performed. Gene structures were defined based on the alignments obtained, the conserved positions of introns in homologous bZIP genes, and the presence of canonical splicing sites (GT-AG). The protocol used for bZIP identification is described in Figure S20.

The procedure used to identify bZIPs in EST datasets was identical to that used for genomic sequences, except that the estwisedb program of the Wise2 package [107] was included to identify the most likely reading frames and its bZIP domains in a given cluster.

### Phylogenetic Analyses

Alignment of bZIP protein sequences was performed by ClustalX [108], using default parameters, and subsequently adjusted manually. The alignments used for the analyses within each group of homologues represent a concatenated sequence of the different conserved motifs found within each group (Figure 2). The phylogenetic analyses based on amino acid sequences were conducted using MEGA v3.1 [109] and PHYLIP v3.6 [110]. Unrooted phylogenetic tree topologies were reconstructed by Neighbor-Joining (NJ), the distances were obtained using a PAM-like distance matrix [111], or alternatively, using p-distances [112], and the re-sampling of the original bZIP set was a 1,000 bootstrap repetition. Maximum Likelihood (ML) analyses of the bZIP domain (44 and 60 amino acids) were carried out using RAxML [113] with the distances computed using the JTT+Γ evolutionary model [114], and a re-sampling of the original bZIP set of 500 bootstrap repetitions. Bayesian approaches were not employed as they often lead to very liberal estimates of branch confidence that can result in wrong topologies [115]. Additionally, phylogenetic trees for nucleotide sequences, corresponding to the conserved motifs used for proteins, were inferred by means of the maximum likelihood method available in PAUP 4b10 [116]. The TrN+Γ [117] model of sequence evolution was used. Model choice was performed in MODELTEST 3.6 [118] by the likelihood ratio test with significance level set at 1%. ML trees are available upon request. Branch lengths of the tree comprising all species analyzed were estimated by Maximum Likelihood in TREE-PUZZLE v5.2 [119], using the consensus topology inferred by NJ analysis with PAM-like distances. All sequences and alignments used in this study are available upon request.

### Identification of Conserved Motifs

The putative complete sets of unique bZIPs from Chlamydomonas, Ostreococcus, Physcomitrella, black cottonwood, Arabidopsis and rice served as input for a conserved motif analysis performed with MEME (http://meme.sdsc.edu/meme/meme.html) [120]. Whole protein sequences were employed for this search. A given motif was allowed to appear at any number of repetitions, the maximum width of a motif was set to 80, and the maximum number of motifs was set to 20. The other parameters were used as default. In a complementary approach, each group of homologues was analyzed individually with the parameters described above.

### Phylogenetic Analyses and Identification of Possible Groups of Orthologoues (PoGOs)

The detailed evolutionary analysis of angiosperm bZIP sequence relationships within each group allowed the identification of PoGOs. A PoGO is defined by the following criteria: (i) members of a PoGO have a monophyletic origin, indicated by a bootstrap support greater than 50%; (ii) a PoGO possesses at least one representative gene each from *A. thaliana* and *O. sativa*, assuming that the putative complete sets of *bZIP* genes of these organisms were identified and no selective gene loss had occurred. In case a PoGO is found to be restricted to either monocots or eudicots, the presence of sequences from at least one other species of the same lineage in this PoGO is required; and (iii) the inferred phylogeny should be consistent with the known phylogeny of plant species [56].

### Identification of Pseudogenes and Genomic Duplications

Search for pseudogenes in Chlamydomonas, Ostreococcus, black cottonwood, Arabidopsis and rice was performed by masking the genomic region for each identified bZIP. Blastx searches were performed against the masked sequences using the Angiotot bZIP database as query. A hit was considered as a pseudogene only if it possessed all or part of the bZIP domain; therefore all hits were compared against bZIP PFAM models [121] and manually cured, eliminating false positives. Genomic duplications in Arabidopsis were identified via “Paralogons in Arabidopsis thaliana” (http://wolfe.gen.tcd.ie/athal/dup) and ‘MATDB: Segmental Duplications’ from MIPS (Munich Information Center for Protein Sequences; http://www.mips.gsf.de/projects/plants) (Table S7).

### Analysis of Gene Family Expansion and Contraction

The evolution of rates of *bZIP* gene gain and loss along the history of green plants was analyzed by the method of Hahn *et al.* [100], implemented in CAFÉ [122]. The method models gene family evolution as a stochastic birth-and-death process implemented as a probabilistic graphical model that allows for the inference of the most likely family sizes in the common ancestors of every branching point. In this way one can test the null hypothesis of random change in the family size. To avoid incomplete sampling, only plants with fully sequenced genomes were analyzed. The algorithm developed by Hahn *et al.* uses a birth-and-death parameter, λ, which was also estimated within CAFE. In addition to the parameter λ, CAFE needs divergence times to be entered along with the phylogeny of the organisms used. Since the inference of the size of gene families at deep evolutionary times is not reliable with any of the current methods available (Hahn, personal communication; [100]), we focused on land plants only. Tree topology and divergence times are shown in Figure S19. Significance of the contractions and expansions along branches was accessed by means of the three methodologies available in CAFE: branch cutting, likelihood ratio test, and Viterbi assignments [122].

### Gene Expression Analysis

Absolute signal intensity values from Arabidopsis ATH1_22K array (Affymetrix) was obtained through Meta-Analyzer from GENEVESTIGATOR (http://www.genevestigator.ethz.ch/) [123]. The developmental stages were as described by Boyes *et al.* [124]. Massively Parallel Signature Sequencing, MPSS, [125] was also verified for Arabidopsis and rice genes (Datasets S3 and S4).

## Supporting Information

Figure S1 Definition of homologous gene groups A, D and F. This figure is a partial representation of the tree inferred from NJ analysis from the 258 non-redundant set of bZIPs from Arabidopsis, rice and black cottonwood using *p*-distance and 1000 bootstrap repetitions (indicated as percentages at the branch points). The alignment used corresponds to the minimum bZIP domain of 44 amino acids. Groups D and F are sister groups supported by a 50% bootstrap. Rice, black cottonwood and Arabidopsis sequences are represented in orange, dark blue and light blue, respectively.
(1.01 MB TIF)

Figure S2 Conserved intron position in the basic motif region of angiosperm bZIP transcription factors. The first leucine of the leucine zipper is highlighted in green, and the conserved asparagine of the basic motif is shown in red. According to the position of the introns, indicated by arrows, four different groups can be observed (1 to 4). bZIPs from Group L have a basic motif five amino acids shorter than that of the other bZIPs, and the conserved asparagine, shown in red, is substituted either by lysine (K) or arginine (R). In bold, the first amino acid after the intron. The *bZIP* genes used in this figure are: *AtbZIP24* (Group F), *AtbZIP45* (Group D), *AtbZIP39* (Group A), *AtbZIP54* (Group G), *AtbZIP62* (Group J), *AtbZIP63* (Group C), *AtbZIP56* (Group H), *AtbZIP61* (Group E), *AtbZIP31* (Group I), *AtbZIP60* (Group K), *AtbZIP76* (Group L), *AtbZIP70* (Group S), and *AtbZIP49* (Group B).
(1.85 MB TIF)

Figure S3 Unrooted phylogenetic tree inferred from a NJ analysis from a subset of 173 bZIPs of Arabidopsis, rice and black cottonwood using *p*-distance and 1000 bootstrap repetitions (indicated as percentages at the branches). The alignment used corresponds to the minimal bZIP domain extended by two leucine repetitions, totaling 60 amino acids. Groups B, K and H, as well as Groups E and L are sister groups supported by bootstrap analysis. Rice, black cottonwood and Arabidopsis sequences are represented in orange, dark blue and light blue, respectively.
(1.11 MB TIF)

Figure S4 Phylogenetic tree of monocot and eudicot bZIPs of Group A. The unrooted tree was inferred by a NJ analysis from distances calculated with the PAM distance matrix. The bootstrap values correspond to 1000 repetitions and are indicated as percentage in every branch. The amino acid alignment used to generate this tree corresponds to the bZIP domain plus the conserved motif A1 (Figure 2 and Table S2). Rice, black cottonwood and Arabidopsis sequences are represented in orange, dark blue and light blue, respectively. Other eudicot sequences are shown in green. The organism from which the remaining monocot and eudicot bZIPs originated is indicated by the last two letters in each sequence. Abbreviations are explained in Table S6.
(1.28 MB TIF)

Figure S5 Phylogenetic tree of Group B bZIPs from monocots and eudicots. An unrooted tree was inferred by a NJ analysis from distances obtained from the PAM distance matrix. The bootstrap values correspond to 1000 repetitions and are indicated as percentage in every branch. The amino acid alignment used to generate this tree corresponds to the bZIP domain plus the conserved motifs within this group (Figure 2 and Table S2). Rice, black cottonwood and Arabidopsis sequences are represented in orange, dark blue and light blue, respectively. Other monocot sequences are shown in red. The organism from which the remaining monocot and eudicot bZIPs originated is indicated by the last two letters in each sequence. Abbreviations are explained in Table S6.
(0.31 MB TIF)

Figure S6 Phylogenetic tree of Group C bZIPs from monocots and eudicots. An unrooted tree was inferred by a NJ analysis from distances calculated with the PAM distance matrix. The bootstrap values correspond to 1000 repetitions and are indicated as percentage in every branch. The amino acid alignment used to generate this tree corresponds to the bZIP domain plus the conserved motif within this group (Figure 2 and Table S2). Rice, black cottonwood and Arabidopsis sequences are represented in orange, dark blue and light blue, respectively. Other eudicot and monocot sequences are show in green and red, respectively. The organism from which the remaining monocot and eudicot bZIPs originated is indicated by the last two letters in each sequence. Abbreviations are explained in Table S6.
(2.03 MB TIF)

Figure S7 Phylogenetic tree of Group D bZIPs from monocots and eudicots. An unrooted tree was inferred by a NJ analysis from distances calculated with the PAM distance matrix. The bootstrap values correspond to 1000 repetitions and are indicated as percentage in every branch. The amino acid alignment used to generate this tree corresponds to the bZIP domain plus the conserved motifs within this group (Figure 2 and Table S2). Rice, black cottonwood and Arabidopsis sequences are represented in orange, dark blue and light blue, respectively. Other eudicot and monocot sequences are show in green and red, respectively. The organism from which the remaining monocot and eudicot bZIPs originated is indicated by the last two letters in each sequence. Abbreviations are explained in Table S6.
(1.31 MB TIF)

Figure S8 Phylogenetic tree of Group E bZIPs from monocots and eudicots. An unrooted tree was inferred by a NJ analysis from distances calculated with the PAM distance matrix. The bootstrap values correspond to 1000 repetitions and are indicated as percentage in every branch. The amino acid alignment used to generate this tree corresponds to the bZIP domain plus the conserved motifs within this group (Figure 2 and Table S2). Rice, black cottonwood and Arabidopsis sequences are represented in orange, dark blue and light blue, respectively. The organism from which the remaining monocot and eudicot bZIPs originated is indicated by the last two letters in each sequence. Abbreviations are explained in Table S6.
(0.31 MB TIF)

Figure S9 Phylogenetic tree of Group F bZIPs from monocots and eudicots. An unrooted tree was inferred by a NJ analysis from distances calculated with the PAM distance matrix. The bootstrap values correspond to 1000 repetitions and are indicated as percentage in every branch. The amino acid alignment used to generate this tree corresponds to the bZIP domain plus the conserved motif within this group (Figure 2 and Table S2). Rice, black cottonwood and Arabidopsis sequences are represented in orange, dark blue and light blue, respectively. Other eudicot and monocot sequences are show in green and red, respectively. The organism from which the remaining monocot and eudicot bZIPs originated is indicated by the last two letters in each sequence. Abbreviations are explained in Table S6.
(0.83 MB TIF)

Figure S10 Phylogenetic tree of Group G bZIPs from monocots and eudicots. An unrooted tree was inferred by a NJ analysis from distances calculated with the PAM distance matrix. The bootstrap values correspond to 1000 repetitions and are indicated as percentage in every branch. The amino acid alignment used to generate this tree corresponds to the bZIP domain plus the conserved motifs within this group (Figure 2 and Table S2). Rice, black cottonwood and Arabidopsis sequences are represented in orange, dark blue and light blue, respectively. Other eudicot and monocot sequences are show in green and red, respectively. The organism from which the remaining monocot and eudicot bZIPs originated is indicated by the last two letters in each sequence. Abbreviations are explained in Table S6.
(1.03 MB TIF)

Figure S11 Phylogenetic tree of Group H bZIPs from monocots and eudicots. An unrooted tree was inferred by a NJ analysis from distances obtained from a PAM distance matrix. The bootstrap values correspond to 1000 repetitions and are indicated as percentage in every branch. The amino acid alignment used to generate this tree corresponds to the bZIP domain plus the conserved motif within this group (Figure 2 and Table S2). Rice, black cottonwood and Arabidopsis sequences are represented in orange, dark blue and light blue, respectively. Other eudicot and monocot sequences are show in green and red, respectively. The organism from which the remaining monocot and eudicot bZIPs originated is indicated by the last two letters in each sequence. Abbreviations are explained in Table S6.
(0.85 MB TIF)

Figure S12 Phylogenetic tree of Group I bZIPs from monocots and eudicots. An unrooted tree was inferred by a NJ analysis from distances obtained from a PAM distance matrix. The bootstrap values correspond to 1000 repetitions and are indicated as percentage in every branch. The amino acid alignment used to generate this tree corresponds to the bZIP domain plus the conserved motifs within this group (Figure 2 and Table S2). Rice, black cottonwood and Arabidopsis sequences are represented in orange, dark blue and light blue, respectively. Other eudicot sequences are show in green. The organism from which the remaining monocot and eudicot bZIPs originated is indicated by the last two letters in each sequence. Abbreviations are explained in Table S6.
(1.12 MB TIF)

Figure S13 Phylogenetic tree of Group J bZIPs from monocots and eudicots. An unrooted tree was inferred by a NJ analysis from distances obtained from a PAM distance matrix. The bootstrap values correspond to 1000 repetitions and are indicated as percentage in every branch. The amino acid alignment used to generate this tree corresponds to the bZIP domain plus the conserved motifs within this group (Figure 2 and Table S2). Rice, black cottonwood and Arabidopsis sequences are represented in orange, dark blue and light blue, respectively. The organism from which the remaining monocot and eudicot bZIPs originated is indicated by the last two letters in each sequence. Abbreviations are explained in Table S6.
(0.14 MB TIF)

Figure S14 Phylogenetic tree of Group K bZIPs from monocots and eudicots. An unrooted tree was inferred by a NJ analysis from distances obtained from a PAM distance matrix. The bootstrap values correspond to 1000 repetitions and are indicated as percentage in every branch. The amino acid alignment used to generate this tree corresponds to the bZIP domain plus the conserved motif within this group (Figure 2 and Table S2). Rice, black cottonwood and Arabidopsis sequences are represented in orange, dark blue and light blue, respectively. Other eudicots and monocot sequences are show in green and red, respectively. The organism from which the remaining monocot and eudicot bZIPs originated is indicated by the last two letters in each sequence. Abbreviations are explained in Table S6.
(0.82 MB TIF)

Figure S15 Phylogenetic tree of Group L bZIPs from monocots and eudicots. An unrooted tree was inferred by a NJ analysis from distances obtained from a PAM distance matrix. The bootstrap values correspond to 1000 repetitions and are indicated as percentage in every branch. The amino acid alignment used to generate this tree corresponds to the bZIP domain plus the conserved motifs within this group (Figure 2 and Table S2). Rice, black cottonwood and Arabidopsis sequences are represented in orange, dark blue and light blue, respectively. The organism from which the remaining monocot and eudicot bZIPs originated is indicated by the last two letters in each sequence. Abbreviations are explained in Table S6.
(0.47 MB TIF)

Figure S16 Phylogenetic tree of Group S bZIPs from monocots and eudicots. An unrooted tree was inferred by a NJ analysis from distances obtained from a PAM distance matrix. The bootstrap values correspond to 1000 repetitions and are indicated as percentage in every branch. The amino acid alignment used to generate this tree corresponds to the bZIP domain. Rice, black cottonwood and Arabidopsis sequences are represented in orange, dark blue and light blue, respectively. Other eudicot and monocot sequences are show in green and red, respectively. The organism from which the remaining monocot and eudicot bZIPs originated is indicated by the last two letters in each sequence. Abbreviations are explained in Table S6.
(2.04 MB TIF)

Figure S17 Gene amplification pattern in each angiosperm group of bZIP homologues.
(0.77 MB TIF)

Figure S18 Identification of Groups cI and cII. Both trees are a partial representation of the whole tree obtained by NJ analyses. (A) In the initial phylogenetic analysis with the complete ViridiZIP set, we were able to identify two clusters of genes that did not posses any member from angiosperms; therefore, we called them NA (non-angiosperm). (B) Restricted analyses including bZIPs from algae and mosses uncovered the relationship of Groups NA and C; both groups share the same homologue in Ostreococcus (*OtbZIP5*), indicating it to be a common ancestor. Group NA was re-classified into Groups cI and cII. Their relation to members of Group NA shown in (A) is indicated by stars (* for Group cII, or ** for Group cI). Groups cI, cII, C and OtbZIP5 form the Group Proto-C. The bootstrap support of each group is shown in the figure.
(2.44 MB TIF)

Figure S19 Evolution of the bZIP family of transcription factors in land plants. We estimated the birth-and-death parameter (λ) using CAFE, as described in Materials and Methods. (A) The examined values of λ ranged from 1.0×10^−4^ to 6.8×10^−3^. The log probabilities obtained for each assayed value are shown. The shadowed region is displayed at a higher scale in the inset, where a peak at λ = 0.002011 is observed. (B) Evolutionary relationships of land plants with divergence time points (Arabidopsis - black cottonwood, 100–120 million years ago (mya) (47); monocot - eudicot, 140–150 mya (57); Physcomitrella - angiosperms, 450 mya (58)). Numbers at the branch end points indicate the numbers of bZIPs observed in the extant species. Numbers at the nodes represent the expected number of bZIPs in the ancestral species. Using the three methods available in CAFE, i.e., Viterbi assignments, branch cutting and likelihood ratio test, we identified branches deviating from the background model. According to all three methods, the branch leading to angiosperms significantly deviates from the null model (*p*<0.05), which implies that there was a significant increase in the number of bZIPs in the lineage leading to that group. Similarly, the Viterbi and branch cutting methods identify the branch leading to bryophytes (Physcomitrella) exhibiting a significant reduction in the number of bZIPs (*p*<0.05). Finally, we did not observe any significant deviation of the model for the extant group of angiosperms which can be interpreted as an even diffusion of the number of bZIPs in each branch. However, one cannot exclude the effect of natural selection in accounting for the differences that are nevertheless occurring. The increased number of bZIPs in the branch leading to angiosperms might be, at least partly, related to the several genome-wide duplication events that took place in the history of that lineage.
(1.62 MB TIF)

Figure S20 Scheme of the pipeline for bZIP identification in genomic sequences and ESTs. (I) Input genomic and EST sequences are compared by tblastn with the Angiotot protein dataset, generating a group of sequences that putatively code for bZIPs (SeqZIP). (II) Manual curation allowed subtracting sequences already present in Angiotot (redundancies) and false positives, which mainly correspond to low-complexity sequences. (III) The remaining sequences (true positives) are compared by tblastx against the best hit from Angiotot obtained in step I, allowing to identify the most probable ORF, and in the case of genomic sequences, to identify their gene structure, taking into account conserved intron positions and the presence of canonic splicing sites (GT-AG).
(0.75 MB TIF)

Table S1 Comparison between bZIPs reported in this manuscript and in Nijhawan et al. (2008)
(0.04 MB XLS)

Table S2 Conserved motifs in bZIP PoGOs.
(0.01 MB PDF)

Table S3 Accession numbers and classification into groups of homologues of non-sequenced angiosperms.
(0.03 MB PDF)

Table S4 Biological functions of genes in PoGOs.
(0.02 MB PDF)

Table S5 Classification of non-angiosperm bZIPs.
(0.02 MB XLS)

Table S6 Organism abbreviations.
(0.03 MB XLS)

Table S7 Gene pairs resulting from segmental duplications of the Arabidopsis genome.
(0.03 MB DOC)

Dataset S1 Re-annotated nucleotide sequences from rice and black cottonwood.
(0.02 MB TXT)

Dataset S2 Re-annotated amino acid sequences from rice and black cottonwood.
(0.01 MB TXT)

Dataset S3 MPSS Expression data for bZIP genes from rice.
(0.02 MB PDF)

Dataset S4 MPSS Expression data for bZIP genes from Arabidopsis.
(0.01 MB PDF)

Text S1 Supporting texts including further results and discussion.
(0.06 MB DOC)
