# Supplementary figures and images for: The Role of bZIP Transcription Factors in Green Plant Evolution: Adaptive Features Emerging from Four Founder Genes
Source: PLoS One. 2008 Aug 13;3(8):e2944. doi: 10.1371/journal.pone.0002944 (PMC2492810; doi:10.1371/journal.pone.0002944)

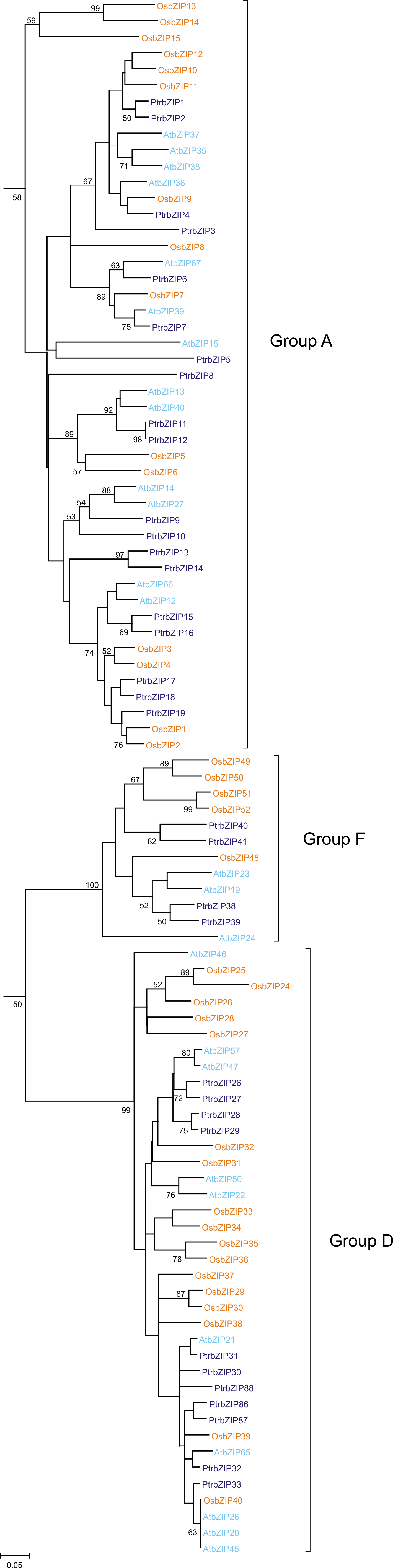

Supplement: Figure S1 — Definition of homologous gene groups A, D and F. This figure is a partial representation of the tree inferred from NJ analysis from the 258 non-redundant set of bZIPs from Arabidopsis, rice and black cottonwood using p-distance and 1000 bootstrap repetitions (indicated as percentages at the branch points). The alignment used corresponds to the minimum bZIP domain of 44 amino acids. Groups D and F are sister groups supported by a 50% bootstrap. Rice, black cottonwood and Arabidopsis sequences are represented in orange, dark blue and light blue, respectively. (1.01 MB TIF) [file pone.0002944.s001.tif]

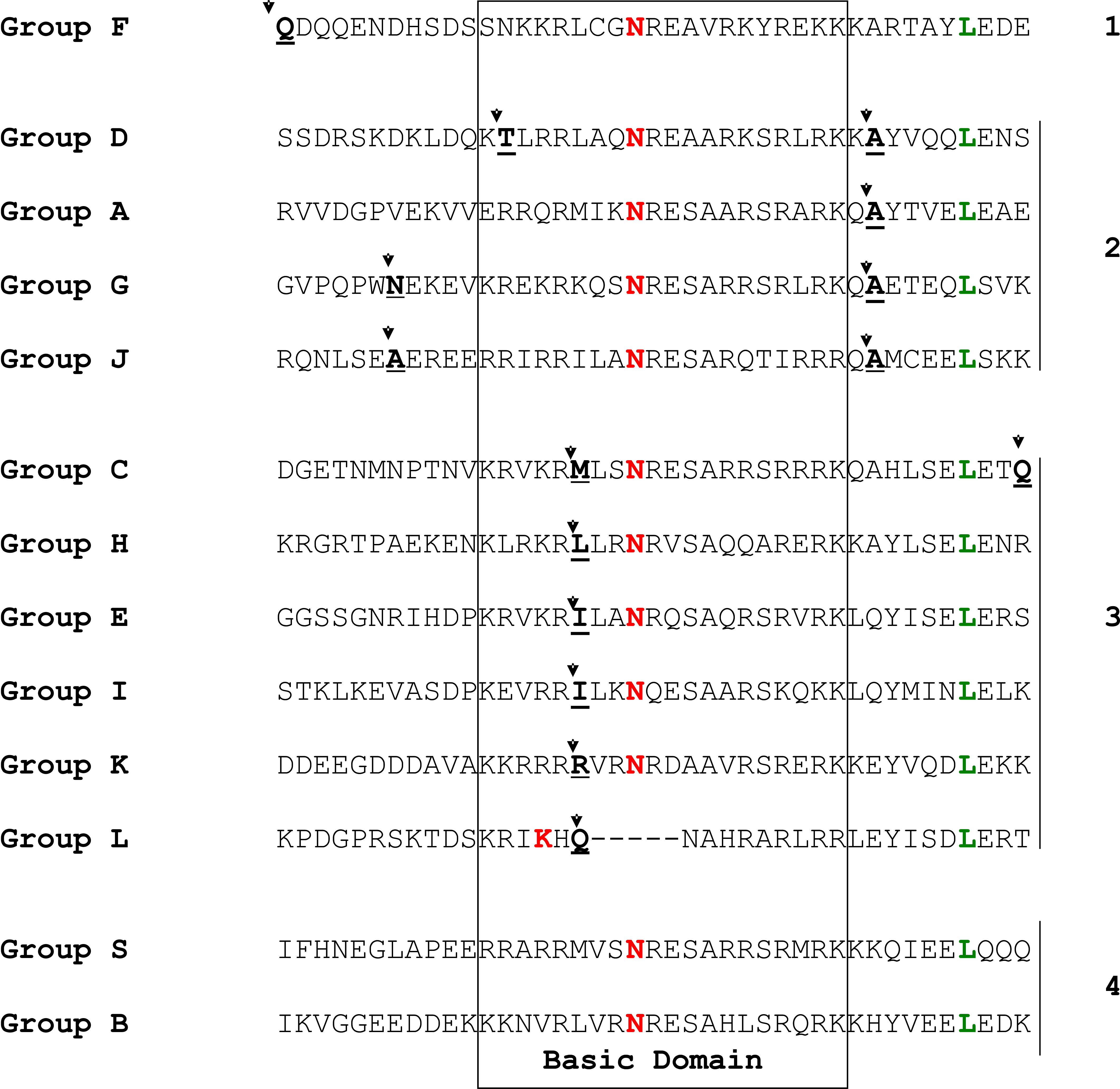

Supplement: Figure S2 — Conserved intron position in the basic motif region of angiosperm bZIP transcription factors. The first leucine of the leucine zipper is highlighted in green, and the conserved asparagine of the basic motif is shown in red. According to the position of the introns, indicated by arrows, four different groups can be observed (1 to 4). bZIPs from Group L have a basic motif five amino acids shorter than that of the other bZIPs, and the conserved asparagine, shown in red, is substituted either by lysine (K) or arginine (R). In bold, the first amino acid after the intron. The bZIP genes used in this figure are: AtbZIP24 (Group F), AtbZIP45 (Group D), AtbZIP39 (Group A), AtbZIP54 (Group G), AtbZIP62 (Group J), AtbZIP63 (Group C), AtbZIP56 (Group H), AtbZIP61 (Group E), AtbZIP31 (Group I), AtbZIP60 (Group K), AtbZIP76 (Group L), AtbZIP70 (Group S), and AtbZIP49 (Group B). (1.85 MB TIF) [file pone.0002944.s002.tif]

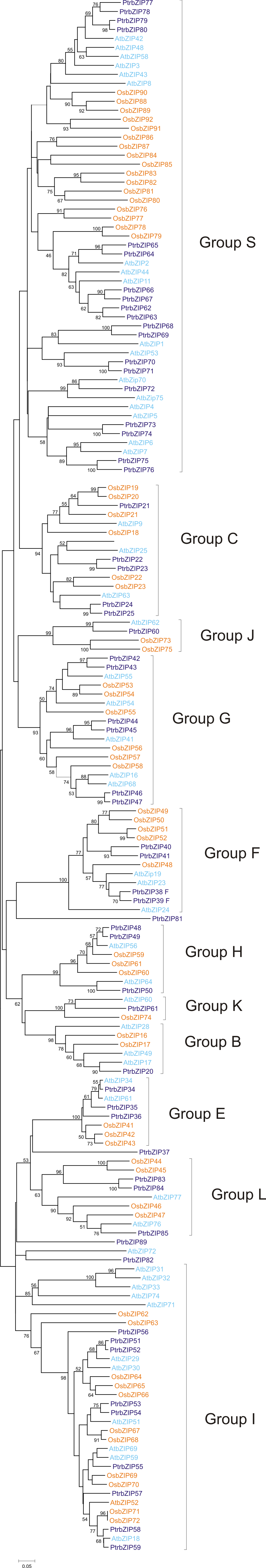

Supplement: Figure S3 — Unrooted phylogenetic tree inferred from a NJ analysis from a subset of 173 bZIPs of Arabidopsis, rice and black cottonwood using p-distance and 1000 bootstrap repetitions (indicated as percentages at the branches). The alignment used corresponds to the minimal bZIP domain extended by two leucine repetitions, totaling 60 amino acids. Groups B, K and H, as well as Groups E and L are sister groups supported by bootstrap analysis. Rice, black cottonwood and Arabidopsis sequences are represented in orange, dark blue and light blue, respectively. (1.11 MB TIF) [file pone.0002944.s003.tif]

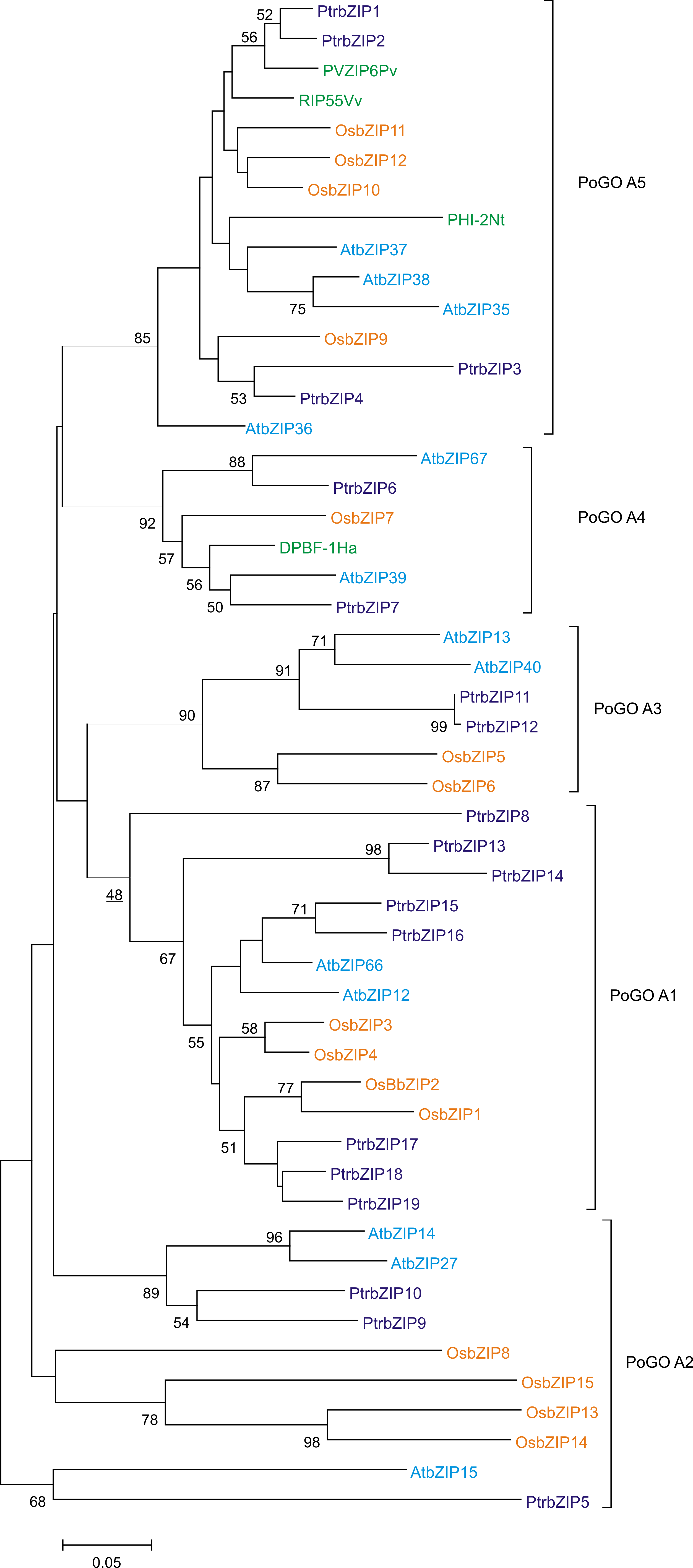

Supplement: Figure S4 — Phylogenetic tree of monocot and eudicot bZIPs of Group A. The unrooted tree was inferred by a NJ analysis from distances calculated with the PAM distance matrix. The bootstrap values correspond to 1000 repetitions and are indicated as percentage in every branch. The amino acid alignment used to generate this tree corresponds to the bZIP domain plus the conserved motif A1 (Figure 2 and Table S2). Rice, black cottonwood and Arabidopsis sequences are represented in orange, dark blue and light blue, respectively. Other eudicot sequences are shown in green. The organism from which the remaining monocot and eudicot bZIPs originated is indicated by the last two letters in each sequence. Abbreviations are explained in Table S6. (1.28 MB TIF) [file pone.0002944.s004.tif]

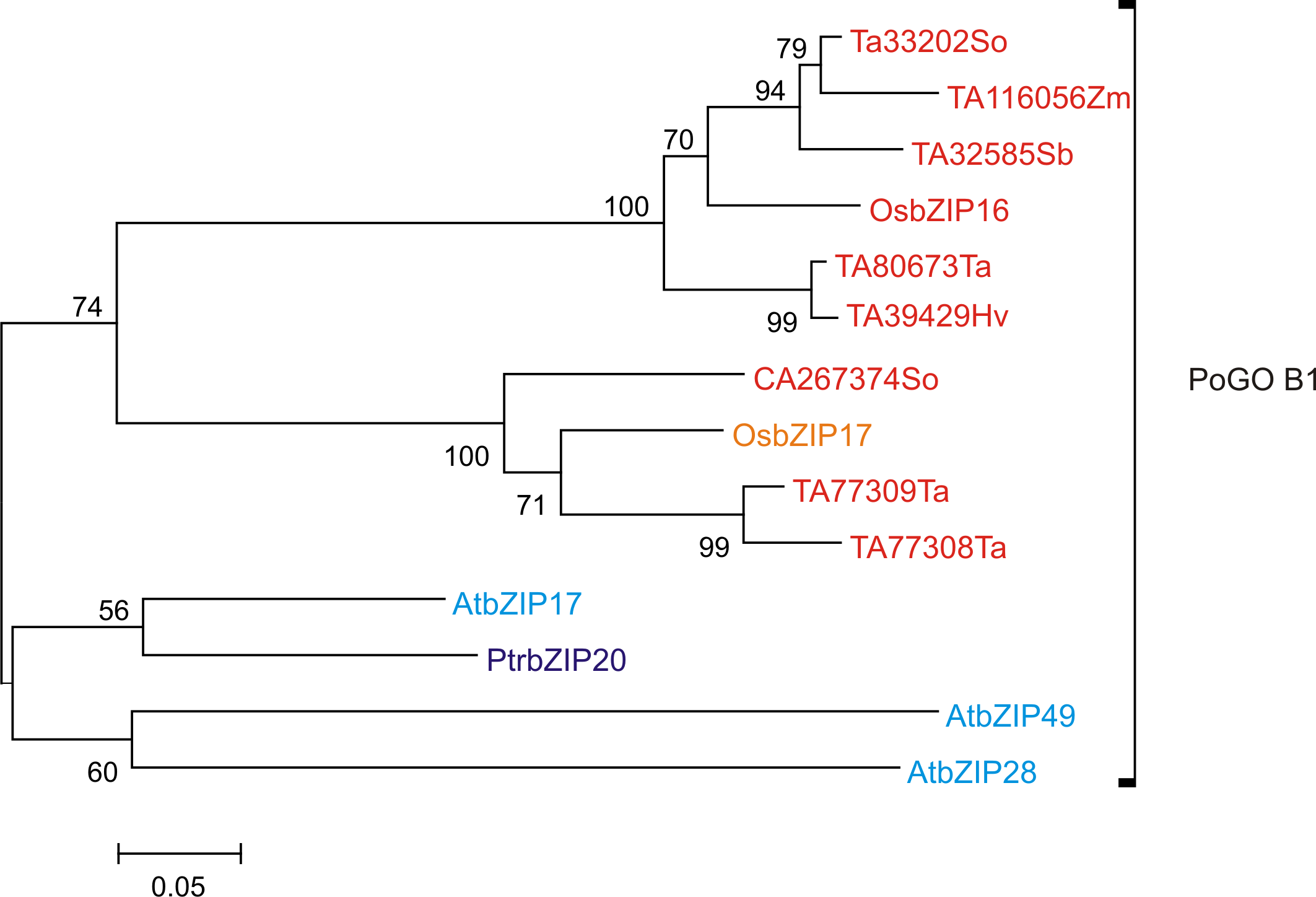

Supplement: Figure S5 — Phylogenetic tree of Group B bZIPs from monocots and eudicots. An unrooted tree was inferred by a NJ analysis from distances obtained from the PAM distance matrix. The bootstrap values correspond to 1000 repetitions and are indicated as percentage in every branch. The amino acid alignment used to generate this tree corresponds to the bZIP domain plus the conserved motifs within this group (Figure 2 and Table S2). Rice, black cottonwood and Arabidopsis sequences are represented in orange, dark blue and light blue, respectively. Other monocot sequences are shown in red. The organism from which the remaining monocot and eudicot bZIPs originated is indicated by the last two letters in each sequence. Abbreviations are explained in Table S6. (0.31 MB TIF) [file pone.0002944.s005.tif]

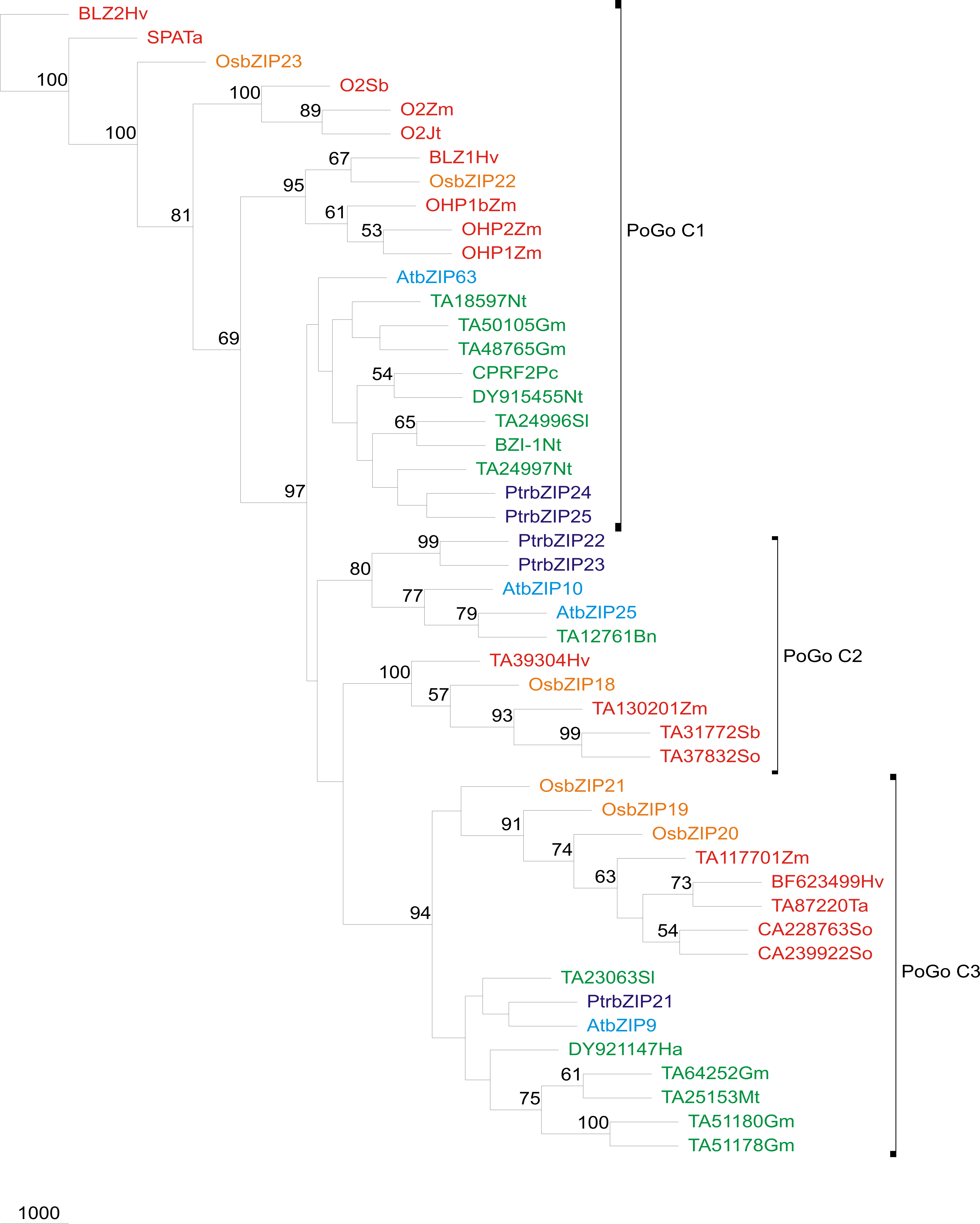

Supplement: Figure S6 — Phylogenetic tree of Group C bZIPs from monocots and eudicots. An unrooted tree was inferred by a NJ analysis from distances calculated with the PAM distance matrix. The bootstrap values correspond to 1000 repetitions and are indicated as percentage in every branch. The amino acid alignment used to generate this tree corresponds to the bZIP domain plus the conserved motif within this group (Figure 2 and Table S2). Rice, black cottonwood and Arabidopsis sequences are represented in orange, dark blue and light blue, respectively. Other eudicot and monocot sequences are show in green and red, respectively. The organism from which the remaining monocot and eudicot bZIPs originated is indicated by the last two letters in each sequence. Abbreviations are explained in Table S6. (2.03 MB TIF) [file pone.0002944.s006.tif]

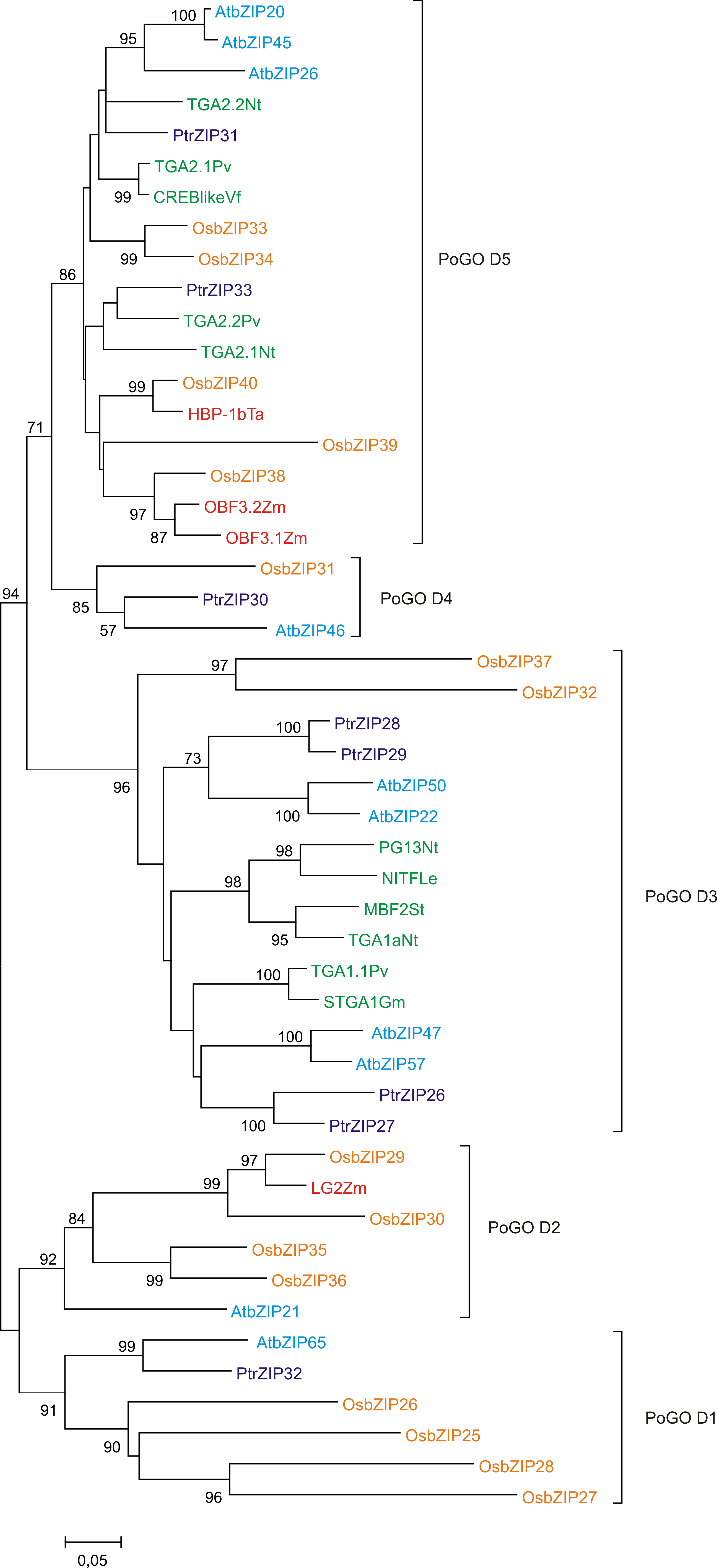

Supplement: Figure S7 — Phylogenetic tree of Group D bZIPs from monocots and eudicots. An unrooted tree was inferred by a NJ analysis from distances calculated with the PAM distance matrix. The bootstrap values correspond to 1000 repetitions and are indicated as percentage in every branch. The amino acid alignment used to generate this tree corresponds to the bZIP domain plus the conserved motifs within this group (Figure 2 and Table S2). Rice, black cottonwood and Arabidopsis sequences are represented in orange, dark blue and light blue, respectively. Other eudicot and monocot sequences are show in green and red, respectively. The organism from which the remaining monocot and eudicot bZIPs originated is indicated by the last two letters in each sequence. Abbreviations are explained in Table S6. (1.31 MB TIF) [file pone.0002944.s007.tif]

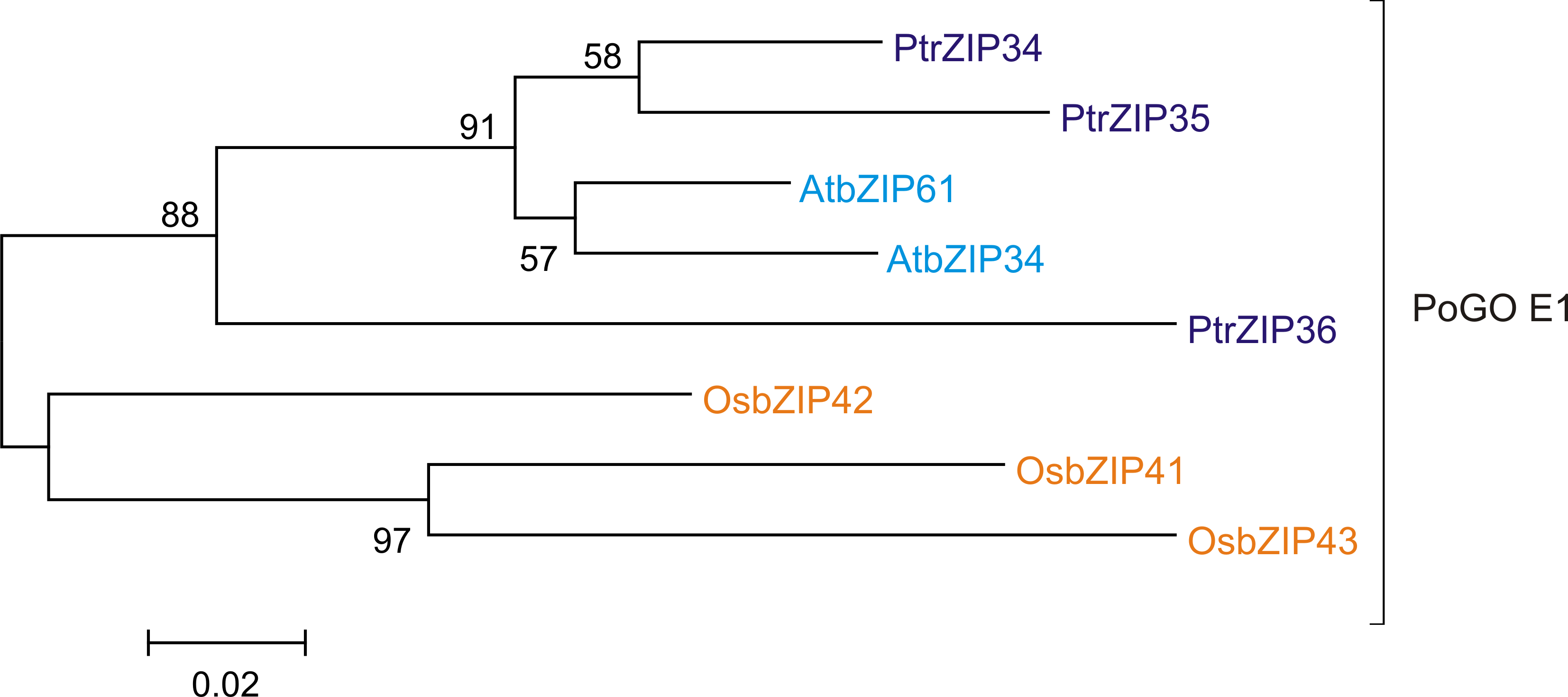

Supplement: Figure S8 — Phylogenetic tree of Group E bZIPs from monocots and eudicots. An unrooted tree was inferred by a NJ analysis from distances calculated with the PAM distance matrix. The bootstrap values correspond to 1000 repetitions and are indicated as percentage in every branch. The amino acid alignment used to generate this tree corresponds to the bZIP domain plus the conserved motifs within this group (Figure 2 and Table S2). Rice, black cottonwood and Arabidopsis sequences are represented in orange, dark blue and light blue, respectively. The organism from which the remaining monocot and eudicot bZIPs originated is indicated by the last two letters in each sequence. Abbreviations are explained in Table S6. (0.31 MB TIF) [file pone.0002944.s008.tif]

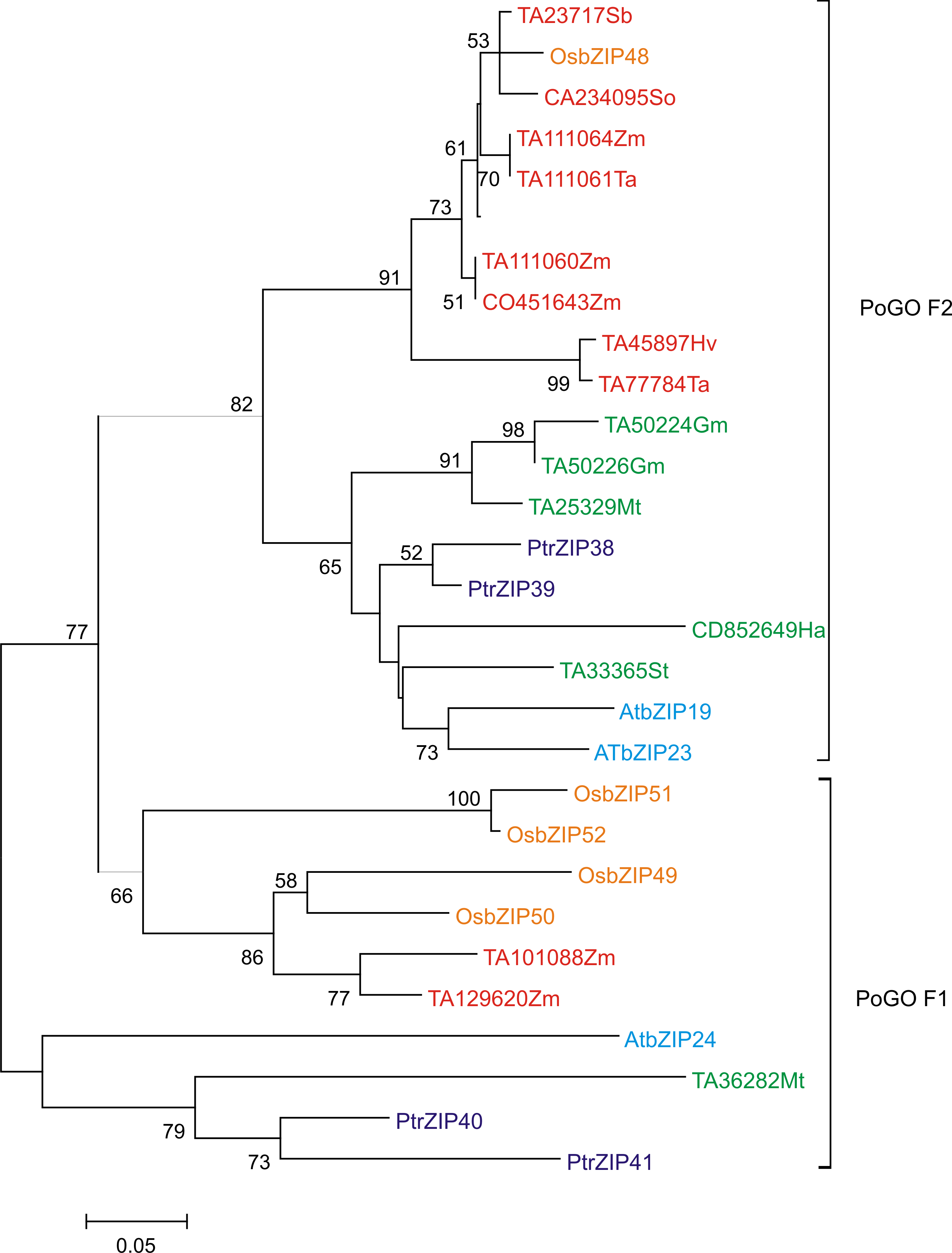

Supplement: Figure S9 — Phylogenetic tree of Group F bZIPs from monocots and eudicots. An unrooted tree was inferred by a NJ analysis from distances calculated with the PAM distance matrix. The bootstrap values correspond to 1000 repetitions and are indicated as percentage in every branch. The amino acid alignment used to generate this tree corresponds to the bZIP domain plus the conserved motif within this group (Figure 2 and Table S2). Rice, black cottonwood and Arabidopsis sequences are represented in orange, dark blue and light blue, respectively. Other eudicot and monocot sequences are show in green and red, respectively. The organism from which the remaining monocot and eudicot bZIPs originated is indicated by the last two letters in each sequence. Abbreviations are explained in Table S6. (0.83 MB TIF) [file pone.0002944.s009.tif]

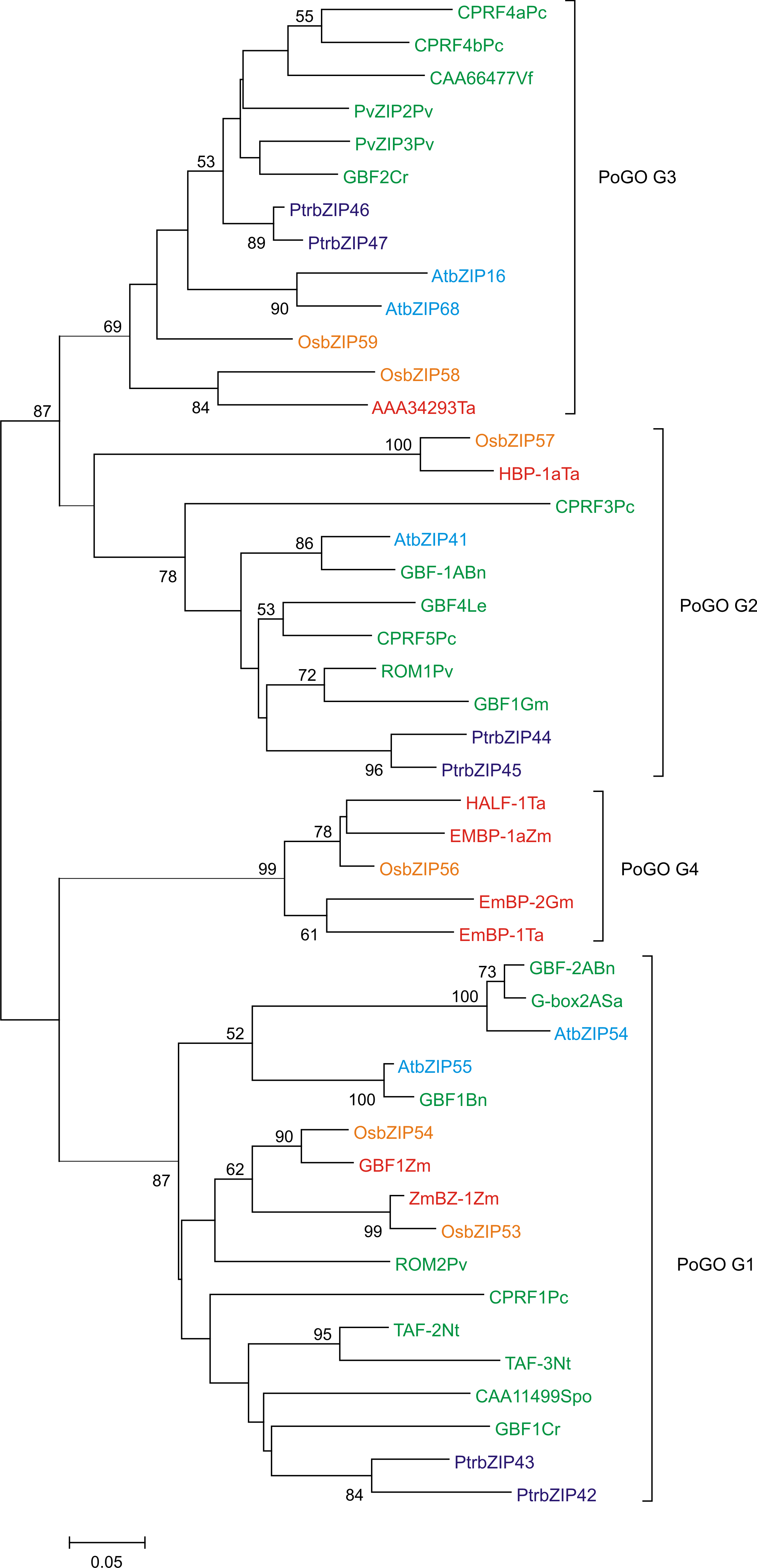

Supplement: Figure S10 — Phylogenetic tree of Group G bZIPs from monocots and eudicots. An unrooted tree was inferred by a NJ analysis from distances calculated with the PAM distance matrix. The bootstrap values correspond to 1000 repetitions and are indicated as percentage in every branch. The amino acid alignment used to generate this tree corresponds to the bZIP domain plus the conserved motifs within this group (Figure 2 and Table S2). Rice, black cottonwood and Arabidopsis sequences are represented in orange, dark blue and light blue, respectively. Other eudicot and monocot sequences are show in green and red, respectively. The organism from which the remaining monocot and eudicot bZIPs originated is indicated by the last two letters in each sequence. Abbreviations are explained in Table S6. (1.03 MB TIF) [file pone.0002944.s010.tif]

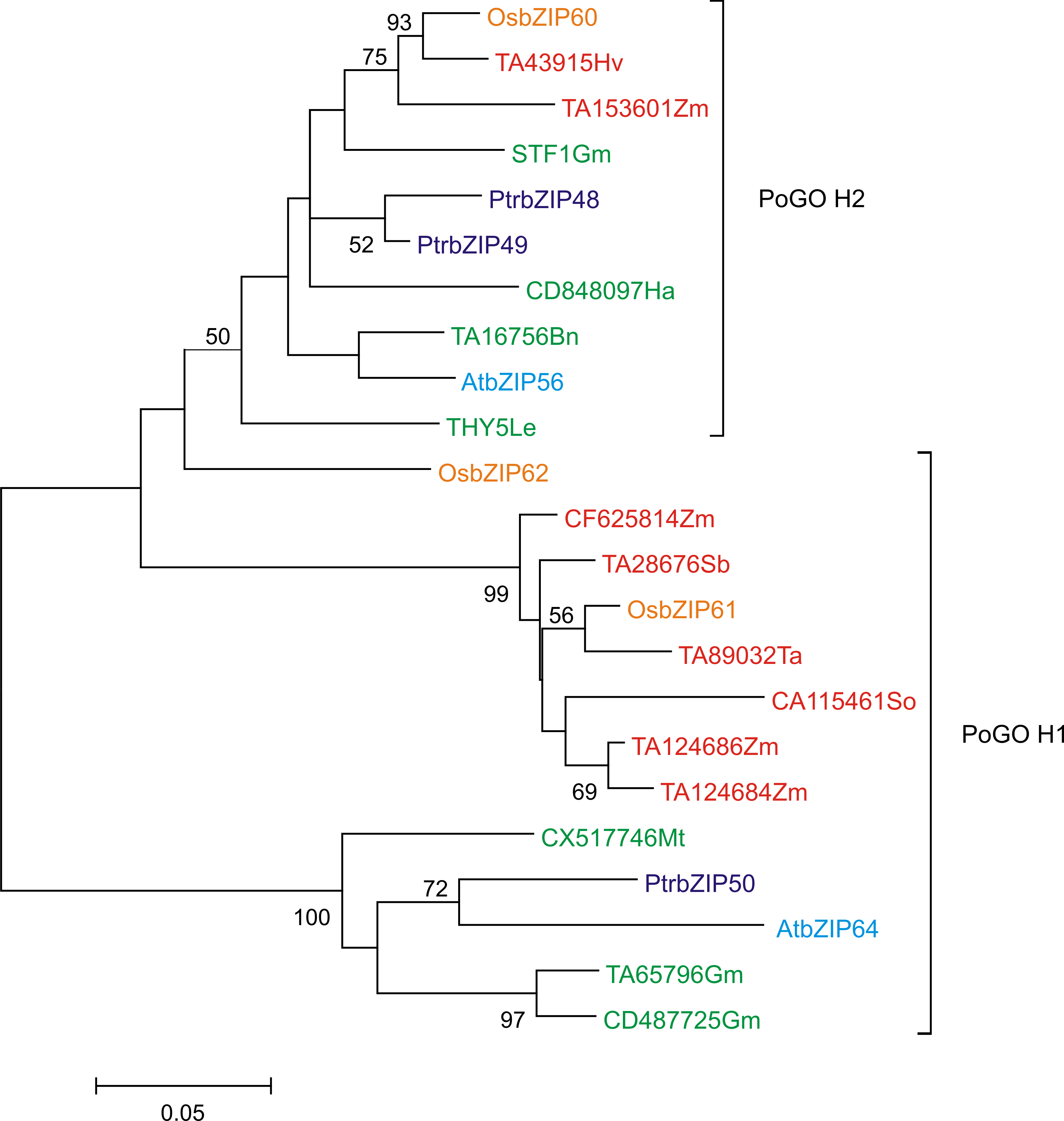

Supplement: Figure S11 — Phylogenetic tree of Group H bZIPs from monocots and eudicots. An unrooted tree was inferred by a NJ analysis from distances obtained from a PAM distance matrix. The bootstrap values correspond to 1000 repetitions and are indicated as percentage in every branch. The amino acid alignment used to generate this tree corresponds to the bZIP domain plus the conserved motif within this group (Figure 2 and Table S2). Rice, black cottonwood and Arabidopsis sequences are represented in orange, dark blue and light blue, respectively. Other eudicot and monocot sequences are show in green and red, respectively. The organism from which the remaining monocot and eudicot bZIPs originated is indicated by the last two letters in each sequence. Abbreviations are explained in Table S6. (0.85 MB TIF) [file pone.0002944.s011.tif]

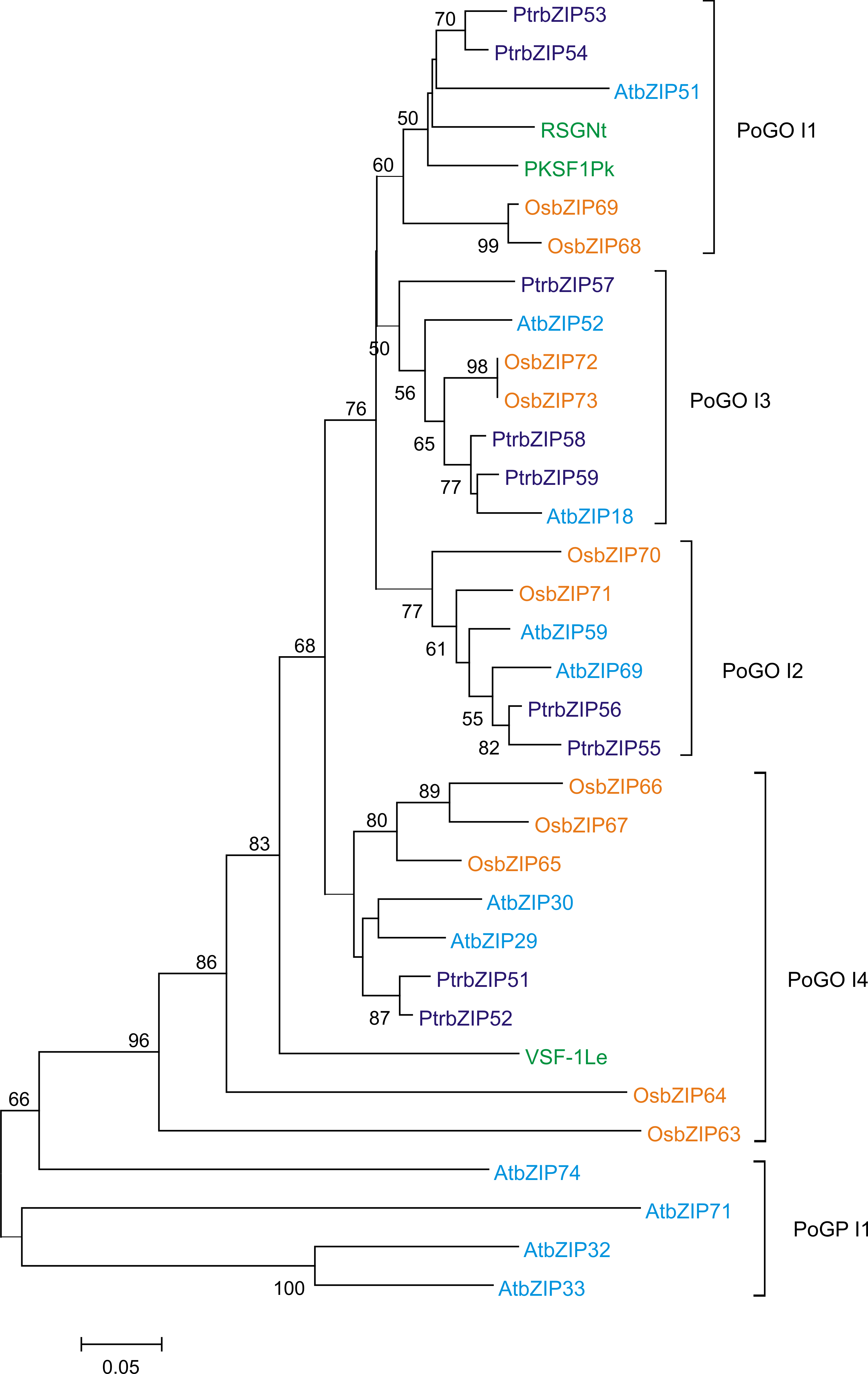

Supplement: Figure S12 — Phylogenetic tree of Group I bZIPs from monocots and eudicots. An unrooted tree was inferred by a NJ analysis from distances obtained from a PAM distance matrix. The bootstrap values correspond to 1000 repetitions and are indicated as percentage in every branch. The amino acid alignment used to generate this tree corresponds to the bZIP domain plus the conserved motifs within this group (Figure 2 and Table S2). Rice, black cottonwood and Arabidopsis sequences are represented in orange, dark blue and light blue, respectively. Other eudicot sequences are show in green. The organism from which the remaining monocot and eudicot bZIPs originated is indicated by the last two letters in each sequence. Abbreviations are explained in Table S6. (1.12 MB TIF) [file pone.0002944.s012.tif]

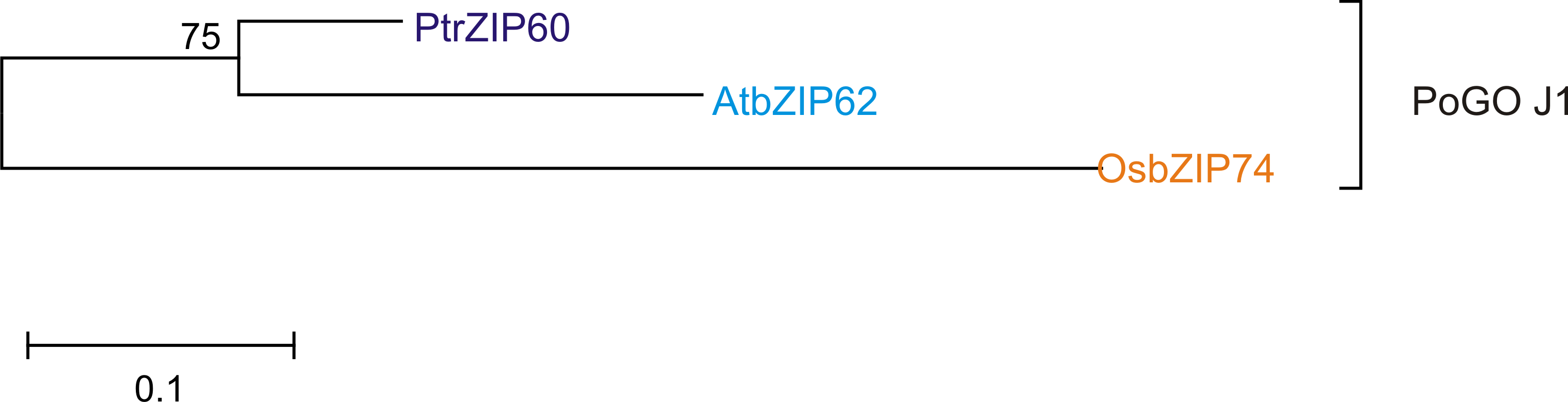

Supplement: Figure S13 — Phylogenetic tree of Group J bZIPs from monocots and eudicots. An unrooted tree was inferred by a NJ analysis from distances obtained from a PAM distance matrix. The bootstrap values correspond to 1000 repetitions and are indicated as percentage in every branch. The amino acid alignment used to generate this tree corresponds to the bZIP domain plus the conserved motifs within this group (Figure 2 and Table S2). Rice, black cottonwood and Arabidopsis sequences are represented in orange, dark blue and light blue, respectively. The organism from which the remaining monocot and eudicot bZIPs originated is indicated by the last two letters in each sequence. Abbreviations are explained in Table S6. (0.14 MB TIF) [file pone.0002944.s013.tif]

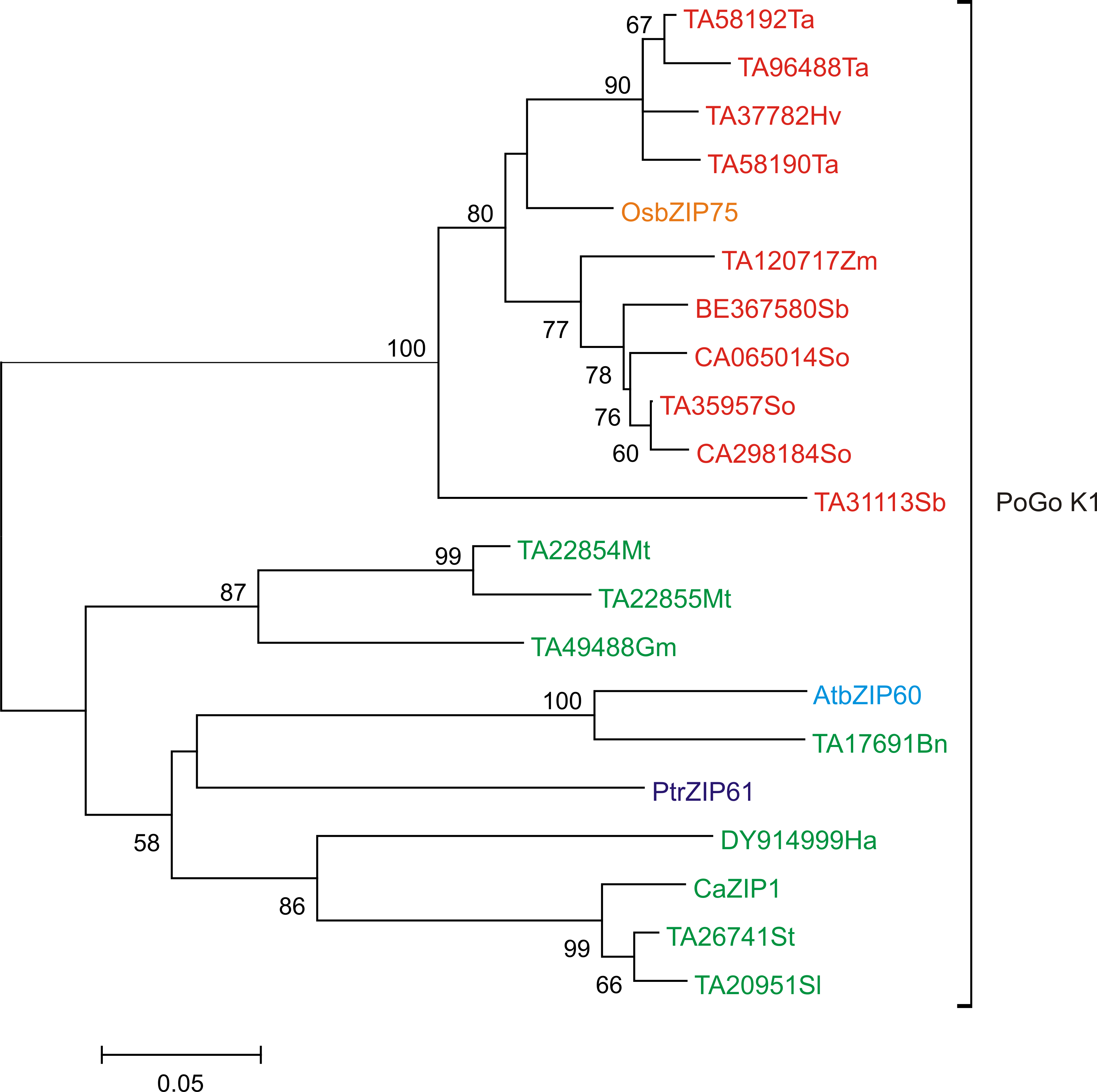

Supplement: Figure S14 — Phylogenetic tree of Group K bZIPs from monocots and eudicots. An unrooted tree was inferred by a NJ analysis from distances obtained from a PAM distance matrix. The bootstrap values correspond to 1000 repetitions and are indicated as percentage in every branch. The amino acid alignment used to generate this tree corresponds to the bZIP domain plus the conserved motif within this group (Figure 2 and Table S2). Rice, black cottonwood and Arabidopsis sequences are represented in orange, dark blue and light blue, respectively. Other eudicots and monocot sequences are show in green and red, respectively. The organism from which the remaining monocot and eudicot bZIPs originated is indicated by the last two letters in each sequence. Abbreviations are explained in Table S6. (0.82 MB TIF) [file pone.0002944.s014.tif]

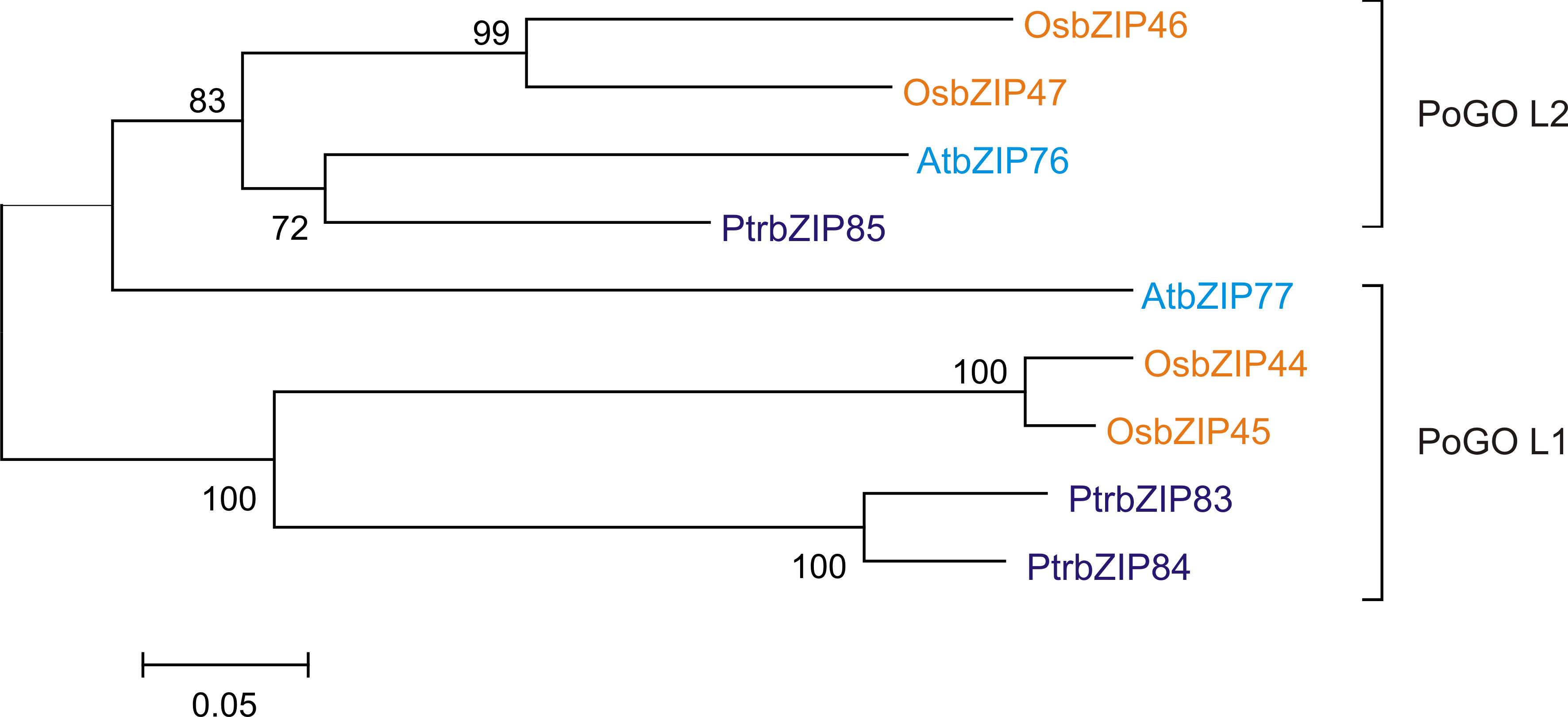

Supplement: Figure S15 — Phylogenetic tree of Group L bZIPs from monocots and eudicots. An unrooted tree was inferred by a NJ analysis from distances obtained from a PAM distance matrix. The bootstrap values correspond to 1000 repetitions and are indicated as percentage in every branch. The amino acid alignment used to generate this tree corresponds to the bZIP domain plus the conserved motifs within this group (Figure 2 and Table S2). Rice, black cottonwood and Arabidopsis sequences are represented in orange, dark blue and light blue, respectively. The organism from which the remaining monocot and eudicot bZIPs originated is indicated by the last two letters in each sequence. Abbreviations are explained in Table S6. (0.47 MB TIF) [file pone.0002944.s015.tif]

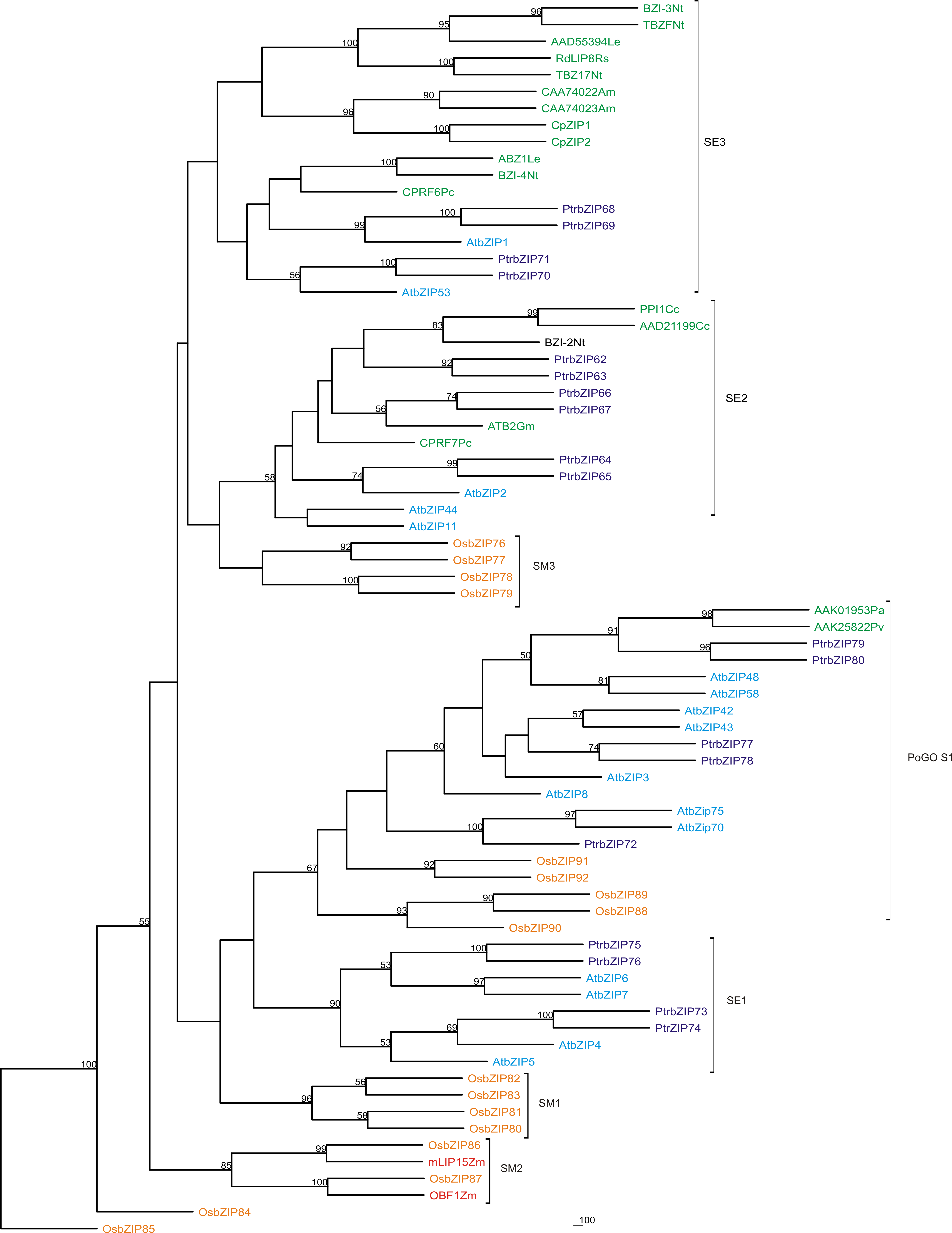

Supplement: Figure S16 — Phylogenetic tree of Group S bZIPs from monocots and eudicots. An unrooted tree was inferred by a NJ analysis from distances obtained from a PAM distance matrix. The bootstrap values correspond to 1000 repetitions and are indicated as percentage in every branch. The amino acid alignment used to generate this tree corresponds to the bZIP domain. Rice, black cottonwood and Arabidopsis sequences are represented in orange, dark blue and light blue, respectively. Other eudicot and monocot sequences are show in green and red, respectively. The organism from which the remaining monocot and eudicot bZIPs originated is indicated by the last two letters in each sequence. Abbreviations are explained in Table S6. (2.04 MB TIF) [file pone.0002944.s016.tif]

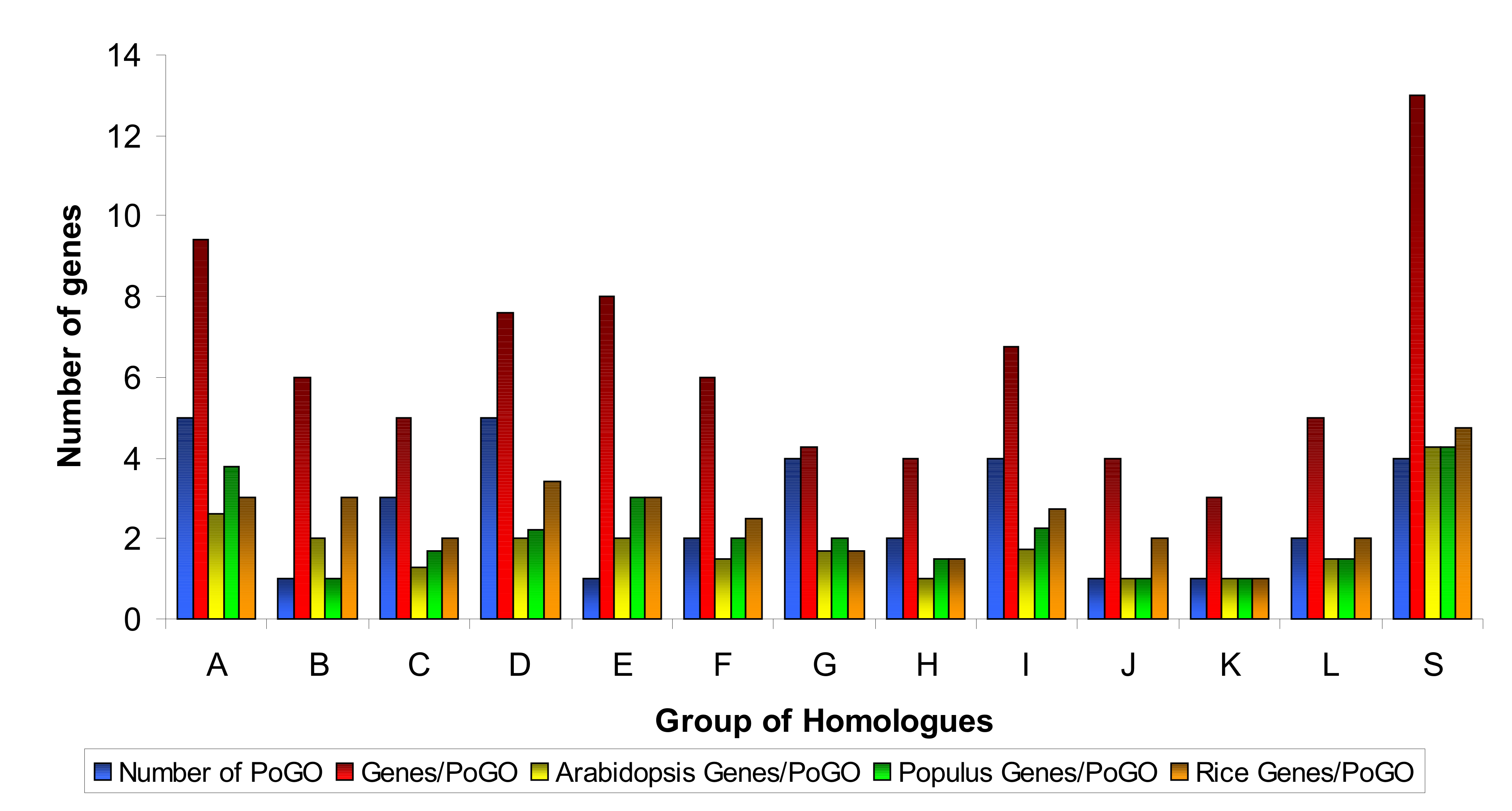

Supplement: Figure S17 — Gene amplification pattern in each angiosperm group of bZIP homologues. (0.77 MB TIF) [file pone.0002944.s017.tif]

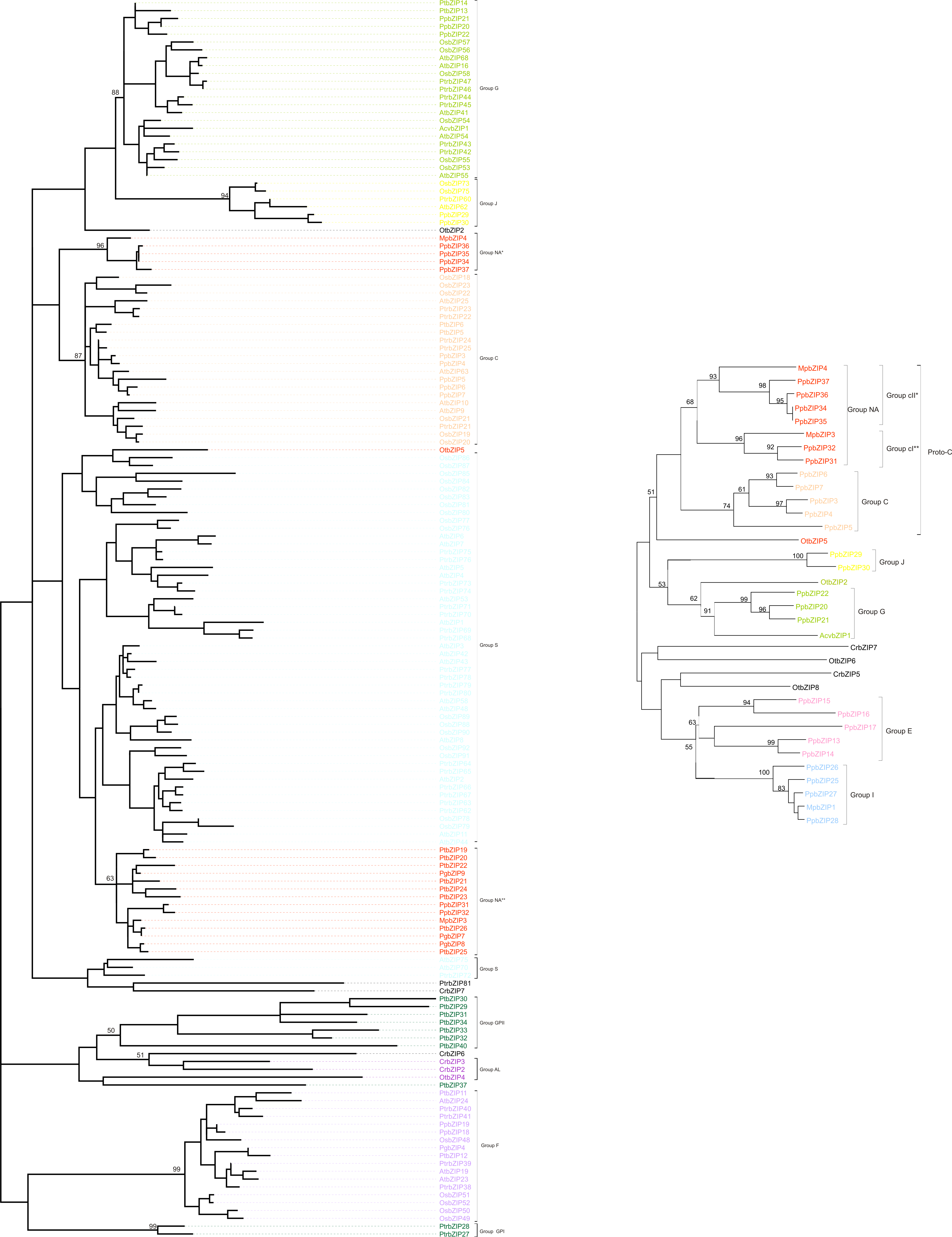

Supplement: Figure S18 — Identification of Groups cI and cII. Both trees are a partial representation of the whole tree obtained by NJ analyses. (A) In the initial phylogenetic analysis with the complete ViridiZIP set, we were able to identify two clusters of genes that did not posses any member from angiosperms; therefore, we called them NA (non-angiosperm). (B) Restricted analyses including bZIPs from algae and mosses uncovered the relationship of Groups NA and C; both groups share the same homologue in Ostreococcus (OtbZIP5), indicating it to be a common ancestor. Group NA was re-classified into Groups cI and cII. Their relation to members of Group NA shown in (A) is indicated by stars (* for Group cII, or ** for Group cI). Groups cI, cII, C and OtbZIP5 form the Group Proto-C. The bootstrap support of each group is shown in the figure. (2.44 MB TIF) [file pone.0002944.s018.tif]

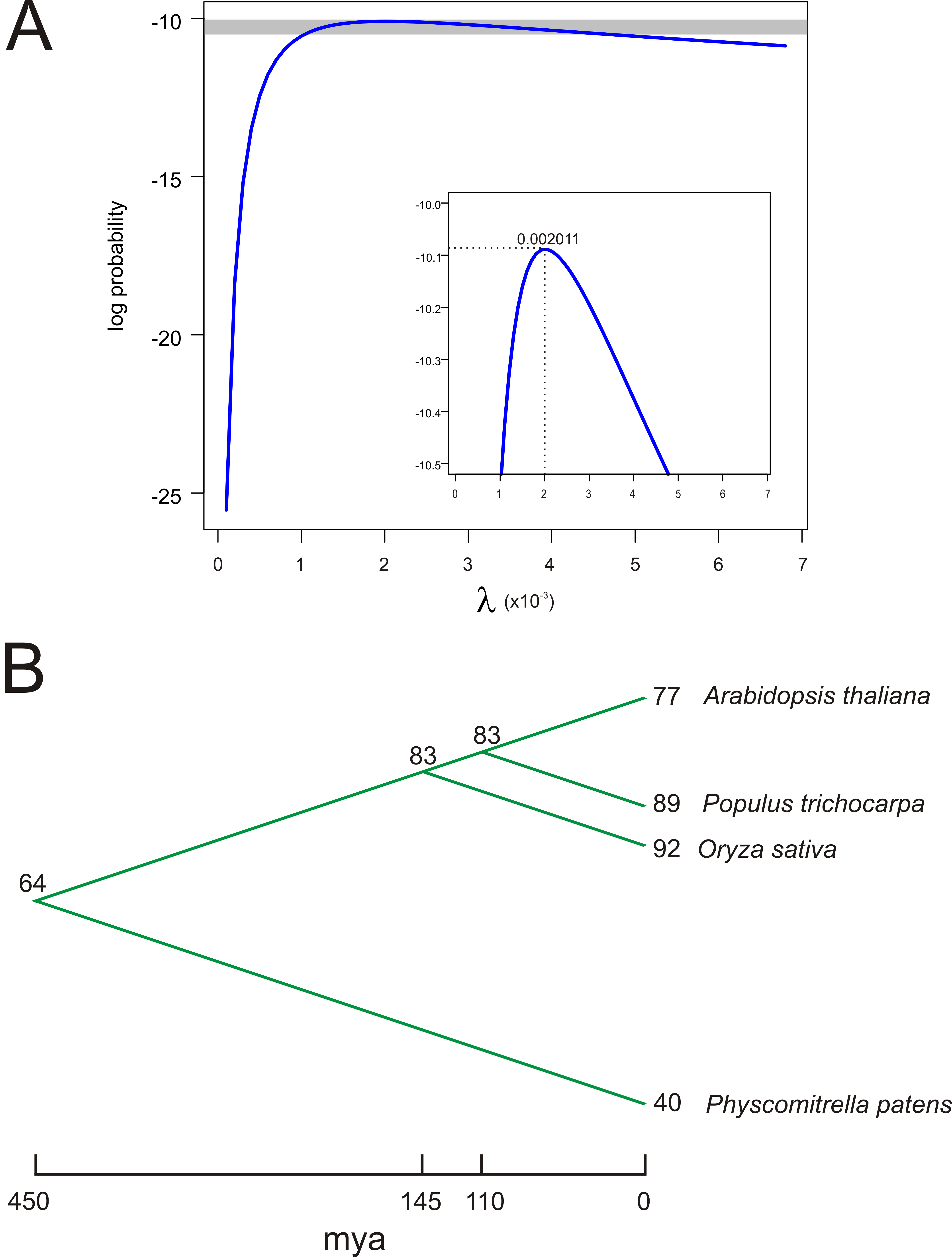

Supplement: Figure S19 — Evolution of the bZIP family of transcription factors in land plants. We estimated the birth-and-death parameter (λ) using CAFE, as described in Materials and Methods. (A) The examined values of λ ranged from 1.0×10^−4^ to 6.8×10^−3^. The log probabilities obtained for each assayed value are shown. The shadowed region is displayed at a higher scale in the inset, where a peak at λ = 0.002011 is observed. (B) Evolutionary relationships of land plants with divergence time points (Arabidopsis - black cottonwood, 100–120 million years ago (mya) (47); monocot - eudicot, 140–150 mya (57); Physcomitrella - angiosperms, 450 mya (58)). Numbers at the branch end points indicate the numbers of bZIPs observed in the extant species. Numbers at the nodes represent the expected number of bZIPs in the ancestral species. Using the three methods available in CAFE, i.e., Viterbi assignments, branch cutting and likelihood ratio test, we identified branches deviating from the background model. According to all three methods, the branch leading to angiosperms significantly deviates from the null model (p<0.05), which implies that there was a significant increase in the number of bZIPs in the lineage leading to that group. Similarly, the Viterbi and branch cutting methods identify the branch leading to bryophytes (Physcomitrella) exhibiting a significant reduction in the number of bZIPs (p<0.05). Finally, we did not observe any significant deviation of the model for the extant group of angiosperms which can be interpreted as an even diffusion of the number of bZIPs in each branch. However, one cannot exclude the effect of natural selection in accounting for the differences that are nevertheless occurring. The increased number of bZIPs in the branch leading to angiosperms might be, at least partly, related to the several genome-wide duplication events that took place in the history of that lineage. (1.62 MB TIF) [file pone.0002944.s019.tif]

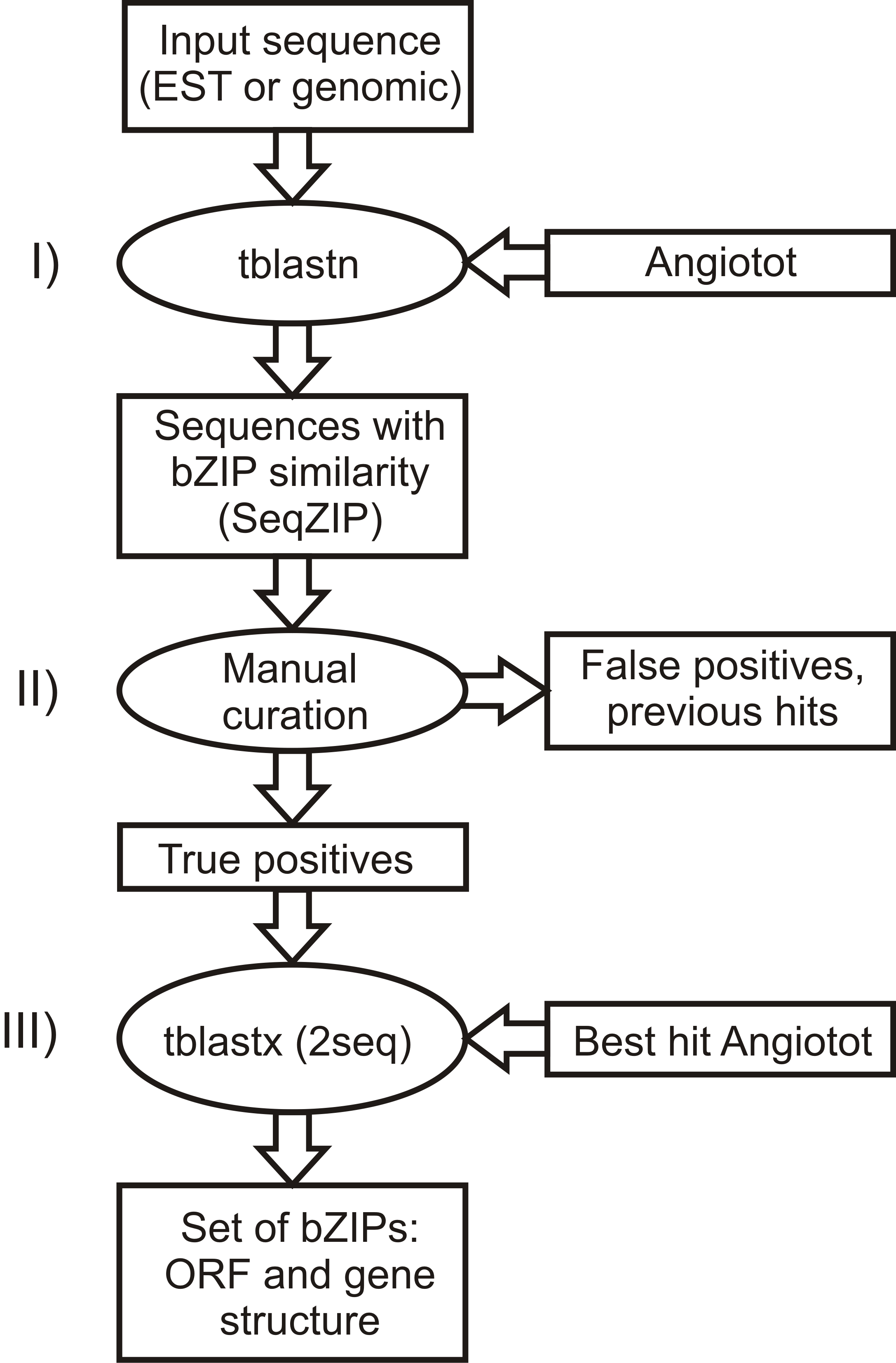

Supplement: Figure S20 — Scheme of the pipeline for bZIP identification in genomic sequences and ESTs. (I) Input genomic and EST sequences are compared by tblastn with the Angiotot protein dataset, generating a group of sequences that putatively code for bZIPs (SeqZIP). (II) Manual curation allowed subtracting sequences already present in Angiotot (redundancies) and false positives, which mainly correspond to low-complexity sequences. (III) The remaining sequences (true positives) are compared by tblastx against the best hit from Angiotot obtained in step I, allowing to identify the most probable ORF, and in the case of genomic sequences, to identify their gene structure, taking into account conserved intron positions and the presence of canonic splicing sites (GT-AG). (0.75 MB TIF) [file pone.0002944.s020.tif]
