# Supplementary material for: The Role of bZIP Transcription Factors in Green Plant Evolution: Adaptive Features Emerging from Four Founder Genes
Source: PLoS One. 2008 Aug 13;3(8):e2944. doi: 10.1371/journal.pone.0002944 (PMC2492810; doi:10.1371/journal.pone.0002944)
Supplement: Table S2 — Conserved motifs in bZIP PoGOs. (0.01 MB PDF) [file pone.0002944.s022.pdf]

Conserved domains in bZIP PoGOs			
**Motif**	**Pattern**	**Function**	**Observations**
A1	[NRST] [AIMV] [ADEG] [DEQ] [ILMV] [LW]	Putative kinase phosphorylation site	Not present in all members of the family (see main text)
A2	[ST] [IL] [EF] [DEQ] [DFL] [FL] [AFILSV] [KNQR]	Putative kinase phosphorylation site	Not present in all members of the family (see main text)
A3	LRRT[SL] [ST]		
B1	S[QH]VPLVPIPRLK[PT] [QK]Q		
B2	KTKKVAS[VI]S[LF]LGLL[FLC] [LV] [MA] [ML] [LV] [FC]G[CA] [LF] [VI]P[AG]VN[RHV] [MN] [YF] [GD]	transmembrane domain	
B3	P[GHQ]N[SAG]SE[TP]L[PV]A[LS]L[YF] [VL]PRN[GD]K[HL]VKI[ND]GNL[IV]I[HK]S[VI] [LV]ASEKA[SVR]		
B4	[DN] [GQ] [LQS] [KEI] [STY] [SR] [AES] [AD] [DN]G[KP] [LM] [PQ]QWF[RS]E[GA] [MV] [AS]GP[MIL] [LF] [SN]SGMC[TS]EVFQFD[VI]S[PS] [TA]		
B5	KNRRI[LM] [YR] [GNS]	S1P canonical site	
B6	[PS] [AV]SS[MV]VVSVL[AV]DPRE		
B7	[DI] [DG] [DG] [GMP]R[IG] [SGT] [PS]K[SP]LSR[IV]FVVVL[VL]D[SG]V[KR]YVTYSC[VT]LP		
C1	MNR[CS] [PA] [ST]EW[AY] [FL] [QE] [KR]F[LI] [EQ]E		not sequences all present this site, most probably they are incomplete sequences
D1	F[DE]MEY[AG] [RH]W[LV]EE[QH]N[RK]Q[IM]NELR[AT]A[LV] [NQ] [AS] [HQ] [AI] [GS]D		
D2	[ED]LRI[IL]V[DE]G[CVI] [ML]A		
D3	HYDE[LI]FR[LM]K[GAS]xAAKADVF[HY] [LV] [LM]SGMWKT[PS]AER[CF]F[LM]W[IL]GGFR[PS]SELLK[LV]L[AV] [PN] [QH]LEPLT[ED]QQL[LM]G[IV] [CY]NLQQS[SC]QQAE[DE]ALSQG		
E1	[NQ]P[SA]W[VA]DEFL[DG]F[SA] [AS] [TS]RRG[AT]HRRS[IV]SDS[IV]AFLE		
E2	[HG] [DE]FD[RK] [FL]DD[ED]Q[LF]MSMF[NS]DD		
F1	HTH[TA]C[TY]HTHT[CK] [NV]PP[GS]		This motif is present 2 times
G1	W[AS] [ASG] [MF]QAY[YS]		
G2	[HP]PYMWG[PV] [QP]		
G3	MPPYGTP[YP]		
H1	G[ME] [ED]SDEE[IL] [RG]RVP[ED] [MF] [GES] [GAL] [EA] [PAG] [AGP]G[TA]	COP1 interaction domain	
I1	F[SET]xA[ED]AKK[AI]M[AS] [PAD] [ED] [KR]LAE[LI]ALI		This motive is not present in PoGP I
J2	AAEAR[KR]RRKELT[KR]LK[NQ] [LM]HG[RG] [QG] [CG]		
K2	G[AV] [PSH] [TM] [AT] [MK]QESAVL[LTS] [EL] [TE]	transmembrane domain	
L1	[THP] [QGH] [HS] [HQC]R[TFS] [SP]S[EQ] [DSG] [SLPV] [FL] [IVL] [EGD] [ED] [QKP]P[SAC]WLDDLL[NSAD] [ED] [PES] [EDG] [AKT] [PSD] [AHSV] [RT] [GLPR] [HKP] [GCP] [LRH] [RP] [RG] [SHA] [ARCS] [SR]		
L2	DS[FDV] [AT] [LYI] [LF]D[GV]		
