# Supplementary material for: The Role of bZIP Transcription Factors in Green Plant Evolution: Adaptive Features Emerging from Four Founder Genes
Source: PLoS One. 2008 Aug 13;3(8):e2944. doi: 10.1371/journal.pone.0002944 (PMC2492810; doi:10.1371/journal.pone.0002944)
Supplement: Table S3 — Accession numbers and classification into groups of homologues of non-sequenced angiosperms. (0.03 MB PDF) [file pone.0002944.s023.pdf]

Accession numbers and classification into groups of homologues of non-sequenced angiosperms.

Species	Accession number	Group of homologues	Database version
Brassica napus	DY007004_1	A	v2
Brassica napus	TA14114_3708_1	A	v2
Brassica napus	TA9126_3708_3	A	v2
Brassica napus	TA16660_3708_3	A	v2
Brassica napus	CX191722_1	A	v2
Brassica napus	TA13612_3708_2	A	v2
Brassica napus	CD836876_2	B	v2
Brassica napus	TA12761_3708_2	C	v2
Brassica napus	TA16722_3708_3	C	v2
Brassica napus	CN727131_2	D	v2
Brassica napus	CN732338_1	D	v2
Brassica napus	TA17770_3708_3	D	v2
Brassica napus	CX193835_2	E	v2
Brassica napus	CD825777_3	F	v2
Brassica napus	TA12202_3708_1	G	v2
Brassica napus	BQ704223_2	G	v2
Brassica napus	CD818264_2	H	v2
Brassica napus	TA18042_3708_2	I	v2
Brassica napus	CN737772_1	I	v2
Brassica napus	TA17691_3708_3	K	v2
Brassica napus	CD833815_1	S	v2
Brassica napus	DY022281_3	S	v2
Brassica napus	TA16235_3708_3	S	v2
Brassica napus	TA14505_3708_3	S	v2
Brassica napus	CD841140_1	S	v2
Brassica napus	TA14216_3708_1	S	v2
Brassica napus	CX194685_2	S	v2
Brassica napus	CD818617_3	S	v2
Brassica napus	TA12114_3708_3	S	v2
Brassica napus	CN730710_1		v2
Glycine max	BG155969_2	A	v2
Glycine max	TA67133_3847_1	A	v2
Glycine max	AW394955_2	A	v2
Glycine max	TA58793_3847_1	A	v2
Glycine max	TA62305_3847_3	A	v2
Glycine max	BE608484_1	A	v2
Glycine max	TA73904_3847_3	A	v2
Glycine max	TA68805_3847_3	A	v2
Glycine max	TA68089_3847_2	A	v2
Glycine max	TA62306_3847_1	A	v2
Glycine max	TA68575_3847_2	A	v2
Glycine max	CO983201_3	A	v2
Glycine max	TA75637_3847_1	A	v2
Glycine max	TA50105_3847_3	C	v2
Glycine max	TA50103_3847_2	C	v2
Glycine max	TA48766_3847_2	C	v2
Glycine max	TA51179_3847_3	C	v2
Glycine max	TA50104_3847_2	C	v2
Glycine max	TA51180_3847_1	C	v2
Glycine max	TA51178_3847_1	C	v2
Glycine max	TA48765_3847_3	C	v2
Glycine max	Y10685_3	C	v2

Glycine max	TA64252_3847_1	C	v2
Glycine max	TA55785_3847_2	D	v2
Glycine max	BI498672_2	D	v2
Glycine max	AW277406_3	D	v2
Glycine max	TA52593_3847_3	D	v2
Glycine max	TA48457_3847_3	D	v2
Glycine max	TA61630_3847_3	D	v2
Glycine max	TA51570_3847_1	E	v2
Glycine max	TA51568_3847_3	E	v2
Glycine max	TA69507_3847_3	E	v2
Glycine max	TA57179_3847_1	E	v2
Glycine max	TA50226_3847_3	F	v2
Glycine max	BF066744_2	F	v2
Glycine max	TA50224_3847_1	F	v2
Glycine max	TA47380_3847_1	G	v2
Glycine max	BM092367_3	G	v2
Glycine max	TA51546_3847_1	G	v2
Glycine max	TA51543_3847_1	G	v2
Glycine max	TA54417_3847_2	G	v2
Glycine max	TA59011_3847_2	G	v2
Glycine max	BI316862_2	G	v2
Glycine max	TA63034_3847_1	G	v2
Glycine max	BF425919_1	G	v2
Glycine max	CD487725_2	H	v2
Glycine max	TA65796_3847_3	H	v2
Glycine max	TA48655_3847_2	I	v2
Glycine max	TA66143_3847_2	I	v2
Glycine max	TA54356_3847_3	I	v2
Glycine max	BE610799_2	I	v2
Glycine max	TA60949_3847_2	I	v2
Glycine max	CO984129_2	I	v2
Glycine max	TA48653_3847_1	I	v2
Glycine max	TA56480_3847_1	I	v2
Glycine max	TA60948_3847_3	I	v2
Glycine max	TA49488_3847_1	K	v2
Glycine max	BE346317_2	S	v2
Glycine max	TA64646_3847_2	S	v2
Glycine max	TA46007_3847_1	S	v2
Glycine max	TA68920_3847_3	S	v2
Glycine max	TA40538_3847_3	S	v2
Glycine max	TA46009_3847_1	S	v2
Glycine max	TA48767_3847_2	S	v2
Glycine max	TA57778_3847_1	S	v2
Glycine max	BE800665_3	S	v2
Glycine max	TA46010_3847_1	S	v2
Glycine max	TA57777_3847_1	S	v2
Glycine max	TA74725_3847_2	S	v2
Glycine max	TA57945_3847_2	S	v2
Glycine max	TA48379_3847_1	S	v2
Glycine max	TA48768_3847_3	S	v2
Glycine max	TA57944_3847_2	S	v2
Glycine max	TA48380_3847_1	S	v2
Glycine max	TA40702_3847_1	S	v2
Helianthus annus	TA14603_4232_1	A	V2
Helianthus annus	DY925853_1	A	V2
Helianthus annus	TA13904_4232_2	A	V2
Helianthus annus	TA16636_4232_3	A	V2
Helianthus annus	BU018591_2	C	V2

Helianthus annus	DY915455_1	C	V2
Helianthus annus	DY921147_1	C	V2
Helianthus annus	BU028485_1	D	V2
Helianthus annus	TA12815_4232_3	D	V2
Helianthus annus	DY915352_2	D	V2
Helianthus annus	CD852649_2	F	V2
Helianthus annus	TA11082_4232_3	G	V2
Helianthus annus	BQ966044_3	G	V2
Helianthus annus	DY916353_3	G	V2
Helianthus annus	TA16001_4232_2	G	V2
Helianthus annus	CD847895_2	G	V2
Helianthus annus	CD848097_2	H	V2
Helianthus annus	BQ970727_2	I	V2
Helianthus annus	DY914999_1	K	V2
Helianthus annus	BQ979657_1	S	V2
Helianthus annus	DY924177_3	S	V2
Helianthus annus	BQ916838_3	S	V2
Helianthus annus	CD848411_1	S	V2
Helianthus annus	TA15647_4232_2	S	V2
Helianthus annus	DY906840_2	S	V2
Helianthus annus	TA7778_4232_2	S	V2
Helianthus annus	TA14115_4232_3	S	V2
Helianthus annus	DY908163_1	S	V2
Medicado truncatula	CX535343_2	A	v2
Medicado truncatula	TA35601_3880_3	A	v2
Medicado truncatula	TA37027_3880_1	A	v2
Medicado truncatula	TA38803_3880_5	A	v2
Medicado truncatula	TA29491_3880_6	A	v2
Medicado truncatula	BI310847_2	A	v2
Medicado truncatula	TA18597_3880_1	C	v2
Medicado truncatula	TA24987_3880_1	C	v2
Medicado truncatula	TA25153_3880_2	C	v2
Medicado truncatula	TA38704_3880_1	C	v2
Medicado truncatula	TA21733_3880_2	D	v2
Medicado truncatula	TA33339_3880_1	D	v2
Medicado truncatula	TA37406_3880_1	D	v2
Medicado truncatula	TA32741_3880_3	D	v2
Medicado truncatula	CX541591_2	D	v2
Medicado truncatula	TA23383_3880_3	D	v2
Medicado truncatula	BG582106_2	D	v2
Medicado truncatula	TA28865_3880_1	D	v2
Medicado truncatula	TA36738_3880_2	D	v2
Medicado truncatula	TA31633_3880_3	D	v2
Medicado truncatula	TA22580_3880_2	E	v2
Medicado truncatula	TA34097_3880_2	E	v2
Medicado truncatula	TA25329_3880_2	F	v2
Medicado truncatula	TA36282_3880_1	F	v2
Medicado truncatula	BQ148978_2	G	v2
Medicado truncatula	AL388132_3	G	v2
Medicado truncatula	TA26173_3880_1	G	v2
Medicado truncatula	TA33144_3880_2	G	v2
Medicado truncatula	CX517746_2	H	v2
Medicado truncatula	AL384098_2	I	v2
Medicado truncatula	BE203302_2	I	v2
Medicado truncatula	TA21837_3880_2	I	v2
Medicado truncatula	TA24461_3880_2	I	v2
Medicado truncatula	TA22854_3880_1	K	v2
Medicado truncatula	TA21059_3880_2	S	v2

Medicado truncatula	TA20293_3880_1	S	v2
Medicado truncatula	DY617518_2	S	v2
Medicado truncatula	CB893389_1	S	v2
Medicado truncatula	DY615615_2	S	v2
Medicado truncatula	TA27861_3880_2	S	v2
Medicado truncatula	AW981132_2	S	v2
Medicado truncatula	TA34745_3880_3	S	v2
Medicado truncatula	BE943475_2	S	v2
Medicado truncatula	TA20855_3880_2	S	v2
Solanum lycopersicum	TA24850_4081_1	A	v2
Solanum lycopersicum	BI204084_2	A	v2
Solanum lycopersicum	BP891659_2	A	v2
Solanum lycopersicum	TA29189_4081_1	A	v2
Solanum lycopersicum	TA29037_4081_2	A	v2
Solanum lycopersicum	BP903433_2	A	v2
Solanum lycopersicum	TA23063_4081_3	C	v2
Solanum lycopersicum	TA31180_4081_1	C	v2
Solanum lycopersicum	TA20156_4081_3	C	v2
Solanum lycopersicum	TA26119_4081_3	D	v2
Solanum lycopersicum	AI488762_2	D	v2
Solanum lycopersicum	BG126810_2	D	v2
Solanum lycopersicum	TA32025_4081_3	D	v2
Solanum lycopersicum	BF097160_2	D	v2
Solanum lycopersicum	TA27092_4081_3	D	v2
Solanum lycopersicum	TA22555_4081_1	D	v2
Solanum lycopersicum	TA22556_4081_1	D	v2
Solanum lycopersicum	TA22087_4081_1	D	v2
Solanum lycopersicum	TA27093_4081_3	D	v2
Solanum lycopersicum	TA23862_4081_2	E	v2
Solanum lycopersicum	AW651406_2	E	v2
Solanum lycopersicum	TA31281_4081_1	E	v2
Solanum lycopersicum	TA27906_4081_3	F	v2
Solanum lycopersicum	BT013873_2	G	v2
Solanum lycopersicum	TA23032_4081_1	G	v2
Solanum lycopersicum	TA22459_4081_1	G	v2
Solanum lycopersicum	TA17494_4081_1	I	v2
Solanum lycopersicum	TA28075_4081_3	I	v2
Solanum lycopersicum	BF098455_3	I	v2
Solanum lycopersicum	TA25506_4081_1	I	v2
Solanum lycopersicum	TA20952_4081_2	K	v2
Solanum lycopersicum	TA20951_4081_2	K	v2
Solanum lycopersicum	TA28182_4081_2	S	v2
Solanum lycopersicum	TA18750_4081_3	S	v2
Solanum lycopersicum	TA29210_4081_2	S	v2
Solanum lycopersicum	TA32139_4081_2	S	v2
Solanum lycopersicum	TA27369_4081_2	S	v2
Solanum lycopersicum	TA18749_4081_1	S	v2
Solanum lycopersicum	TA23077_4081_1	S	v2
Solanum lycopersicum	BE450859_3	S	v2
Solanum tuberosum	CK862819_1	A	v2
Solanum tuberosum	TA27886_4113_6	A	v2
Solanum tuberosum	TA31280_4113_2	A	v2
Solanum tuberosum	TA37366_4113_1	A	v2
Solanum tuberosum	TA39708_4113_1	A	v2
Solanum tuberosum	TA44374_4113_2	A	v2
Solanum tuberosum	TA45822_4113_3	A	v2
Solanum tuberosum	TA47967_4113_1	A	v2
Solanum tuberosum	TA39703_4113_3	B	v2

Solanum tuberosum	DR036669_3	C	v2
Solanum tuberosum	TA24996_4113_1	C	v2
Solanum tuberosum	TA24997_4113_1	C	v2
Solanum tuberosum	CK265068_3	D	v2
Solanum tuberosum	CK266379_1	D	v2
Solanum tuberosum	CO501957_2	D	v2
Solanum tuberosum	TA32533_4113_3	D	v2
Solanum tuberosum	TA39272_4113_2	D	v2
Solanum tuberosum	TA43569_4113_2	D	v2
Solanum tuberosum	TA33365_4113_1	F	v2
Solanum tuberosum	CV468769_2	G	v2
Solanum tuberosum	TA28515_4113_1	G	v2
Solanum tuberosum	TA30257_4113_2	G	v2
Solanum tuberosum	TA35668_4113_1	G	v2
Solanum tuberosum	TA43344_4113_1	G	v2
Solanum tuberosum	TA43931_4113_3	G	v2
Solanum tuberosum	TA44025_4113_2	G	v2
Solanum tuberosum	TA49072_4113_2	G	v2
Solanum tuberosum	BG890987_3	H	v2
Solanum tuberosum	CK275965_1	I	v2
Solanum tuberosum	TA32440_4113_2	I	v2
Solanum tuberosum	TA35607_4113_2	I	v2
Solanum tuberosum	TA37376_4113_2	I	v2
Solanum tuberosum	TA37377_4113_1	I	v2
Solanum tuberosum	TA42125_4113_2	I	v2
Solanum tuberosum	TA26741_4113_3	K	v2
Solanum tuberosum	CK862038_1	S	v2
Solanum tuberosum	DN906956_2	S	v2
Solanum tuberosum	TA23494_4113_2	S	v2
Solanum tuberosum	TA23510_4113_3	S	v2
Solanum tuberosum	TA26759_4113_3	S	v2
Solanum tuberosum	TA27324_4113_3	S	v2
Solanum tuberosum	TA43690_4113_4	S	v2
Solanum tuberosum	TA48587_4113_2	S	v2
Hordeum vulgare	CV063683_3	A	v2
Hordeum vulgare	TA36491_4513_2	A	v2
Hordeum vulgare	BU970111_1	A	v2
Hordeum vulgare	TA43549_4513_3	A	v2
Hordeum vulgare	TA44310_4513_1	A	v2
Hordeum vulgare	TA40815_4513_1	A	v2
Hordeum vulgare	TA41215_4513_2	A	v2
Hordeum vulgare	TA47002_4513_2	A	v2
Hordeum vulgare	BF265504_2	A	v2
Hordeum vulgare	TA48040_4513_1	A	v2
Hordeum vulgare	TA39429_4513_3	B	v2
Hordeum vulgare	BF623499_3	C	v2
Hordeum vulgare	TA39303_4513_2	C	v2
Hordeum vulgare	TA39304_4513_2	C	v2
Hordeum vulgare	BF631054_3	D	v2
Hordeum vulgare	BQ662138_1	D	v2
Hordeum vulgare	TA41819_4513_2	D	v2
Hordeum vulgare	TA55610_4513_1	D	v2
Hordeum vulgare	TA40164_4513_3	D	v2
Hordeum vulgare	TA55989_4513_2	D	v2
Hordeum vulgare	TA41242_4513_1	E	v2
Hordeum vulgare	BE420598_1	E	v2
Hordeum vulgare	TA38477_4513_1	E	v2
Hordeum vulgare	TA48408_4513_1	E	v2

Hordeum vulgare	CV056083_3	F	v2
Hordeum vulgare	BE194756_3	F	v2
Hordeum vulgare	TA50001_4513_2	F	v2
Hordeum vulgare	CA591957_1	F	v2
Hordeum vulgare	TA45897_4513_1	F	v2
Hordeum vulgare	TA39995_4513_2	G	v2
Hordeum vulgare	AV836924_2	G	v2
Hordeum vulgare	TA49833_4513_2	G	v2
Hordeum vulgare	TA42154_4513_1	G	v2
Hordeum vulgare	BE060944_2	G	v2
Hordeum vulgare	TA44357_4513_2	G	v2
Hordeum vulgare	TA42755_4513_3	G	v2
Hordeum vulgare	TA43915_4513_2	H	v2
Hordeum vulgare	BM817340_2	H	v2
Hordeum vulgare	BF253604_2	I	v2
Hordeum vulgare	TA41272_4513_2	I	v2
Hordeum vulgare	TA48700_4513_3	I	v2
Hordeum vulgare	TA45344_4513_1	I	v2
Hordeum vulgare	CV063652_2	I	v2
Hordeum vulgare	BG369395_2	I	v2
Hordeum vulgare	BQ464812_3	I	v2
Hordeum vulgare	TA37782_4513_3	K	v2
Hordeum vulgare	BQ766888_2	S	v2
Hordeum vulgare	TA31297_4513_2	S	v2
Hordeum vulgare	TA40558_4513_3	S	v2
Hordeum vulgare	TA53781_4513_1	S	v2
Hordeum vulgare	TA34188_4513_3	S	v2
Hordeum vulgare	BI958110_1	S	v2
Hordeum vulgare	TA31294_4513_2	S	v2
Hordeum vulgare	BG415333_3	S	v2
Hordeum vulgare	TA35984_4513_1	S	v2
Hordeum vulgare	BE558681_2	S	v2
Hordeum vulgare	BG343063_2	S	v2
Hordeum vulgare	BG415877_3	S	v2
Hordeum vulgare	TA56942_4513_4	S	v2
Saccarum officinarum	CA255858_1	A	v2
Saccarum officinarum	CA168496_3	A	v2
Saccarum officinarum	CA189660_2	A	v2
Saccarum officinarum	CA233918_1	A	v2
Saccarum officinarum	CA095141_1	A	v2
Saccarum officinarum	CA109487_1	A	v2
Saccarum officinarum	CA254950_2	A	v2
Saccarum officinarum	TA42878_4547_3	A	v2
Saccarum officinarum	CA291484_3	A	v2
Saccarum officinarum	CA230985_1	A	v2
Saccarum officinarum	CA290952_3	A	v2
Saccarum officinarum	TA34020_4547_3	A	v2
Saccarum officinarum	TA40347_4547_1	A	v2
Saccarum officinarum	CA237816_4	A	v2
Saccarum officinarum	CA075795_5	A	v2
Saccarum officinarum	CA271379_1	A	v2
Saccarum officinarum	CA267374_3	B	v2
Saccarum officinarum	TA33202_4547_1	B	v2
Saccarum officinarum	TA31887_4547_1	C	v2
Saccarum officinarum	TA37832_4547_1	C	v2
Saccarum officinarum	CA228763_1	C	v2
Saccarum officinarum	CA239922_1	C	v2
Saccarum officinarum	CA285909_1	C	v2

Saccarum officinarum	CA228758_3	C	v2
Saccarum officinarum	CA166865_2	D	v2
Saccarum officinarum	CA276242_1	D	v2
Saccarum officinarum	TA36471_4547_3	D	v2
Saccarum officinarum	CA195716_3	D	v2
Saccarum officinarum	TA42776_4547_3	D	v2
Saccarum officinarum	TA33240_4547_1	D	v2
Saccarum officinarum	CA185286_2	D	v2
Saccarum officinarum	TA39406_4547_2	D	v2
Saccarum officinarum	CA093242_1	D	v2
Saccarum officinarum	TA49070_4547_1	E	v2
Saccarum officinarum	TA47532_4547_2	E	v2
Saccarum officinarum	CA109161_2	E	v2
Saccarum officinarum	CA130480_3	F	v2
Saccarum officinarum	CA139787_1	F	v2
Saccarum officinarum	CA234095_2	F	v2
Saccarum officinarum	CA073451_1	F	v2
Saccarum officinarum	CA150837_2	G	v2
Saccarum officinarum	TA39660_4547_1	G	v2
Saccarum officinarum	CA244895_1	G	v2
Saccarum officinarum	CA291622_2	G	v2
Saccarum officinarum	TA38482_4547_1	G	v2
Saccarum officinarum	CA260509_2	G	v2
Saccarum officinarum	CA298571_3	G	v2
Saccarum officinarum	TA38701_4547_3	G	v2
Saccarum officinarum	CA171228_1	G	v2
Saccarum officinarum	CA079641_2	G	v2
Saccarum officinarum	CA115460_2	H	v2
Saccarum officinarum	CA111537_2	H	v2
Saccarum officinarum	CA251578_1	I	v2
Saccarum officinarum	CA091164_1	I	v2
Saccarum officinarum	TA34763_4547_1	I	v2
Saccarum officinarum	CA299676_3	I	v2
Saccarum officinarum	CA104938_3	I	v2
Saccarum officinarum	TA34761_4547_3	I	v2
Saccarum officinarum	CA152996_3	I	v2
Saccarum officinarum	CA084077_2	I	v2
Saccarum officinarum	TA39843_4547_3	I	v2
Saccarum officinarum	CA281514_3	I	v2
Saccarum officinarum	TA35957_4547_1	K	v2
Saccarum officinarum	TA23286_4547_3	S	v2
Saccarum officinarum	CA253894_1	S	v2
Saccarum officinarum	CA261153_1	S	v2
Saccarum officinarum	CA298025_5	S	v2
Saccarum officinarum	CA285643_2	S	v2
Saccarum officinarum	TA23279_4547_2	S	v2
Saccarum officinarum	CA275565_2	S	v2
Saccarum officinarum	CA066728_3	S	v2
Saccarum officinarum	CA142570_2	S	v2
Saccarum officinarum	CA153252_1	S	v2
Saccarum officinarum	CA158302_1	S	v2
Saccarum officinarum	CA260549_2	S	v2
Saccarum officinarum	CA206226_1	S	v2
Saccarum officinarum	CA285017_4	S	v2
Saccarum officinarum	CA072374_2	S	v2
Saccarum officinarum	CA279874_1	S	v2
Saccarum officinarum	CA130717_1	S	v2
Saccarum officinarum	TA41955_4547_3	S	v2

Saccarum officinarum	TA28783_4547_5	S	v2
Saccarum officinarum	CA182708_5	S	v2
Saccarum officinarum	TA24586_4547_1	S	v2
Saccarum officinarum	CA078759_2	S	v2
Saccarum officinarum	CA080785_1	S	v2
Saccarum officinarum	CA253534_2	S	v2
Sorghum bicolor	TA37761_4558_1	A	v2
Sorghum bicolor	TA38660_4558_2	A	v2
Sorghum bicolor	TA26948_4558_1	A	v2
Sorghum bicolor	TA36148_4558_1	B	v2
Sorghum bicolor	TA32585_4558_2	B	v2
Sorghum bicolor	TA31772_4558_2	C	v2
Sorghum bicolor	TA27708_4558_3	C	v2
Sorghum bicolor	TA26252_4558_1	C	v2
Sorghum bicolor	TA23814_4558_1	C	v2
Sorghum bicolor	BE361313_1	D	v2
Sorghum bicolor	TA39304_4558_1	D	v2
Sorghum bicolor	TA22942_4558_1	D	v2
Sorghum bicolor	TA29009_4558_3	D	v2
Sorghum bicolor	TA32234_4558_1	D	v2
Sorghum bicolor	TA26387_4558_1	D	v2
Sorghum bicolor	TA22611_4558_3	D	v2
Sorghum bicolor	TA24293_4558_1	E	v2
Sorghum bicolor	BG947997_3	E	v2
Sorghum bicolor	TA30250_4558_2	E	v2
Sorghum bicolor	TA23717_4558_3	F	v2
Sorghum bicolor	TA28080_4558_2	G	v2
Sorghum bicolor	TA31175_4558_1	G	v2
Sorghum bicolor	TA27779_4558_3	G	v2
Sorghum bicolor	TA33129_4558_1	G	v2
Sorghum bicolor	TA29798_4558_1	G	v2
Sorghum bicolor	AW565182_1	G	v2
Sorghum bicolor	BE361650_3	H	v2
Sorghum bicolor	TA28676_4558_3	H	v2
Sorghum bicolor	CD463296_2	H	v2
Sorghum bicolor	TA31113_4558_1	K	v2
Sorghum bicolor	TA23432_4558_2	K	v2
Sorghum bicolor	TA23450_4558_2	S	v2
Sorghum bicolor	CN140419_1	S	v2
Sorghum bicolor	TA22038_4558_1	S	v2
Sorghum bicolor	TA21867_4558_1	S	v2
Sorghum bicolor	TA23370_4558_1	S	v2
Triticum aestivum	DR740223_3	A	v2
Triticum aestivum	TA80236_4565_1	A	v2
Triticum aestivum	CA625328_3	A	v2
Triticum aestivum	TA90746_4565_2	A	v2
Triticum aestivum	BE213424_2	A	v2
Triticum aestivum	BQ243523_2	A	v2
Triticum aestivum	TA104858_4565_1	A	v2
Triticum aestivum	TA92266_4565_1	A	v2
Triticum aestivum	BQ805403_3	A	v2
Triticum aestivum	BE400665_3	A	v2
Triticum aestivum	TA90745_4565_1	A	v2
Triticum aestivum	TA80237_4565_3	A	v2
Triticum aestivum	CK208293_1	A	v2
Triticum aestivum	TA110541_4565_3	A	v2
Triticum aestivum	CA702598_2	A	v2
Triticum aestivum	CA501646_1	A	v2

Triticum aestivum	BE516338_3	A	v2
Triticum aestivum	BJ240248_1	A	v2
Triticum aestivum	BJ263150_3	A	v2
Triticum aestivum	TA105268_4565_2	A	v2
Triticum aestivum	BU100519_3	A	v2
Triticum aestivum	TA79958_4565_1	A	v2
Triticum aestivum	CK217886_2	A	v2
Triticum aestivum	TA77309_4565_1	B	v2
Triticum aestivum	TA80673_4565_3	B	v2
Triticum aestivum	TA80671_4565_2	B	v2
Triticum aestivum	TA77308_4565_3	B	v2
Triticum aestivum	CJ673564_2	B	v2
Triticum aestivum	TA77249_4565_3	C	v2
Triticum aestivum	BM068654_3	C	v2
Triticum aestivum	TA58949_4565_3	C	v2
Triticum aestivum	CK213632_1	C	v2
Triticum aestivum	CA738001_1	C	v2
Triticum aestivum	TA77247_4565_2	C	v2
Triticum aestivum	TA95701_4565_1	C	v2
Triticum aestivum	CA652994_1	C	v2
Triticum aestivum	TA87219_4565_2	C	v2
Triticum aestivum	TA87220_4565_3	C	v2
Triticum aestivum	WHTHBP1BC1_3	D	v2
Triticum aestivum	DR741523_3	D	v2
Triticum aestivum	TA104387_4565_3	D	v2
Triticum aestivum	TA83965_4565_2	D	v2
Triticum aestivum	CA600392_2	D	v2
Triticum aestivum	TA61854_4565_3	D	v2
Triticum aestivum	TA92936_4565_2	D	v2
Triticum aestivum	TA70566_4565_2	D	v2
Triticum aestivum	DR739282_2	D	v2
Triticum aestivum	CF134232_3	D	v2
Triticum aestivum	TA74666_4565_2	E	v2
Triticum aestivum	CK163666_3	E	v2
Triticum aestivum	TA85462_4565_1	E	v2
Triticum aestivum	TA83436_4565_2	F	v2
Triticum aestivum	CA678659_1	F	v2
Triticum aestivum	TA105069_4565_3	F	v2
Triticum aestivum	BF473537_3	F	v2
Triticum aestivum	BJ316356_1	F	v2
Triticum aestivum	BE500054_3	F	v2
Triticum aestivum	CJ589087_6	G	v2
Triticum aestivum	CD871271_1	G	v2
Triticum aestivum	CA485656_3	G	v2
Triticum aestivum	CK163894_1	G	v2
Triticum aestivum	CV766489_3	G	v2
Triticum aestivum	TA70933_4565_3	G	v2
Triticum aestivum	TA87824_4565_2	G	v2
Triticum aestivum	CK166015_2	G	v2
Triticum aestivum	CV768933_3	H	v2
Triticum aestivum	TA89032_4565_1	H	v2
Triticum aestivum	CA725309_1	H	v2
Triticum aestivum	BU101264_3	I	v2
Triticum aestivum	TA88757_4565_1	I	v2
Triticum aestivum	TA111061_4565_2	I	v2
Triticum aestivum	TA79038_4565_1	I	v2
Triticum aestivum	CD868602_1	J	v2
Triticum aestivum	TA58175_4565_2	K	v2

Triticum aestivum	TA96488_4565_3	K	v2
Triticum aestivum	CA620381_2	S	v2
Triticum aestivum	DR736531_2	S	v2
Triticum aestivum	CA500176_2	S	v2
Triticum aestivum	CK205817_3	S	v2
Triticum aestivum	TA78527_4565_2	S	v2
Triticum aestivum	TA93350_4565_3	S	v2
Triticum aestivum	TA67477_4565_3	S	v2
Triticum aestivum	TA55615_4565_1	S	v2
Triticum aestivum	CD490860_3	S	v2
Triticum aestivum	BT009178_1	S	v2
Triticum aestivum	BQ246205_1	S	v2
Triticum aestivum	TA55715_4565_2	S	v2
Triticum aestivum	CV776972_1	S	v2
Triticum aestivum	TA103590_4565_2	S	v2
Triticum aestivum	TA93351_4565_2	S	v2
Zea mays	EE027071_2	A	v3
Zea mays	EE022333_3	A	v3
Zea mays	TA138324_4577_2	A	v3
Zea mays	TA122411_4577_3	A	v3
Zea mays	DR959319_1	A	v3
Zea mays	TA141798_4577_2	A	v3
Zea mays	TA156913_4577_1	A	v3
Zea mays	TA126948_4577_2	A	v3
Zea mays	TA122668_4577_1	A	v3
Zea mays	TA156772_4577_3	A	v3
Zea mays	TA139867_4577_1	A	v3
Zea mays	TA143482_4577_1	A	v3
Zea mays	TA97776_4577_2	A	v3
Zea mays	EB484417_1	A	v3
Zea mays	TA103855_4577_2	A	v3
Zea mays	TA125548_4577_3	A	v3
Zea mays	TA124243_4577_1	A	v3
Zea mays	TA131302_4577_2	A	v3
Zea mays	DR957951_2	A	v3
Zea mays	EE169440_2	A	v3
Zea mays	TA122667_4577_1	A	v3
Zea mays	TA116056_4577_1	B	v3
Zea mays	TA145569_4577_1	B	v3
Zea mays	DN217793_2	C	v3
Zea mays	TA117701_4577_2	C	v3
Zea mays	TA130201_4577_1	C	v3
Zea mays	BM501254_1	D	v3
Zea mays	TA125731_4577_1	D	v3
Zea mays	TA112686_4577_3	D	v3
Zea mays	TA110586_4577_2	D	v3
Zea mays	TA112689_4577_3	D	v3
Zea mays	TA133043_4577_1	D	v3
Zea mays	DR787705_3	D	v3
Zea mays	TA123354_4577_1	D	v3
Zea mays	TA139790_4577_2	D	v3
Zea mays	CK144343_2	D	v3
Zea mays	EE186071_1	D	v3
Zea mays	TA112687_4577_2	D	v3
Zea mays	TA129179_4577_3	D	v3
Zea mays	TA124545_4577_3	D	v3
Zea mays	TA110585_4577_1	D	v3
Zea mays	TA134232_4577_2	D	v3

Zea mays	TA121242_4577_1	D	v3
Zea mays	TA142240_4577_3	D	v3
Zea mays	TA133369_4577_3	E	v3
Zea mays	TA125482_4577_3	E	v3
Zea mays	TA149780_4577_3	E	v3
Zea mays	TA129075_4577_3	E	v3
Zea mays	TA134102_4577_2	E	v3
Zea mays	TA133368_4577_2	E	v3
Zea mays	TA134103_4577_1	E	v3
Zea mays	TA127033_4577_2	E	v3
Zea mays	TA111059_4577_1	F	v3
Zea mays	TA129620_4577_1	F	v3
Zea mays	TA101088_4577_2	F	v3
Zea mays	TA111061_4577_1	F	v3
Zea mays	CO451643_2	F	v3
Zea mays	DR792307_2	G	v3
Zea mays	TA110413_4577_2	G	v3
Zea mays	TA119351_4577_1	G	v3
Zea mays	TA130760_4577_3	G	v3
Zea mays	CF636457_6	G	v3
Zea mays	DR823628_3	G	v3
Zea mays	DR957801_2	G	v3
Zea mays	TA130761_4577_3	G	v3
Zea mays	CO442202_2	G	v3
Zea mays	AY685207_1	G	v3
Zea mays	TA155899_4577_1	G	v3
Zea mays	TA119352_4577_3	G	v3
Zea mays	TA119757_4577_4	G	v3
Zea mays	CK368868_1	H	v3
Zea mays	TA124686_4577_1	H	v3
Zea mays	TA153601_4577_2	H	v3
Zea mays	TA123976_4577_2	I	v3
Zea mays	TA118251_4577_1	I	v3
Zea mays	TA106179_4577_2	I	v3
Zea mays	TA137622_4577_2	I	v3
Zea mays	TA150185_4577_2	I	v3
Zea mays	TA122139_4577_1	I	v3
Zea mays	TA122138_4577_3	I	v3
Zea mays	TA117569_4577_3	I	v3
Zea mays	TA111661_4577_3	I	v3
Zea mays	TA115056_4577_3	I	v3
Zea mays	TA141207_4577_3	I	v3
Zea mays	TA106180_4577_3	I	v3
Zea mays	TA117567_4577_1	I	v3
Zea mays	TA134126_4577_2	I	v3
Zea mays	TA146671_4577_2	I	v3
Zea mays	TA119583_4577_2	I	v3
Zea mays	TA115055_4577_3	I	v3
Zea mays	TA118250_4577_2	I	v3
Zea mays	TA120717_4577_1	K	v3
Zea mays	TA124493_4577_5	S	v3
Zea mays	TA155378_4577_5	S	v3
Zea mays	TA123144_4577_3	S	v3
Zea mays	DN218501_3	S	v3
Zea mays	TA119413_4577_2	S	v3
Zea mays	TA112581_4577_1	S	v3
Zea mays	TA100631_4577_1	S	v3
Zea mays	TA100607_4577_1	S	v3

Zea mays	TA123142_4577_6	S	v3
Zea mays	TA156509_4577_3	S	v3
Zea mays	DN214099_1	S	v3
