# Supplementary material for: The Role of bZIP Transcription Factors in Green Plant Evolution: Adaptive Features Emerging from Four Founder Genes
Source: PLoS One. 2008 Aug 13;3(8):e2944. doi: 10.1371/journal.pone.0002944 (PMC2492810; doi:10.1371/journal.pone.0002944)
Supplement: Table S4 — Biological functions of genes in PoGOs. (0.02 MB PDF) [file pone.0002944.s024.pdf]

Biological functions of genes in PoGOs.

Group	PoGO	Genes	Function	Reference
A	1	DPBF4 (EEL), AREB3	Fine regulation of LEA (EEL), ABA responsiveness, drought and high salinity signaling	Bensmihen *et al*. (2005) - J Exp Bot.<br>Bensmihen *et al*. (2002)<br>Uno *et al*. (2000) - Proc Natl Acad Sci
	2	FD, FDP	Signaling of flowering time	Mathieu *et al.* (2007) - Curr Biol.<br>Abe et al. (2005) - Science.<br>Wellmer *et al*. (2006) - PLoS Genet.
	3	OSE2, GBF4, OSE2-like	Stress and ABA responsiveness, seed development	Choi *et al.* (2000) - J Biol Chem.<br>Cooper *et al.* (2003) - Proc Natl Acad Sci
	4	ABI5, DPBF2, DPBF1Ha	LEA gene ABA-dependent induction, seed oil biosynthesis, sugar signaling, leaf senescence	Nishimura *et al.* (2007) - Plant J<br>Wang *et al.* (2007) - Planta.<br>Bensmihen *et al.* (2004) - FEBS Lett.<br>Pourtau *et al.* (2004) - Planta.<br>Brocard-Gifford *et al.* (2003) - Plant Physiol
	5	TRAB1, ABF1-4, PvZIP6, PHI2, RIP55, ABI5Hv	ABA responsiveness, plant development, ABA signaling phosphate-dependent, fruit maturation, glucose signaling	Hobo *et al.* (1999) - Proc Natl Acad Sci<br>Davies and Robinson (2000) - Plant Physiol.<br>Kim *et al.* (2004) - Plant J.<br>Kang *et al*.(2002) - Plant Cell.<br>Kagaya *et al* (2002) - Plant Cell.<br>Sano and Nagata (2002) - Plant Cell Physiol.
B	1	AtbZIP28 and 17	Endoplasmatic reticulum responses	Liu *et al.* (2007) - Plant J.
C	1	REB, O2, OHP, Bzo2h3, CPRF2Pc, RISBZ1	Reserve protein accumulation, regulation of seed expression genes, energetic metabolism	Yamamoto *et al.* (2006) - Plant Physiol.<br>Kemper *et al.* (1999) - Plant Cell.<br>Baena-González *et al.* (2007) - Nature<br>Wellmer *et al* (1999) - J Biol Chem
	2	Bzo2h1	Cell death, basal defense, proline accumulation, seed storage protein gene activation	Weltmeier *et al.*(2006) - EMBO J.<br>Kaminaka *et al.* (2006) - EMBO J.<br>Lara *et al.* (2003) - J Biol Chem.
	3	RITA, Bzo2h2	Seed development, senescence, vascular tissue formation	Silveira *et al* (2007) - Plant Sci<br>Izawa *et al* (1994) - Plant Cell
	1	-	-	
	2	LG2Zm	Ligule formation, establishment of the leaf blade-sheath boundary	Harper and Freeling (1996) - Genetics

			sheath boundary	Walsh *et al.* (1998) - Genes Dev.
D	3	TGA3, TGA1, OBF4, STGA1Gm, TGA1.1Pv, MBF2St,PG13Nt,NITFLe	Phosphorus deficiency responsiveness, auxin response, marker of the end of cellular division, salicylic acid response, systemic resistence, jasmonic acid suppression, defense signalling in roots	Kesarwani *et al*. (2007) - Plant Physiol.
				Zhang *et al.* (2006) - Plant J.
				Johnson *et al*. (2003) - Plant Cell.
				Ndamukong *et al.* (2007) - Plant J.
				Foley *et al*. (2004) - FEBS Lett.
	4	Pan	Floral organ number determination	Hepworth *et al.* (2005) - Plant Cell
				Chuang *et al.* (1999) - Genes Dev.
	5	TGA2.1 and 2.2Pv, TGA2,5,6, TGA2.2	Pathogen, auxin and salicylic acid response	Thibaud-Nissen *et al*. (2006) - Plant J. 2006
				Kim and Delaney (2002) - Plant J.
				Kang and Klessing (2005) - Plant Mol Biol
				Zhang *et al*. (2003) - Plant Cell.
E	1	AtbZIP34 and 61	Plant development	Shen *et al.* (2007) - Biochem Biophys Res Commun.
F	1	-	-	
	2	-	-	
G	1	CPRF1Pc, ROM2, Osbz8, GBF1Zm, GBF1Bn, TAF2 and 3Nt	Light responsiveness, maturation and LEA gene repression, ABA regulation, Adh activation, photomorphogenesis	Sehnke *et al.* (2005) - Cell Res.
				Tamai *et al.* (2002) - Plant Cell Physiol
				Feldbrugge et al. (1994) - Plant Cell.
				Chern *et al.* (1996) - Plant Cell. (1)
	2	ROM1	Maturation gene repression	Chern *et al.* (1996) - Plant Cell. (2)
	3	CPRF4aPc	Light responsiveness	Sprenger-Haussels and Weisshaar (2000) - Plant J
				Kircher *et al.* (1998) - Mol Gen Genet
	4	EmBP1aZm, HALF1Ta, Embp1Ta	Reserve protein control	Okanami *et al.* (1996) - Genes Cells.
				Eckardt *et al.* (1998) - Plant Mol Biol.
				Hill *et al.* (1996) - J Biol Chem.
H	1	Hy5Like, THY5	Photomorphogenesis, light mediated gene expression	Holm *et al.* (2002) - Genes Dev.
	2	Hy5, THY5Le	Photomorphogenesis, light mediated gene expression, anthocyanin metabolism	Vandenbussche *et al*. (2007) - Plant J.
				Shin *et al.* (2007) - Plant J.
				Lee *et al.* (2007) - Plant Cell.
				Jonassen et al. (2007) - Planta
	1	VIP1, RSGNt, PKSF1	Cell elongation, VirE protein interaction, T-DNA integration, control of gibberellin accumulation	Anand *et al.* (2007) - Plant Cell.
				Li *et al.* (2005) - Proc Natl Acad Sci USA
				Fukazawa *et al*. (2000) - Plant Cell
		RF2a, PosF21	Vascular development, pathogen response	Dai *et al.* (2003) - J Biol Chem

I	2			Zhu *et al.* (2002) - Plant Cell.
				Yin *et al.* (1997) - EMBO J.
	3	RF2b	Pathogen response	Liu *et al.* (2007) - Biochem J
				Dai *et al.* (2004) - Proc Natl Acad Sci USA
	4	VSF1Le	Control of xylem gene expression	Torres-Schumann *et al.* (1996) - Plant J
				Ringli and Keller (1998) - Plant Mol Biol.
	PoGPI1	UNE4	Female gametophyte development	Pagnussat *et al.* (2005) - Development
J	1	-	-	
K	1	AtbZIP60	ER stress	Iwata *et al.* (2005) - Proc Natl Acad Sci
L	1	-	-	
	2	-	-	
S	S1	AAK01953Pa, AAK25822Pv	Water deficit response	Rodriguez-Uribe and O'Connell (2006) - Journal of Experimental Botany
	SE1	-	-	
	SM1	-	-	
	SE2	PPI1Cc,BZI-2Nt, CPRF7Pc, ATB2, GBF5	Light response, hypoosmolarity response, pathogen defence	Satoh *et al.* (2004) - Plant Cell Physiology
				Wiese *et al*. (2005) - Biochemical Society Transactions
				Rook *et al.* (1998) - The Plant Journal
				Rook et al. (1998) - Plant Molecular Biology
				Strathmann *et al.* (2001) - The Plant Journal
				Rügner *et al.* (2001) - Mol Genet Genomics
				Lee *et al.* (2002) - Mol Plant Microbe Interact
	SM2	mLIP15, OBF1Zm, OsbZIP86, OsbZIP87	Cold response and signaling	Singh *et al.* (1990) - The Plant Cell
				Shimizu *et al.* (2005) - Plant Cell Physiol
				Aguan *et al.* (1993) - Mol Gen Genet.
				Kusano *et al.* (1995) - Mol Gen Genet.
	SE3	TBZFNt, BZI-3Nt, AAD55394Le, TBZ17, CAA74022, CAA774023, CpZIP1, CpZIP2, ABZ1, BZI-4, CPRF6, AtbZIP53	Hypoosmolarity response, light response, senescence, histone regulation, drought response, ABA signaling, anaerobic response	Satoh *et al.* (2004) - Plant Cell Physiology
				Weltmeier *et al.* (2006) - The EMBO Journal
				Rügner *et al.* (2001) - Mol Genet Genomics
				Yang *et al.* (2001) - Plant Physiology
				Martinez-Garcia *et al.* (1998) - The Plant Journal
				Ditzer and Bartels (2006) - Plant Mol Biol
				Sell and Hehl (2004) - Eur. J. Biochem.
				Stankovic *et al. (2000) - Planta*
