# Supplementary material for: The Role of bZIP Transcription Factors in Green Plant Evolution: Adaptive Features Emerging from Four Founder Genes
Source: PLoS One. 2008 Aug 13;3(8):e2944. doi: 10.1371/journal.pone.0002944 (PMC2492810; doi:10.1371/journal.pone.0002944)
Supplement: Table S7 — Gene pairs resulting from segmental duplications of the Arabidopsis genome. (0.03 MB DOC) [file pone.0002944.s027.doc]

| **Table S5**. Gene pairs resulting from segmental duplications of the Arabidopsis genome. |
| --- |
| Group A |
| - DPBF4 and AREB3 - AtbZIP13 and GBF4 - ABF4 and ABF1 - ABF2 and ABF1 - AtbZIP14 and AtbZIP17 |
| Group B |
| - AtbZIP49 and AtbZIP17 - AtbZIP49 and AtbZIP28 |
| Group D |
| - TGA1 and OBF4 - AtbZIP50 and TGA3 - TGA3 and TGA1 - PAN and AtbZIP21 - TGA6 and TGA2 |
| Group E |
| - AtbZIP61 and AtbZIP34 |
| Group F |
| - AtbZIP23 and AtbZIP19 |
| Group G |
| - GBF2 and GBF3 - AtbZIP16 and AtbZIP68 |
| Group I |
| - AtbZIP18 and VP1 - AtbZIP18 and AtbZIP52 - POSF21 and AtbZIP69 - AtbZIP30 and AtbZIP29 |
| Group S |
| - GBF5 and Atb2 - GBF5 and AtbZIP44 - AtbZIP6 and AtbZIP7 - AtbZIP58 and AtbZIP48 - AtbZIP42 and AtbZIP43 |
