# Supplementary material for: The Role of bZIP Transcription Factors in Green Plant Evolution: Adaptive Features Emerging from Four Founder Genes
Source: PLoS One. 2008 Aug 13;3(8):e2944. doi: 10.1371/journal.pone.0002944 (PMC2492810; doi:10.1371/journal.pone.0002944)
Supplement: Dataset S3 — MPSS Expression data for bZIP genes from rice. (0.02 MB PDF) [file pone.0002944.s030.pdf]

AGI	POGO	9LA	9LB	9LC	9LD	9ME	9RO	9RR	FLA	FLB	FLC	FLD	FME	FRO	FRR	MC00	MC24	MR03	MR06	MR12	MR24	MR48	MS03	MS06	MS12	MS24	MS48
Os01g59760	A1	0	0	0	0	0	0	0	0	13	0	0	5	0	0	0	0	0	0	0	0	0	0	0	29	0	3
Os03g20650	A1	0	0	0	0	0	0	0	0	0	14	14	0	0	0	0	0	0	0	0	0	0	0	0	0	0	0
Os05g41070	A1	27	72	40	18	57	0	150	111	34	18	18	96	143	194	79	8	117	19	26	39	2	70	36	2	55	54
Os02g58670	A2	0	25	0	0	0	5	25	0	0	8	8	4	0	0	11	0	37	0	5	0	10	24	0	0	0	89
Os06g50600	A2	0	0	0	0	2	17	52	0	0	0	0	22	1	0	17	72	0	9	2	0	7	0	0	2	6	2
Os08g43600	A2	0	0	0	0	0	0	0	0	0	0	0	0	8	6	0	0	0	0	0	0	0	0	0	0	0	0
Os09g36910	A2	0	0	0	0	0	0	0	0	0	0	0	0	0	0	0	0	0	0	0	0	0	0	0	0	0	0
Os01g64730	A3	3	10	0	0	0	0	10	0	26	10	10	6	27	0	13	47	97	4	38	9	34	38	0	14	7	85
Os05g36160	A3	0	0	0	0	0	0	0	0	0	0	0	3	0	0	0	2	1	0	0	0	0	0	0	10	0	1
Os02g52780	A5	35	141	56	101	312	83	63	150	283	49	49	254	299	405	22	0	0	2	50	2	6	5	0	32	7	0
Os06g10880	A5	0	0	0	0	0	0	0	0	17	0	0	0	0	0	0	0	0	0	0	0	1	0	0	29	0	0
Os08g36790	A5	14	14	90	55	43	0	67	2	89	49	49	46	14	12	47	65	77	72	72	27	7	13	40	117	8	23
Os09g28310	A5	160	33	2	5	61	46	35	0	87	10	10	0	25	66	55	16	0	0	0	5	19	19	7	29	0	7
Os05g34050	B1	0	6	0	0	0	1	0	0	0	0	0	0	0	0	0	0	0	2	5	0	0	0	0	0	2	39
Os07g44950	B1	0	0	0	0	0	0	0	0	0	6	6	0	22	28	7	67	9	0	0	0	19	4	0	0	0	6
Os03g58250	C1	34	2	5	0	0	4	31	0	51	0	0	0	22	27	84	90	11	18	44	138	20	14	90	12	65	65
Os07g08420	C1	0	0	0	0	0	0	0	0	0	0	0	0	0	0	0	4	0	0	0	0	0	0	0	0	0	0
Os02g07840	C3	0	2	0	0	0	291	265	0	4	0	0	117	135	42	234	150	132	10	174	24	0	45	79	108	246	80
Os02g16680	C3	0	28	20	0	94	108	186	0	0	82	82	109	168	102	42	6	26	40	46	14	84	56	0	34	38	64
Os06g45140	C3	0	0	0	0	11	39	32	0	6	0	0	0	0	0	0	206	4	65	245	67	29	0	120	280	0	7
Os02g22280	D1	0	0	0	0	0	59	0	0	64	0	0	0	8	9	76	0	0	0	0	0	0	0	35	0	0	0
Os06g41100	D1	0	0	0	0	0	0	0	0	0	0	0	0	0	0	1	0	0	0	0	0	1	0	0	0	56	0
Os09g10840	D1	0	0	0	19	0	13	0	0	0	0	0	0	8	13	1	11	0	8	3	1	0	0	0	0	0	0
Os09g31390	D1	0	4	0	0	0	99	7	0	0	0	0	0	0	0	0	0	0	0	0	4	0	0	0	0	4	0
Os01g64020	D2	0	4	0	0	6	1	223	0	0	1	1	25	20	11	8	21	4	20	0	4	10	0	9	10	17	13
Os11g05480	D2	0	0	0	0	0	21	22	0	0	0	0	0	19	0	0	0	3	6	0	18	0	0	0	0	2	0
Os12g05680	D2	0	0	6	55	2	62	552	0	0	0	0	0	34	31	10	70	21	210	35	52	22	4	58	10	58	29
Os04g54474	D3	19	14	0	0	79	0	44	0	2	0	0	0	73	142	0	0	0	0	33	5	18	15	0	30	33	4
Os08g07970	D3	7	43	10	50	1414	346	421	125	65	27	27	329	1111	1305	65	44	14	0	7	0	93	75	9	2	72	53
Os01g17260	D5	41	8	92	83	3	63	110	4	131	148	148	16	23	10	101	53	186	42	22	2	28	40	6	38	29	20
Os01g59350	D5	0	0	0	0	0	0	0	0	0	0	0	0	0	0	33	0	0	7	8	0	33	6	0	15	0	0
Os03g20310	D5	65	54	0	0	16	83	157	0	27	27	27	25	22	9	57	228	83	89	97	190	51	49	128	79	23	68
Os07g48820	D5	429	636	535	719	397	282	656	431	575	170	170	250	437	425	214	369	171	176	281	32	187	140	769	410	345	273
Os01g11350	E1	0	0	0	0	0	0	0	0	0	0	0	0	0	0	1	0	0	0	0	0	0	0	0	0	0	0
Os01g55150	E1	0	0	0	0	0	145	0	0	0	0	0	1	33	9	0	0	0	0	0	0	0	0	0	0	0	1
Os02g14910	E1	0	0	0	0	0	0	0	0	0	0	0	0	0	0	0	0	0	0	0	0	0	0	0	0	0	0
Os01g46970	G1	0	0	0	0	0	12	0	0	0	0	0	0	0	0	0	0	0	0	0	0	0	0	0	0	0	2
Os05g49420	G1	0	0	0	0	0	0	24	0	10	1	1	0	0	0	5	20	31	0	0	40	0	1	238	0	179	3
Os02g03580	G3	39	33	24	15	20	87	11	0	60	72	72	23	6	3	24	98	21	60	70	121	29	33	58	116	43	39
Os12g13170	G3	170	86	130	22	0	1	75	0	168	34	34	39	0	0	67	164	261	111	55	163	28	112	70	138	33	76
Os01g07880	H1	0	0	0	0	0	0	0	0	0	7	7	0	0	0	0	0	10	15	15	0	23	125	0	14	0	14
Os06g39960	H1	0	0	42	0	0	0	0	0	0	0	0	0	0	0	0	0	0	53	0	0	0	0	0	0	0	0
Os11g06170	I1	0	0	0	0	0	0	0	0	0	0	0	10	0	0	0	1	4	32	0	2	0	33	0	0	0	9
Os12g06520	I1	336	246	141	109	5	139	155	0	102	0	0	24	29	41	0	0	14	0	150	0	0	31	31	29	25	0
Os04g41820	I2	0	0	0	0	0	0	0	0	0	0	0	0	1	4	1	0	0	0	0	0	0	0	3	12	0	0
Os09g34060	I2	1	0	5	5	0	0	0	0	9	0	0	0	0	0	34	6	8	0	0	43	35	26	10	0	2	34
Os03g21800	I3	0	0	0	0	0	44	23	0	0	0	0	4	9	5	49	0	6	0	182	72	57	19	179	66	35	32
Os07g48180	I3	0	0	0	0	0	0	0	0	0	0	0	0	0	0	0	0	0	0	0	0	0	0	0	0	0	0
Os03g03550	I4	0	0	4	14	0	0	4	0	8	0	0	22	0	0	0	0	0	0	0	0	0	0	0	0	0	0
Os04g10260	I4	0	0	0	0	0	0	0	0	0	0	0	0	0	0	0	0	0	0	0	0	0	0	0	0	0	0
Os08g43090	I4	0	0	0	0	0	0	0	10	82	1	1	8	0	0	169	45	176	18	21	132	88	109	108	44	88	42
Os09g34880	I4	0	0	0	0	0	0	0	0	0	0	0	0	0	0	0	0	0	0	0	0	0	0	0	0	0	0
Os10g38820	I4	0	0	0	0	0	0	0	0	0	0	0	0	0	0	0	0	0	0	0	0	0	0	0	0	0	0
Os11g05640	J1	0	0	0	0	0	0	0	0	0	0	0	0	0	0	0	0	0	0	0	0	0	0	0	0	0	0
Os12g06010	J1	18	137	109	103	53	101	209	10	187	176	176	50	61	57	20	108	75	97	24	42	138	117	124	109	50	79
Os11g11100	L2	0	0	0	0	0	53	11	0	2	1	1	0	0	0	96	8	7	69	38	3	106	12	25	42	93	18
Os12g09270	L2	0	0	0	0	0	0	0	0	0	76	76	0	0	0	0	0	0	4	1	4	1	0	0	2	0	12
Os01g36220	S1	0	48	0	0	0	35	36	0	11	0	0	0	31	16	0	0	42	7	71	11	28	81	12	31	41	30
Os02g03960	S1	221	491	447	1000	2191	167	154	1246	203	337	337	1251	796	931	148	18	128	156	79	2	94	109	0	180	232	105
Os02g49560	S1	0	0	0	0	0	0	0	0	0	0	0	0	0	0	0	0	0	0	0	0	0	0	0	0	0	0
Os03g19370	S1	0	0	2	0	0	11	0	0	0	0	0	0	0	0	0	0	0	0	0	0	0	0	0	0	0	0
Os03g47200	S1	0	0	0	0	0	0	0	0	0	0	0	0	0	0	0	0	0	0	0	0	0	0	0	0	0	0
Os05g03860	S1	4098	2386	2186	1799	11017	6726	11073	8451	2177	1000	1000	10207	17669	22254	1495	3008	1602	2086	4376	2898	992	1452	4226	3126	2264	2625
Os07g03220	S1	0	0	0	0	0	0	0	0	0	6	6	0	0	0	1	0	3	29	4	0	0	0	0	0	9	0
Os08g26880	S1	96	123	127	91	143	0	23	0	92	58	58	40	48	46	1	0	0	0	0	0	0	4	0	2	0	1
Os08g38020	S1	0	0	0	0	0	0	0	0	0	0	0	0	0	0	0	0	0	0	0	12	0	0	0	0	0	0
Os09g13570	S1	3	0	0	18	0	0	0	0	14	0	0	0	0	0	0	0	0	0	0	0	29	0	0	0	0	0
Os09g29820	S1	0	0	0	0	0	0	0	0	0	0	0	0	0	0	0	0	0	0	0	0	0	0	0	0	0	0
Os12g37410	S1	252	358	361	442	667	593	537	1606	503	284	284	532	1045	1383	262	860	605	352	850	873	195	343	899	880	1367	760
Os12g43790	S1	0	0	0	0	0	0	0	0	0	0	0	0	0	0	0	0	0	0	0	0	0	0	0	0	0	0

MS96	NCA	NLA	NLB	NLC	NLD	NME	NOS	NPO	NR2	PLC	PLW	PSC	PSI	PSL	PSN	PSY	XC00	XC06	XC24	XR03	XR06	XR12	XR24	XR48	XS03	XS06	XS12
0	11	5	24	0	0	4	8	0	0	2	2	96	0	0	0	2	0	0	0	0	0	0	2	0	0	8	0
0	0	0	0	0	0	1	0	0	0	0	0	0	0	0	0	0	0	0	0	0	0	0	0	0	0	0	0
112	40	68	10	64	127	66	161	63	0	31	54	18	46	16	52	34	77	0	86	97	6	32	46	126	87	50	25
0	14	0	0	0	9	23	8	0	0	22	0	0	0	0	0	0	4	0	0	0	0	17	101	23	144	23	16
0	28	0	0	0	2	0	50	0	0	0	2	0	14	0	0	0	2	0	0	3	0	0	14	0	0	0	27
0	0	0	0	0	0	0	0	0	0	0	0	0	0	0	0	0	0	0	0	0	0	0	0	0	0	0	0
0	0	0	0	0	0	1	0	0	0	0	0	0	0	0	0	0	0	0	0	0	0	0	0	0	0	0	0
2	0	4	3	0	0	23	0	23	0	0	6	52	0	13	0	0	0	0	0	54	0	0	24	56	0	32	0
0	5	0	0	0	0	6	37	5	0	0	0	65	23	0	39	21	0	0	0	0	0	0	0	5	19	1	1
47	15	29	103	16	40	175	92	102	0	5	40	0	0	2	0	0	9	87	10	60	74	80	11	37	6	81	212
0	90	5	22	0	0	7	0	0	0	0	2	19	25	30	0	0	2	0	0	0	0	0	30	0	0	10	0
117	29	7	38	1	40	62	138	14	0	9	87	2	7	8	23	13	82	37	3	30	39	28	0	40	204	105	6
22	22	11	119	32	30	27	0	11	0	497	255	0	58	104	2	0	68	69	111	45	107	200	0	13	28	144	284
0	0	0	0	0	0	0	2	5	0	0	4	0	2	0	27	15	0	0	0	0	0	0	0	0	0	12	0
0	0	0	0	0	0	0	119	45	0	0	10	0	9	49	12	0	0	0	0	0	0	0	0	0	0	0	0
0	7	0	27	22	20	0	44	3	0	0	4	1	2	20	7	2	4	3	0	37	0	46	0	56	0	0	11
0	0	0	0	0	0	0	0	0	0	0	0	1279	677	1462	1801	1429	0	0	0	0	0	0	0	7	0	0	0
0	0	0	0	0	0	330	0	12	0	0	0	0	0	0	2	0	27	18	0	0	0	135	48	0	0	63	0
0	64	0	0	0	0	92	2	24	0	0	0	136	0	6	0	0	0	0	0	34	54	30	0	24	0	56	110
0	77	0	0	0	0	5	0	3	0	0	0	179	25	61	12	255	0	0	0	0	0	0	0	0	31	0	77
0	28	0	0	0	0	11	0	0	0	0	0	0	0	0	0	0	0	0	0	45	55	0	0	40	14	15	0
0	0	0	0	0	0	0	0	0	0	0	0	0	0	0	0	0	0	0	0	0	0	0	0	0	0	0	0
2	0	0	0	0	0	0	0	0	0	0	0	0	3	11	0	2	7	25	0	0	0	0	0	0	0	7	0
0	0	0	0	0	0	0	0	1	0	0	0	18	108	36	11	31	0	0	0	0	0	0	0	0	3	0	0
0	0	0	0	0	0	0	0	0	0	0	0	0	0	0	0	0	0	0	0	0	0	0	0	28	0	0	1
0	21	0	0	0	0	0	2	39	0	0	0	0	0	2	0	0	0	23	0	0	0	0	0	0	0	0	0
4	118	0	0	3	10	0	0	12	0	0	8	0	0	0	0	0	6	36	7	0	0	0	0	0	29	19	0
8	0	37	28	13	0	101	12	0	0	2	21	0	0	0	0	0	35	0	0	0	57	23	0	31	17	0	0
150	16	168	208	0	0	408	3	26	0	4	102	0	0	2	0	0	111	150	100	13	6	84	173	10	12	36	112
340	74	0	77	32	136	52	241	1	0	19	89	83	9	97	91	104	24	33	0	47	8	37	0	0	47	174	24
0	95	0	0	0	0	1	40	0	0	0	0	0	0	0	0	0	0	9	0	3	0	0	0	68	0	68	0
83	25	11	3	22	31	58	31	0	0	16	9	6	2	56	0	12	18	60	111	10	3	6	55	0	107	86	53
54	32	401	314	296	432	158	26	29	0	41	216	3	0	76	69	35	472	107	414	154	171	154	119	212	213	163	205
0	0	0	0	0	0	0	3	0	0	0	0	0	0	0	0	0	0	0	0	0	0	0	0	0	0	0	0
0	0	0	0	0	0	0	27	0	0	0	0	0	0	0	12	0	16	0	0	0	0	0	0	0	0	0	0
0	0	0	0	0	0	0	0	0	0	0	0	0	0	0	0	0	0	0	0	0	0	0	0	0	0	0	0
0	37	0	0	0	0	0	0	14	0	0	0	0	0	0	5	0	0	0	0	0	0	0	0	0	0	0	0
0	0	14	3	0	0	0	0	0	0	0	0	0	0	0	31	32	0	0	2	3	3	0	0	0	1	1	0
37	26	15	64	0	26	10	161	75	0	3	0	58	0	3	0	0	37	7	19	98	9	35	34	0	61	10	47
87	58	29	209	115	93	15	181	69	0	35	102	5	89	23	37	2	72	56	74	43	79	48	100	13	81	89	125
0	106	0	12	0	9	32	69	2	0	0	8	3	8	53	0	0	5	6	0	35	65	32	0	2	51	6	89
0	0	0	0	67	0	0	3	0	0	0	0	0	0	0	0	0	4	0	0	0	0	25	0	0	0	0	11
0	0	0	0	0	0	0	0	0	0	0	0	86	0	4	0	0	0	0	0	0	0	0	0	0	0	0	0
0	24	13	191	82	156	4	62	6	0	0	38	0	14	0	6	0	26	4	0	118	25	0	61	17	108	124	18
0	70	0	0	0	2	20	112	7	0	0	0	10	0	5	3	3	0	0	0	0	0	0	0	0	0	0	0
48	20	0	4	0	8	0	0	63	0	0	30	50	20	26	4	41	32	69	9	12	17	46	14	36	29	17	23
0	35	0	0	0	0	1	16	193	0	0	0	101	0	43	6	210	0	0	0	0	3	0	0	0	12	15	0
0	0	0	0	0	0	0	0	0	0	0	0	0	0	0	0	0	0	0	0	0	0	0	0	0	0	0	0
101	79	0	0	0	0	19	2	81	0	0	0	0	105	44	69	13	0	0	0	0	0	0	0	0	0	0	6
0	0	0	0	0	0	0	0	0	0	0	0	5	29	188	54	6	0	0	0	0	0	0	0	0	0	0	0
186	91	35	131	30	93	116	164	2	0	53	54	189	288	99	193	74	50	67	0	90	68	21	122	36	105	59	59
0	0	0	0	0	0	0	0	0	0	0	0	73	18	77	0	2	0	0	0	0	0	0	0	0	0	0	0
0	0	0	0	0	0	2	0	0	0	0	0	0	0	0	0	0	0	0	0	0	0	0	0	0	0	0	0
0	45	0	0	0	0	0	0	0	0	0	2	0	0	0	0	0	0	0	0	0	0	0	0	0	0	0	0
38	126	0	52	94	38	50	35	0	0	42	9	325	462	419	307	155	72	232	12	121	73	3	209	57	84	74	20
7	3	0	0	0	28	0	29	2	0	12	19	12	20	8	0	0	0	14	7	6	3	14	22	17	5	19	131
40	51	0	0	0	0	0	0	0	0	0	0	0	0	0	0	0	0	4	0	0	0	0	0	22	0	3	0
20	0	78	69	16	0	0	0	0	0	0	8	0	0	0	0	0	292	19	0	101	73	165	0	23	73	41	229
254	0	314	160	424	211	941	893	459	0	16	15	0	178	46	4	17	457	236	613	194	140	220	67	293	304	299	188
0	0	0	0	0	0	5	0	0	0	0	0	0	0	0	0	0	0	44	0	0	0	0	0	0	0	0	0
0	0	0	0	0	0	0	0	0	0	0	0	0	0	0	0	0	0	0	0	0	0	0	0	0	0	0	0
0	0	0	0	0	0	0	0	0	0	5	0	0	0	0	0	0	0	0	0	0	0	0	0	0	0	0	0
1349	1077	4191	2812	1773	1800	5025	499	253	0	378	1162	329	645	461	694	348	2000	2061	2548	1259	1122	1739	1299	1114	1590	934	1410
0	0	19	0	0	0	0	0	0	0	0	0	0	0	0	0	0	0	0	0	8	2	0	0	0	15	2	0
0	4	63	24	0	0	13	5	10	0	0	6	0	3	7	10	0	26	9	44	12	7	45	4	5	124	15	0
2	0	0	0	0	0	0	0	0	0	0	0	0	0	0	0	0	0	0	0	0	0	0	0	0	0	0	0
0	0	0	3	0	0	0	0	0	0	0	27	0	40	6	0	0	0	19	0	0	0	0	4	0	12	6	69
0	0	0	0	0	0	0	0	0	0	0	0	0	0	0	0	0	0	0	0	0	0	0	0	0	0	0	0
345	42	415	736	1071	681	394	397	8	0	95	170	39	123	275	125	15	806	239	845	199	402	323	277	773	445	245	198
0	0	0	0	0	0	0	0	0	0	3	0	0	0	0	0	0	0	0	0	0	9	0	0	0	0	0	0

XS24	XS48	NRA	NRB	NSL	NSR	NST	NYL	NYR	PLA
7	3	15	0	0	14	0	0	0	0
0	0	1	0	0	0	9	0	8	0
18	42	191	335	74	174	28	27	31	36
0	0	0	0	20	0	0	17	0	0
16	9	3	25	19	9	0	2	42	0
0	0	1	0	0	0	7	0	0	0
0	0	0	0	0	0	0	0	0	0
36	16	24	0	168	13	0	104	0	0
0	0	0	0	1	0	0	3	16	0
79	83	105	122	126	78	81	15	59	47
0	9	4	0	0	3	22	0	0	5
12	11	20	57	26	11	35	34	5	9
12	93	54	0	181	24	70	54	0	379
2	0	6	0	0	3	0	0	0	4
0	0	4	49	27	0	54	9	0	0
5	2	0	6	11	11	20	26	0	0
0	0	0	42	12	18	0	0	0	0
15	105	330	312	209	325	34	111	210	4
26	6	246	44	19	216	48	24	376	0
0	2	2	31	0	1	0	57	0	0
0	0	8	85	0	0	42	0	0	0
0	0	0	0	0	0	0	0	6	0
33	0	88	67	17	12	0	5	102	0
0	0	24	82	0	0	0	0	18	0
17	0	129	200	0	175	0	0	24	0
0	0	22	7	1	130	0	0	28	0
0	0	135	288	1	295	0	0	193	2
6	0	147	126	0	551	62	0	201	0
3	57	1079	136	86	782	76	35	888	25
35	29	10	8	106	0	0	30	8	78
0	8	0	30	57	0	0	26	0	0
26	9	45	73	69	73	11	74	142	0
159	287	494	168	130	116	20	249	446	45
0	0	0	0	3	12	56	0	0	4
0	0	23	0	0	18	175	0	28	0
0	0	0	0	0	0	25	0	0	0
0	0	2	0	3	0	0	0	0	0
0	0	4	0	0	0	19	0	0	0
52	14	29	7	49	7	28	59	0	7
42	67	87	248	186	52	16	112	33	58
28	5	0	5	20	2	23	23	0	0
2	0	0	0	0	0	0	0	0	0
2	0	0	4	9	28	0	1	0	2
51	65	0	139	68	91	78	41	154	32
2	0	0	0	0	0	2	0	0	0
10	104	8	0	16	0	60	40	0	22
0	0	32	5	0	26	38	0	6	0
0	0	13	0	0	0	0	0	30	0
0	0	0	0	0	0	0	0	2	0
0	0	0	0	0	0	0	0	0	0
78	71	45	56	138	55	26	92	79	18
0	0	0	0	0	0	0	0	0	0
0	0	0	0	0	0	0	0	0	0
0	0	0	0	0	0	0	0	0	0
162	68	60	70	55	38	0	63	100	42
26	8	3	17	40	7	31	57	0	27
29	0	0	0	0	2	0	0	0	0
0	125	15	31	96	19	0	197	14	0
97	159	573	29	137	121	18	381	156	72
0	0	0	0	0	0	0	0	0	0
0	0	11	0	0	2	0	0	3	0
0	0	16	0	0	0	0	0	8	0
1163	2229	11725	8603	727	14243	4591	1437	8813	195
0	0	0	0	0	0	0	0	0	0
2	43	39	42	10	123	1	25	80	27
0	0	0	0	1	9	0	0	0	0
29	14	10	0	4	0	0	5	0	0
0	0	16	0	0	0	0	0	8	0
537	628	1214	336	298	329	283	217	191	132
0	0	2	0	0	0	0	0	0	0
