# Supplementary material for: The Role of bZIP Transcription Factors in Green Plant Evolution: Adaptive Features Emerging from Four Founder Genes
Source: PLoS One. 2008 Aug 13;3(8):e2944. doi: 10.1371/journal.pone.0002944 (PMC2492810; doi:10.1371/journal.pone.0002944)
Supplement: Dataset S4 — MPSS Expression data for bZIP genes from Arabidopsis. (0.01 MB PDF) [file pone.0002944.s031.pdf]

AGI	PoGo	CAF	INF	LEF	ROF	SIF	AP1	AP3	AGM	INS	ROS	SAP	S04	S52	LES	GSE	CAS	SIS
**At3g56850**	A1	22	31	34	35	15	7	15	14	30	10	9	0	9	3	0	4	1
**At2g41070**	A1	229	4	8	26	0	2	0	0	2	30	0	0	0	3	0	0	0
**At5g42910**	A2	1	0	0	0	0	0	0	0	0	0	0	0	0	0	0	0	0
**At4g35900**	A2	16	0	7	0	0	8	0	0	2	0	3	0	1	8	0	0	0
**At5g44080**	A3	8	10	3	0	0	13	29	20	20	12	59	3	19	20	6	23	17
**At1g03970**	A3	12	0	2	0	0	15	11	0	6	15	58	0	0	80	12	83	24
**At3g19290**	A5	46	13	28	26	29	0	26	0	7	0	0	0	20	43	2	0	1
**At1g45249**	A5	0	4	13	31	13	2	8	13	9	2	7	0	3	0	0	0	0
**At1g49720**	A5	4	0	23	0	13	27	41	45	11	15	21	187	52	12	15	15	2
**At4g34000**	A5	110	14	31	139	23	8	2	1	7	10	4	0	0	6	15	18	0
**At2g40950**	B1	38	43	9	13	11	12	20	5	8	3	2	0	3	4	0	40	10
**At3g10800**	B1	59	18	9	48	48	20	20	12	31	58	22	6	2	16	7	92	23
**At3g56660**	B1	0	0	0	0	0	0	0	0	3	0	8	0	0	0	0	0	0
**At4g02640**	C2	125	109	65	85	117	10	22	15	4	48	8	2	0	35	4	33	56
**At3g54620**	C2	46	19	25	0	5	80	69	131	46	99	87	48	64	110	31	433	122
**At5g65210**	D3	0	0	0	0	0	0	0	1	0	209	0	0	0	0	0	35	0
**At5g10030**	D3	12	0	21	44	0	0	1	0	0	7	0	0	0	15	0	0	0
**At1g22070**	D3	49	15	15	37	6	12	36	26	12	67	29	21	117	21	9	181	26
**At1g77920**	D3	4	0	0	3	0	0	0	0	0	0	0	0	0	0	0	3	0
**At3g12250**	D5	18	9	19	57	7	6	19	9	7	15	6	40	5	3	1	13	9
**At5g06960**	D5	0	0	4	11	0	0	0	0	0	0	0	0	0	0	0	0	0
**At2g42380**	E1	0	7	9	0	3	2	2	0	0	0	0	4	0	12	0	0	2
**At3g58120**	E1	0	27	97	43	0	4	5	0	2	1	7	0	0	2	2	0	0
**At2g16770**	F2	16	13	4	0	0	0	0	0	6	0	10	0	0	0	0	3	0
**At4g35040**	F2	103	279	64	375	132	13	15	26	14	238	14	9	34	20	40	26	77
**At4g01120**	G1	208	65	39	110	20	22	15	33	28	61	4	3	26	38	0	189	19
**At2g46270**	G1	0	1	0	9	4	0	3	5	3	0	0	0	0	0	0	0	4
**At1g32150**	G3	19	11	18	10	8	31	31	13	2	5	30	3	8	13	0	26	37
**At2g35530**	G3	84	52	15	20	21	22	35	26	34	20	26	1	26	17	9	7	41
**At1g43700**	I1	0	0	0	0	0	2	24	0	18	33	26	0	1	48	7	87	8
**At2g12900**	I1	0	0	0	0	8	0	0	0	0	0	0	0	0	0	0	0	0
**At2g31370**	I2	38	14	9	0	15	102	83	28	44	84	79	9	14	58	0	137	39
**At1g06070**	I2	50	31	27	26	32	3	5	12	12	0	0	0	1	12	2	0	11
**At1g06850**	I3	3	0	0	0	0	5	5	16	0	5	3	0	0	7	1	18	50
**At2g40620**	I3	7	6	0	36	0	4	3	2	0	11	28	0	0	0	0	0	1
**At2g21230**	I4	73	24	36	37	27	2	0	4	6	2	1	0	0	3	0	7	0
**At4g38900**	I4	62	31	30	25	61	12	37	21	19	20	6	3	0	17	0	60	29
**At1g68880**	S	0	0	3	52	0	0	0	1	0	20	0	0	0	0	0	0	0
**At5g49450**	S	16	5	45	35	5	1	19	6	0	44	4	14	1	192	13	41	9
**At3g49760**	S	0	0	0	8	0	0	0	0	0	0	0	0	0	0	0	0	0
**At3g30530**	S	6	6	3	0	3	0	0	0	0	0	0	0	0	3	0	0	0
**At1g75390**	S	47	146	0	88	130	7	2	8	5	35	23	0	0	0	1	9	11
**At5g38800**	S	0	0	0	0	17	0	0	3	3	0	0	0	0	0	0	0	16
**At5g15830**	S	0	0	16	84	0	0	0	0	0	73	0	0	0	12	0	0	0
**At4g37730**	S	0	0	0	0	0	0	0	0	0	16	1	0	0	0	0	0	0
**At4g34590**	S	21	153	7	24	68	20	6	9	17	21	11	0	0	9	8	0	122
**At1g13600**	S	0	0	0	2	0	2	0	0	0	0	0	0	0	0	0	0	0
**At2g18160**	S	0	0	99	132	80	109	184	43	82	458	92	37	28	84	128	0	120
**At2g04038**	S	0	0	0	4	0	0	0	0	0	0	0	5	0	0	0	0	0
**At3g62420**	S	455	103	238	252	69	12	55	98	30	96	149	11	153	84	22	119	43
