# Supplementary material for: The Role of bZIP Transcription Factors in Green Plant Evolution: Adaptive Features Emerging from Four Founder Genes
Source: PLoS One. 2008 Aug 13;3(8):e2944. doi: 10.1371/journal.pone.0002944 (PMC2492810; doi:10.1371/journal.pone.0002944)
Supplement: Text S1 — Supporting texts including further results and discussion. (0.06 MB DOC) [file pone.0002944.s032.doc]

# Supporting Information

#### Supporting Text S1a to S1h - Results and Discussion

**Supporting** **Text S1a - Groups of Homologues of Angiosperm *bZIP* Genes**

The set of rice *bZIP* genes was obtained by searching the sequences of the *O. sativa* ssp. *indica* and *japonica* genomes and full-length cDNAs and ESTs against an angiosperm bZIP data set (i.e., Angiotot) as reference. The sequences identified were compared against themselves, and those exhibiting an identity of less than 97% over their alignment were considered as representatives of two different loci; otherwise, only one representative was kept for further analysis. This criterion is based on the 96.5% amino acid identity level observed between two recent maize bZIP paralogues, OHP1 and OHP1b [1,2]. Of the 92 rice *bZIP* genes that we detected, 87 are common to both subspecies, and two and three genes respectively are exclusively present in *indica* or *japonica*.

Jakoby *et al.* [3] have previously clustered 75 bZIPs from Arabidopsis into ten groups based on sequence conservation in the basic region and on the presence of conserved motifs. To test whether this clustering could be extended to other angiosperm *bZIP* genes, thus defining groups of homologues among them, phylogenetic analyses were carried out. The only region allowing an alignment of all bZIPs was found to be restricted to the minimum bZIP domain, comprising the basic motif and three leucine heptads, totaling 44 amino acids, which is in accordance with previous results [4,5]. The Arabidopsis AtbZIP78 sequence was not used in these analyses as its leucine zipper contains only two leucine repetitions. The Arabidopsis *AtbZIP73* gene possesses an unexpected stop codon in its bZIP domain, we therefore considered it a pseudogene and renamed it *ΨAtbZIP73*.

**Supporting** **Text S1b –** **Lack of Introns in Group A *bZIP* Genes**

All genes except for *Gbf4* (*AtbZIP40*) and *AtbZIP13* from Group A share common intron positions. The absence of introns in *Gbf4* and *AtbZIP13* may indicate a retrogene process, whereby a cDNA copy of a gene is synthesized by RNA reverse transcription and inserted back into the genome, most likely through homologous recombination [6–8]. To which extent loss of introns through retrogene insertion has been active in shaping the Arabidopsis genome remains to be more thoroughly evaluated. Previous work has shown that introns in Arabidopsis have a tendency to become very short and, in some cases, even disappear [9].

**Supporting** **Text S1c - New bZIP Basic Motif**

The second half of the basic motif of Group L members (Figures 1A and S2), which is encoded by the second exon of this conserved region, appears to be five amino acids shorter than observed in all others bZARP members, thus representing an atypical basic motif. An otherwise highly conserved asparagine appears to align with an arginine or lysine residue, raising the possibility of altered DNA-binding specificity (Figure S2). Moreover, this region is also part of a bipartite nuclear localization signal (NLS) [10], but nuclear localization does not seem to be affected by these changes (A.B. Silveira and M. Vincentz, unpublished).

**Supporting** **Text S1d - Possible Groups of Orthologues (PoGOs) in Angiosperms***.*

Groups A, D and S include genes for responses and adaptation to environmental factors and the control of energy use and these groups are among the most amplified in the bZIP family. Our data are in accordance with the observation that genes of stress response pathways, e.g. genes encoding protein kinases, were more intensely duplicated and retained during angiosperm evolution than genes not directly involved in environmental adaptation [11]. On the other hand, the amplification of some Group S genes potentially contributed to more efficient energy use, which certainly represents a key element of growth optimization in unstable environments. Although higher capacity for tolerating environmental fluctuations represents a rather important selective advantage for sessile organism such as plants, the amplification of TF involved in developmental processes such as MIKC^c^-type MADS-box genes has also played an important role in the diversification of higher plants (MADS-box gene family in rice: genome-wide identification, organization and expression profiling during reproductive development and stress [12,13].

Changing the temporal or spatial gene expression patterns of paralogues may help to establish more efficient stress response strategies in plants [14–16]. Indeed, expression data for *bZIP* genes from rice (MPSS) and Arabidopsis (MPSS and GENEVESTIGATOR) showed that most of the paralogues posses distinct expression profiles, indicating that they are subject to processes of sub- or neo-functionalization (Dataset S3 and Dataset S4) [17]. Good examples for paralogous genes with distinct expression profiles are *TGA2* and *TGA6* in PoGO D5, which are paralogues that probably arose through a recent duplication of part of the Arabidopsis genome (Table S7). Although both genes participate in pathogen defense and share a high degree of functional redundancy, their expression patterns are divergent [18] indicating that fine-tuning of expression adds new functionally relevant attributes to genes. Micro- and macro-arrays are now common tools for genome-wide expression analyses. However, such arrays are often not suitable for the analysis of TF genes which are often expressed at a low level or a cell-specific manner. However, alternative platforms for sensitive expression profiling of weakly expressed genes have recently been developed, such as multi-parallel quantitative reverse-transcription polymerase chain reaction (qRT-PCR) for expression profiling of TF genes at a genome-wide scale in Arabidopsis and rice [19,20]. These new methods will enable us to precisely study the expression behavior of large sets of control genes, such as *bZIPs*, in different plant species (including algae). The information gained will certainly assist in assigning functions to new genes.

Additionally, the ability of certain bZIP factors to form heterodimers (e.g., from Groups S and C [21]) could promote functional diversification of *bZIP* genes similarly to what has been described for the B-class type-II MADS-box genes [22,23]**.**

The identification of multigene families and their organization in groups of orthologues is expected to facilitate the transfer of biochemical, structural and functional information between genes of the same PoGO thereby rationalizing gene characterization studies. For instance, in Group D, the Arabidopsis and wheat HBP-1 (Histone promoter-Binding Protein-1) transcription factor orthologues, present in PoGO D5, specifically bind to a *cis*-element found in promoters of histone H3 and H4 genes, possibly regulating their transcription [24,25]. Moreover, in the same PoGO, the paralogous transcription factors HBP-1, TGA2 and TGA6 from Arabidopsis act in the systemic defense pathway activated upon pathogen attack. Analysis of mutants of these genes indicated that they share some functional redundancy [18]. Orthologues of these genes in bean, potato and tobacco identified through the phylogenetic analysis presented here have functional relationships to pathogen defense, as expected [26–28].

Another example of function conservation concerns Group C, in which PoGO C1 possesses a subclade of monocot-specific paralogues, including genes like *Opaque-2*, *OHP1* and *OHP2* from maize (Figure S6) [2]. Maize Opaque-2 is a key regulator of carbon/nitrogen balance during seed development [29]. Monocot orthologues of *Opaque-2* exhibit endosperm-specific expression (*OsbZIP63*, *SPATa*, *BLZ2Hv*, *O2Sb*, *O2Zm* and *O2Clj*) [4,29–33]. This behavior appears to be monocot-specific, as expression of other *bZIP* genes from Group C is not restricted to endosperm tissue. Phylogenetic analysis showed that the most likely Arabidopsis orthologue of *Opaque-2* is *Bzo2h3/AtbZIP63*. Functional analysis showed that BZO2H3 protein does not bind the promoter of *AtS2*, which is a reserve protein gene [34]. However, it has recently been shown that BZO2H3 is a key regulator of energy homeostasis in response to glucose availability thus suggesting that it has a role in carbon/nitrogen balance control [35]. The inference of functional characteristics among groups of orthologues can improve greatly the transfer of functional information. Nevertheless, non-orthologous functional conservation might represent an additional challenge [36].

Moreover, the different examples described here indicate that expression data may give some important clue about functional conservation but do not always constitute a conclusive or reliable feature. In broader terms, precise functional studies of orthologous or even homologous groups of TFs of plant model organisms (algae, Physcomitrella, Arabidopsis, rice) encompassing the main evolutionary steps of green plants should be undertaken to thoroughly evaluate the functional conservation of TFs and their associated regulatory networks. Reverse genetic approaches (random or specific gene disruption, overexpression of strong activators or repressors or their inducible counterparts) in combination with microarray technology to detect target genes, the identification of interacting partners and even metabolomic analyses should pave the way towards a better understanding of the functional diversification of plant TFs. Simultaneous silencing by artificial miRNA or dsRNA of several paralogous or related homologues (sister clades) should also be considered to overcome functional redundancy in more complex genomes.

**Supporting** **Text S1e – Conserved One-to-One Relationships**

Another interesting feature that can be deduced from our analysis is the presence of genes that do not have any paralogous relationship inside their respective PoGOs. PoGO K1 contains only a single gene from each of the three model lineages (Figure 3). This one-to-one relationship indicates that these genes are under evolutionary constraint that limits their duplication (amplification) perhaps indicating an important function [11].

As eudicots and monocots have diverged earlier than lineages inside eudicots, it was quite unexpected to find four PoGOs (C1, D4, G2 and H2) with an Arabidopsis versus rice one-to-one relationship, but with two black cottonwood genes each. In two cases (D4 and H2) the black cottonwood paralogues are located in genomic regions that underwent a recent genome-wide duplication, ~60 million years ago [37,38]. A possible explanation for the divergence from the one-to-one relationship is that black cottonwood is more tolerant than Arabidopsis and rice towards the duplication of key genes. Alternatively, it is possible that black cottonwood has undergone a slower rate of sequence evolution than Arabidopsis or rice. Three factors may have contributed to this: (i) black cottonwood has a long generation time (4 to 8 years) [39], whereas Arabidopsis and rice can pass through up to three or four generations per year; (ii) the molecular clock of black cottonwood has been calculated to be six times slower than that of Arabidopsis [38]; and (iii), as black cottonwood is a long-living plant with pronounced vegetative propagation, the contribution of old and conserved genotypes to current black cottonwood genomes is therefore probably high, thus reducing the overall rate of sequence evolution [38,40]. It is therefore tentative to speculate that the rate of sequence elimination is lower in black cottonwood and the corollary of this possibility is that duplicated genes would have a longer half-life. This hypothesis can now be explored at the whole-genome scale.

**Supporting** **Text S1f to S1h - Tracing the Origin and Diversification of *bZIP* Genes in Green Plants.**

**Supporting** **Text S1f - ABA Signaling in Physcomitrella**

The Physcomitrella *PpLEA1* gene is responsive to osmotic stress and ABA treatment [41]. Kamisugi *et al*. have shown that the promoter of its wheat homologue, the *Em* gene, is ABA-activated when transferred to the moss, whereas the *PpLEA1* promoter is not ABA-inducible in cereals. This observation suggests that the ABA regulatory networks of Physcomitrella and wheat are partly different, possibly reflecting an ancestral and more rudimentary ABA signaling cascade in the moss [41]. In angiosperms, this network involves an ABI3/VP1 transcription factor and a bZIP. Recently, Marella *et al*. [42] showed that PpABI3A, an ABI3/VP1 transcription factor orthologue from Physcomitrella, plays a role similar to that of ABI3/VP1 proteins in angiosperms. However, PpABI3A did not fully reverse the phenotype of the Arabidopsis *abi3-6* mutant, probably indicating weak interaction between PpABI3A and the Arabidopsis ABI5 bZIP transcription factor, which is an important partner of ABI3 in seed plants. Notably, ABI5 belongs to Group A, thus it is not present in mosses (Figure 2). Additionally, as reported for Arabidopsis, ABI3 can interact with bZIPs of Group C [34]. Possibly, the weak interaction seen between PpABI3A and ABI5 reflects a partially unspecific binding of the former to bZIPs and raises the question of which bZIP, if any, interacts with PpABI3A and participates in ABA-mediated responses in Physcomitrella. The emergence of Group A in gymnosperms perhaps led to the tighter interaction of ABI3/VP1 and bZIPs of this group establishing new networks of ABA signaling, mediated by ABI3. These new networks might be important to cope in more efficient ways with novelties developed in seed plants, e.g., longer distances and times for signaling processes due to higher trunks, which are absent in mosses.

**Supporting** **Text S1g *-*****A Possible Group of Homologues in Non-Angiosperms**

To exclude the possibility that the absence of angiosperm bZIPs in Group NA was caused by a systematic bias (e.g., large number of sequences analyzed leading to low bootstrap values) and to obtain more information about the relationship between algal and terrestrial plant bZIPs, we performed two additional analyses. In one phylogeny we included Arabidopsis and all bryophyte sequences, and in the second study we included bZIPs from bryophytes and algae. The Arabidopsis/bryophyte analysis confirmed the group pattern that we observed for sequences of the ViridiZIP set (Figure S18), indicating that this approach had sufficient resolution and confirming that Arabidopsis sequences were absent from Group NA. In the bryophyte/algae study all groups of homologues including bryophyte bZIPs were recovered (Groups B, C, D, E, F, G, H, I, J). Moreover, evidence for the existence of various sister groups, such as Groups B and H, Groups D and F, Groups E and I, and Groups G and J, was obtained (Figure 1B). This result is consistent with the branching pattern inferred from angiosperm sequences (Figure 1A) and therefore supports the results obtained through the analysis of bryophyte/algae bZIPs.

**Supporting** **Text S1h -** **bZIP Evolution in Plants, Pseudogenes**

We also searched for putative *bZIP* pseudogenes and found 12 candidates in Arabidopsis, 23 in rice, 22 in black cottonwood, five in Chlamydomonas and one in Ostreococcus. Formation of pseudogenes is one of the processes leading to gene loss. Except for black cottonwood, where pseudogenes tend to be derived from Group D (31%), there is no clear tendency for gene loss in the other organisms analyzed. The pattern of gene gain and loss detected in our analysis is compatible with the general birth-and-death model of gene family evolution [43,44].

Reference List

1. Pysh LD, Aukerman MJ, Schmidt RJ (1993) OHP1: a maize basic domain/leucine zipper protein that interacts with opaque2. Plant Cell 5: 227-236.

2. Vincentz M, Bandeira-Kobarg C, Gauer L, Schlogl P, Leite A (2003) Evolutionary pattern of angiosperm bZIP factors homologous to the maize Opaque2 regulatory protein. J Mol Evol 56: 105-116.

3. Jakoby M, Weisshaar B, Droge-Laser W, Vicente-Carbajosa J, Tiedemann J *et al.* (2002) bZIP transcription factors in Arabidopsis. Trends Plant Sci 7: 106-111.

4. Vettore AL, Yunes JA, Cord NG, da Silva MJ, Arruda P *et al.* (1998) The molecular and functional characterization of an Opaque2 homologue gene from Coix and a new classification of plant bZIP proteins. Plant Mol Biol 36: 249-263.

5. Vincentz M, Cara FA, Okura VK, da Silva FR, Pedrosa GL *et al.* (2004) Evaluation of monocot and eudicot divergence using the sugarcane transcriptome. Plant Physiol 134: 951-959.

6. Graur D, Li WH (2000) Fundamentals of Molecular Evolution. Sinauer Associates, Inc., Publishers.MA. U.S.A).

7. Sosinsky A, Glusman G, Lancet D (2000) The genomic structure of human olfactory receptor genes. Genomics 70: 49-61.

8. Knowles DG, McLysaght A (2006) High rate of recent intron gain and loss in simultaneously duplicated Arabidopsis genes. Mol Biol Evol 23: 1548-1557.

9. Seoighe C, Gehring C, Hurst LD (2005) Gametophytic selection in *Arabidopsis thaliana* supports the selective model of intron length reduction. PLoS Genet 1: e13.

10. Varagona MJ, Raikhel NV (1994) The basic domain in the bZIP regulatory protein Opaque2 serves two independent functions: DNA binding and nuclear localization. Plant J 5: 207-214.

11. Shiu SH, Karlowski WM, Pan R, Tzeng YH, Mayer KF *et al.* (2004) Comparative analysis of the receptor-like kinase family in Arabidopsis and rice. Plant Cell 16: 1220-1234.

12. Arora R, Agarwal P, Ray S, Singh AK, Singh VP *et al.* (2007) MADS-box gene family in rice: genome-wide identification, organization and expression profiling during reproductive development and stress. BMC Genomics 8: 242.

13. Quodt V, Faigl W, Saedler H, Munster T (2007) The MADS-domain protein PPM2 preferentially occurs in gametangia and sporophytes of the moss Physcomitrella patens. Gene 400: 25-34.

14. Ha M, Li WH, Chen ZJ (2007) External factors accelerate expression divergence between duplicate genes. Trends Genet 23: 162-166.

15. Li WH, Yang J, Gu X (2005) Expression divergence between duplicate genes. Trends in Genetics 21: 602-607.

16. Gu ZL, Rifkin SA, White KP, Li WH (2004) Duplicate genes increase gene expression diversity within and between species. Nat Genet 36: 577-579.

17. Akhunov ED, Akhunova AR, Dvorak J (2007) Mechanisms and rates of birth and death of dispersed duplicated genes during the evolution of a multigene family in diploid and tetraploid wheats. Mol Biol Evol 24: 539-550.

18. Zhang Y, Tessaro MJ, Lassner M, Li X (2003) Knockout analysis of Arabidopsis transcription factors TGA2, TGA5, and TGA6 reveals their redundant and essential roles in systemic acquired resistance. Plant Cell 15: 2647-2653.

19. Czechowski T, Bari RP, Stitt M, Scheible WR, Udvardi MK (2004) Real-time RT-PCR profiling of over 1400 Arabidopsis transcription factors: unprecedented sensitivity reveals novel root- and shoot-specific genes. Plant J 38: 366-379.

20. Caldana C, Scheible WR, Mueller-Roeber B, Ruzicic S (2007) A quantitative RT-PCR platform for high-throughput expression profiling of 2500 rice transcription factors. Plant Methods 3: 7.

21. Ehlert A, Weltmeier F, Wang X, Mayer CS, Smeekens S *et al.* (2006) Two-hybrid protein-protein interaction analysis in Arabidopsis protoplasts: establishment of a heterodimerization map of group C and group S bZIP transcription factors. Plant J 46: 890-900.

22. Hernandez-Hernandez T, Martinez-Castilla LP, Alvarez-Buylla ER (2007) Functional diversification of B MADS-box homeotic regulators of flower development: Adaptive evolution in protein-protein interaction domains after major gene duplication events. Mol Biol Evol 24: 465-481.

23. Veron AS, Kaufmann K, Bornberg-Bauer E (2007) Evidence of interaction network evolution by whole-genome duplications: a case study in MADS-box proteins. Mol Biol Evol 24: 670-678.

24. Kawata T, Minami M, Tamura T, Sumita K, Iwabuchi M (1992) Isolation and characterization of a cDNA clone encoding the TATA box-binding protein (TFIID) from wheat. Plant Mol Biol 19: 867-872.

25. Mikami K, Sakamoto A, Iwabuchi M (1994) The HBP-1 family of wheat basic/leucine zipper proteins interacts with overlapping *cis*-acting hexamer motifs of plant histone genes. J Biol Chem 269: 9974-9985.

26. Feltkamp D, Masterson R, Starke J, Rosahl S (1994) Analysis of the involvement of ocs-like bZip-binding elements in the differential strength of the bidirectional mas1'2' promoter. Plant Physiol 105: 259-268.

27. Niggeweg R, Thurow C, Kegler C, Gatz C (2000) Tobacco transcription factor TGA2.2 is the main component of as-1-binding factor ASF-1 and is involved in salicylic acid- and auxin-inducible expression of as-1-containing target promoters. J Biol Chem 275: 19897-19905.

28. Tucker ML, Whitelaw CA, Lyssenko NN, Nath P (2002) Functional analysis of regulatory elements in the gene promoter for an abscission-specific cellulase from bean and isolation, expression, and binding affinity of three TGA-type basic leucine zipper transcription factors. Plant Physiol 130: 1487-1496.

29. Schmidt RJ, Burr FA, Aukerman MJ, Burr B (1990) Maize regulatory gene opaque-2 encodes a protein with a "leucine-zipper" motif that binds to zein DNA. Proc Natl Acad Sci U S A 87: 46-50.

30. Onodera Y, Suzuki A, Wu CY, Washida H, Takaiwa F (2001) A rice functional transcriptional activator, RISBZ1, responsible for endosperm-specific expression of storage protein genes through GCN4 motif. J Biol Chem 276: 14139-14152.

31. Albani D, Hammond-Kosack MC, Smith C, Conlan S, Colot V *et al.* (1997) The wheat transcriptional activator SPA: a seed-specific bZIP protein that recognizes the GCN4-like motif in the bifactorial endosperm box of prolamin genes. Plant Cell 9: 171-184.

32. Onate L, Vicente-Carbajosa J, Lara P, Diaz I, Carbonero P (1999) Barley BLZ2, a seed-specific bZIP protein that interacts with BLZ1 *in vivo* and activates transcription from the GCN4-like motif of B-hordein promoters in barley endosperm. J Biol Chem 274: 9175-9182.

33. Pirovano L, Lanzini S, Hartings H, Lazzaroni N, Rossi V *et al.* (1994) Structural and functional analysis of an Opaque-2-related gene from sorghum. Plant Mol Biol 24: 515-523.

34. Lara P, Onate-Sanchez L, Abraham Z, Ferrandiz C, Diaz I *et al.* (2003) Synergistic activation of seed storage protein gene expression in Arabidopsis by ABI3 and two bZIPs related to OPAQUE2. J Biol Chem 278: 21003-21011.

35. Baena-Gonzalez E, Rolland F, Thevelein JM, Sheen J (2007) A central integrator of transcription networks in plant stress and energy signalling. Nature 448: 938-943.

36. Causier B, Castillo R, Zhou J, Ingram R, Xue Y *et al.* (2005) Evolution in action: following function in duplicated floral homeotic genes. Curr Biol 15: 1508-1512.

37. Jansson S, Douglas CJ (2007) Populus: A Model System for Plant Biology. Annu Rev Plant Biol 58: 435-458.

38. Tuskan GA, Difazio S, Jansson S, Bohlmann J, Grigoriev I *et al.*(2006) The genome of black cottonwood, *Populus trichocarpa* (Torr. & Gray). Science 313: 1596-1604.

39. James RR, Croft BA, Strauss SH (1999) Susceptibility of the cottonwood leaf beetle (Coleoptera : Chrysomelidae) to different strains and transgenic toxins of Bacillus thuringiensis. Environ Entomol 28: 108-115.

40. Mitton JB, Grant MC (1996) Genetic variation and the natural history of quaking aspen. BioScience 46: 25-31.

41. Kamisugi Y, Cuming AC (2005) The evolution of the abscisic acid-response in land plants: comparative analysis of group 1 *LEA* gene expression in moss and cereals. Plant Mol Biol 59: 723-737.

42. Marella HH, Sakata Y, Quatrano RS (2006) Characterization and functional analysis of ABSCISIC ACID INSENSITIVE3-like genes from *Physcomitrella patens*. Plant J 46: 1032-1044.

43. Nei M, Rooney AP (2005) Concerted and birth-and-death evolution of multigene families. Annu Rev Genet 39: 121-152.

44. Hahn MW, De Bie T, Stajich JE, Nguyen C, Cristianini N (2005) Estimating the tempo and mode of gene family evolution from comparative genomic data. Genome Res 15: 1153-1160.
